# Efficient prime editing in vivo and in vitro using lipid nanoparticles

**DOI:** 10.1038/s41565-026-02200-6

**Published:** 2026-06-15

**Authors:** Allen Y. Jiang, Ana Cristian, Dominique L. Brooks, Emily R. Feierman, Paul Z. Chen, Madelynn N. Whittaker, Sarah E. Pierce, Holt A. Sakai, Hongyu Chen, Dangliang Liu, Peyton B. Randolph, Angus H. Li, Alvin Hsu, Serena O. Omo-Lamai, Y. Allen Tao, Benista Owusu-Amo, Xiao Wang, Xiao Wang, Kiran Musunuru, David R. Liu

**Affiliations:** 1 Merkin Institute of Transformative Technologies in Healthcare, Broad Institute of MIT and Harvard, Cambridge, MA USA; 2 Department of Chemistry and Chemical Biology, Harvard University, Cambridge, MA USA; 3 Howard Hughes Medical Institute, Harvard University, Cambridge, MA USA; 4 Institute for Medical Engineering and Sciences, Massachusetts Institute of Technology, Cambridge, MA USA; 5 Cardiovascular Institute, Perelman School of Medicine at the University of Pennsylvania, Philadelphia, PA USA; 6 Division of Cardiovascular Medicine, Department of Medicine, Perelman School of Medicine at the University of Pennsylvania, Philadelphia, PA USA; 7 Department of Genetics, Perelman School of Medicine at the University of Pennsylvania, Philadelphia, PA USA; 8 Koch Institute for Integrative Cancer Research, Massachusetts Institute of Technology, Cambridge, MA USA; 9 Department of Chemistry, Massachusetts Institute of Technology, Cambridge, MA USA; 10 Broad Institute of MIT and Harvard, Cambridge, MA USA; 11 Harvard John A. Paulson School of Engineering and Applied Sciences, Harvard University, Cambridge, MA USA; 12 Stanley Center for Psychiatric Research, Broad Institute of MIT and Harvard, Cambridge, MA USA

**Keywords:** Nanoparticles, Drug delivery

## Abstract

Prime editing is a versatile clinical genome editing method that enables precise substitutions, small insertions and deletions at specified locations in the genomes of living systems including human cells. Although non-viral lipid nanoparticle (LNP) delivery of RNA in vivo has become a preferred method for gene editing in animals and patients, its application to complex, three-component prime editing systems has yielded low editing efficiencies. Here we developed a systematic prime editing LNP (PE-LNP) optimization platform that addresses key bottlenecks in cargo design that limit editing efficiency. This generalizable workflow yielded PE-LNPs that can achieve 49% average in vivo prime editing in the bulk mouse liver with a single dose of 2 mg kg^−1^. We applied our workflow to the correction of *PAH* R408W, a cause of phenylketonuria, in a mouse model and achieved prime editing efficiencies and serum phenylalanine levels anticipated to be curative. We also show that PE-LNPs minimize off-target editing compared with DNA delivery methods, induce only transient elevation of liver enzymes and can be dosed repeatedly to improve editing efficiencies. These PE-LNP systems provide an attractive alternative to viral delivery by offering transient expression that minimizes off-target editing, no observed long-term toxicity and high levels of non-viral in vivo liver prime editing.

## Abstract (web summary)

A systematic workflow is used to optimize lipid nanoparticle-based prime editing systems that enable efficient editing and disease correction in human cells and mouse tissue, and overcome key limitations of transient delivery.

## Main

The versatility of prime editing, which can make virtually any local change at a targeted site in the genome of living cells^1^, has enabled the treatment of a range of diverse genetic diseases, including chronic granulomatous disease^2^, sickle cell disease^3,4^, alternating hemiplegia of childhood^5^, retinitis pigmentosa^6,7^, Leber congenital amaurosis^8,9^, phenylketonuria (PKU)^10–12^, hereditary tyrosinemia^8^ and alpha-1 antitrypsin deficiency^13^ in animal models or in human patients. Therapeutic applications of prime editing so far, however, have primarily relied on ex vivo electroporation of prime editors (PEs) followed by engraftment, or on in vivo viral delivery. Although viral delivery methods including the use of adeno-associated viruses (AAVs) are capable of potent in vivo delivery of PEs, they have limited packaging capacity, can elicit immune responses preventing redosing and may result in prolonged transgene expression that increases opportunities for off-target editing or immunogenicity. Non-viral in vivo delivery vehicles capable of overcoming these challenges while supporting efficient prime editing of target genes would substantially expand the applicability of in vivo prime editing for research and therapeutic applications.

Lipid nanoparticles (LNPs) are a promising non-viral delivery modality and have already mediated therapeutic in vivo delivery of mRNA encoding Cas9 nucleases^14^ and base editors (BE)^15–17^ in animals and in human patients. The first in vivo correction of a pathogenic mutation in a human was recently achieved using a BE-LNP targeting the most common cause of alpha-1 antitrypsin deficiency, a serious liver and lung disease^16^. More recently, baby K. J. Muldoon, a patient with a life-threatening urea cycle disorder, was effectively treated with a personalized BE-LNP^15^. These clinical outcomes highlight the utility and safety of LNP-mediated delivery of gene editing agents.

In contrast to AAVs, LNPs are entirely synthetic and typically consist of four lipid components: an ionizable lipid, a helper phospholipid, a polyethylene glycol (PEG) lipid and cholesterol (Chol). By tuning the identity and ratios of these components, LNPs can be produced with specific characteristics such as transfection potency, tissue tropism, circulation lifetime and inflammatory response^18,19^. When administered systemically, LNPs generally traffic to the liver because of ApoE opsonization and the fenestrated vasculature of the liver, where they are preferentially uptaken by hepatocytes via low-density lipoprotein receptor-mediated endocytosis^20,21^. LNPs are thus an ideal vehicle for delivery of gene editing agents to the liver.

Despite successful LNP delivery of gene therapies and gene editing agents, researchers have reported limited in vivo success for LNP-mediated delivery of prime editing systems due to low editing efficiency^12,22,23^. Given the complexity of prime editing and the numerous steps it requires, prime editing mediated by LNPs could be limited by many potential bottlenecks. First, a temporal mismatch between the PE protein, which must be translated from the delivered mRNA, and the short-lived synthetic PE guide RNA (pegRNA), may cause pegRNA availability in the cell to be limiting by the time the PE is present at a useful concentration^24^. Several previous efforts have thus sought to enhance pegRNA abundance. Highly chemically modified pegRNAs (HM-pegRNAs) containing stabilizing 2′-O-methyl (2′-OMe) substitutions in the SpCas9 guide RNA (gRNA) scaffold have been used in an attempt to overcome this limitation^22^ but yielded only 8% average bulk liver editing in mice following a regimen of three weekly 3 mg kg^−1^ LNP doses. Constitutive, hepatocyte-specific engineered pegRNA (epegRNA) expression using AAV9^12^ in combination with LNP-delivered PE mRNA resulted in an average of 16% mouse bulk liver editing but required the use of viral delivery of epegRNA. Finally, LNP delivery of PE fused to the small RNA-binding protein La^25^ (referred to as PE7) along with La-accessible pegRNAs achieved up to 23% mouse bulk liver editing following two 4 mg kg^−1^ doses of PE-LNP components and represents the current state of the art among published PE-LNP methods^12^.

Second, suboptimal PE activity and translation could limit prime editing efficiency in vivo, effects that would be compounded by the transience of editor production from mRNA. Recently, a PE designed to improve reverse transcriptase solubility and dNTP affinity increased editing efficiency in the mouse liver following LNP delivery by 1.7-fold compared with PEmax, but still achieved only 1.8% editing^23^. Third, stoichiometric mismatch of the prime editing components when delivered by LNPs could result in reduced editing efficiency if one of the components becomes limiting in a transient delivery context, disrupting coordinated reaction steps necessary for successful prime editing.

These bottlenecks are compounded by a substantial nanoparticle engineering challenge. The PE-LNP system must deliver three physicochemically distinct cargoes: a PE mRNA approximately 6.5 kb in length and two much smaller gRNAs of approximately 100–150 nucleotides. This complexity, combined with the need for stoichiometric control to ensure coordinated function, presents a considerable challenge for LNP formulation and effective delivery of the prime editing components for in vivo genome editing.

In this work, we develop an all-RNA PE-LNP system capable of efficient prime editing in the liver by identifying multiple bottlenecks in editing efficiency and overcoming them through iterative optimization. We explored the use of state-of-the-art prime editing systems^26^, novel 3′ epegRNA motifs^27^ and varying ratios of three prime editing components to yield a formulation-agnostic and site-agnostic workflow for PE-LNP optimization. Our best-performing PE-LNP system is capable of supporting 49% indel-free prime editing in bulk mouse liver at the *Pcsk9* locus after a single 2 mg kg^−1^ dose, a 63-fold improvement in editing efficiency at the same dose compared with our initial approach. Using this optimized PE-LNP system, we achieved curative levels (15% of bulk liver) of in vivo prime editing in the *PAH* R408W humanized mouse model of PKU. Overall, this work provides a general starting point for efficient and precise LNP-mediated prime editing of the liver, including applications to treat genetic liver diseases, and advances our understanding of transient in vivo prime editing delivery.

## Iterative optimization of prime editing cargo for PE-LNPs

**Fig. 1:**
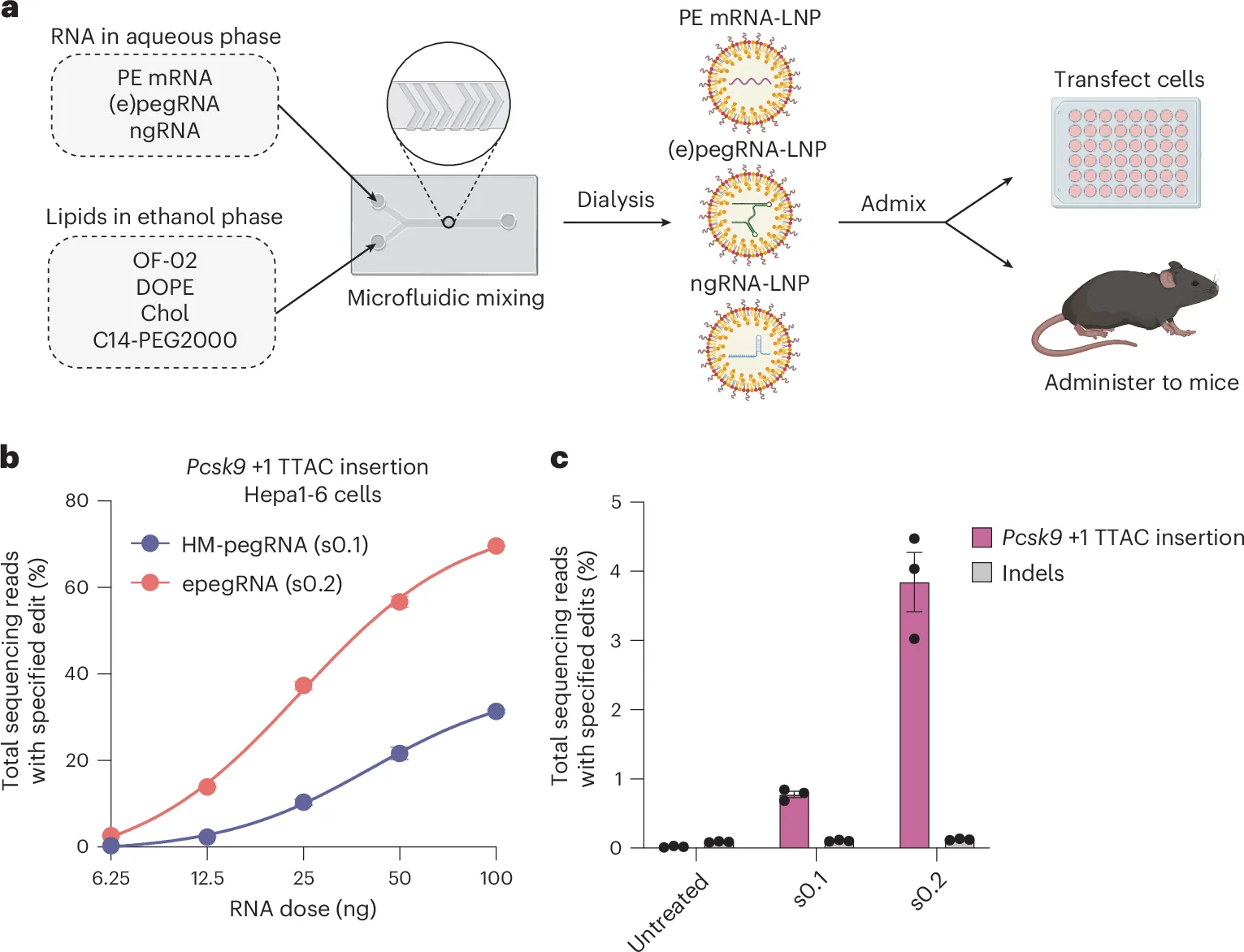
Initial development of PE-LNPs for prime editing in tissue culture and mice. **a**, Schematic of PE-LNP formulation and administration. LNPs are formulated to individually encapsulate PE mRNA, (e)pegRNA or ngRNA and admixed immediately before in vitro transfection or in vivo RO injection. **b**, Prime editing efficiencies of PE-LNPs encapsulating either HM-pegRNA (s0.1 PE-LNP) or epegRNA (s0.2 PE-LNP) for the *Pcsk9* +1 TTAC insertion in Hepa1-6 cells. Cells were collected after 72 h and analysed by HTS. Data were fitted to four-parameter logistic curves using nonlinear regression and represent the mean prime editing efficiencies ± s.e.m. of *n* = 3 wells. **c**, Prime editing efficiencies in the bulk liver of 6-week-old female C57BL/6 mice dosed via RO injection with s0.1 or s0.2 PE-LNPs delivered at a 2 mg kg^−1^ total RNA dose (1:0.9:0.1, mRNA:(e)pegRNA:ngRNA). Tissue from livers was collected 7 days after the injection and analysed by HTS. Data points represent individual mice, and error bars represent mean ± s.e.m. of *n* = 3 mice. Illustrations in **a** created in BioRender; Jiang, A. https://biorender.com/0xtxw71 (2026). Source data

Because a low intracellular concentration of synthetic pegRNA after PE translation may limit prime editing following LNP delivery, we initially sought to compare LNP-mediated prime editing efficiencies using either a synthetic HM-pegRNA, as previously described^22^, or a synthetic epegRNA containing a 3′ pseudoknot motif that protects the pegRNA from degradation^24^. To deliver prime editing components via LNPs, we used microfluidic mixing to separately formulate nucleoside-modified mRNA encoding PEmax, (e)pegRNA and nicking gRNA (ngRNA) into OF-02 LNPs (Fig. 1a). The OF-02 LNP formulation was chosen for its ability to efficiently deliver mRNA to the liver^28^. Although we considered coformulating the (e)pegRNA and ngRNA owing to their similar size and structure, we chose to formulate them separately to provide better control of individual doses and streamline the formulation process when testing different combinations of mRNA, (e)pegRNA and ngRNA.

We admixed LNPs individually containing the editor mRNA, (e)pegRNA or ngRNA immediately before either treating Hepa1-6 cells with increasing doses of PE-LNPs or retro-orbitally (RO) administering 2 mg kg^−1^ PE-LNPs to adult C57BL/6 mice. The LNPs were mixed at an initial ratio of 1:0.9:0.1 mRNA:(e)pegRNA:ngRNA by total RNA mass based on previous work demonstrating efficient editing in vitro with this ratio^29^. Using a previously developed PE3 strategy to install a +1 TTAC frameshift insertion at *Pcsk9*^22^, we observed in vitro editing efficiencies of 31% using HM-pegRNA and 70% using epegRNA at the highest dose of PE-LNPs tested (Fig. 1b). In the bulk liver of mice 1 week after LNP administration, we also observed only 0.8% average prime editing efficiency at *Pcsk9* when using HM-pegRNA (Fig. 1c, stage 0.1 (s0.1)). Bulk liver editing significantly increased to 3.8% when using epegRNA instead of HM-pegRNA (*P* = 0.0003) (Fig. 1c). Taken together, these results indicate that epegRNAs improve prime editing efficiencies over chemically modified pegRNAs in both cells and mouse liver following LNP delivery. PE-LNPs using epegRNA are hereafter referred to as stage 0.2 (s0.2) PE-LNPs.

**Fig. 2:**
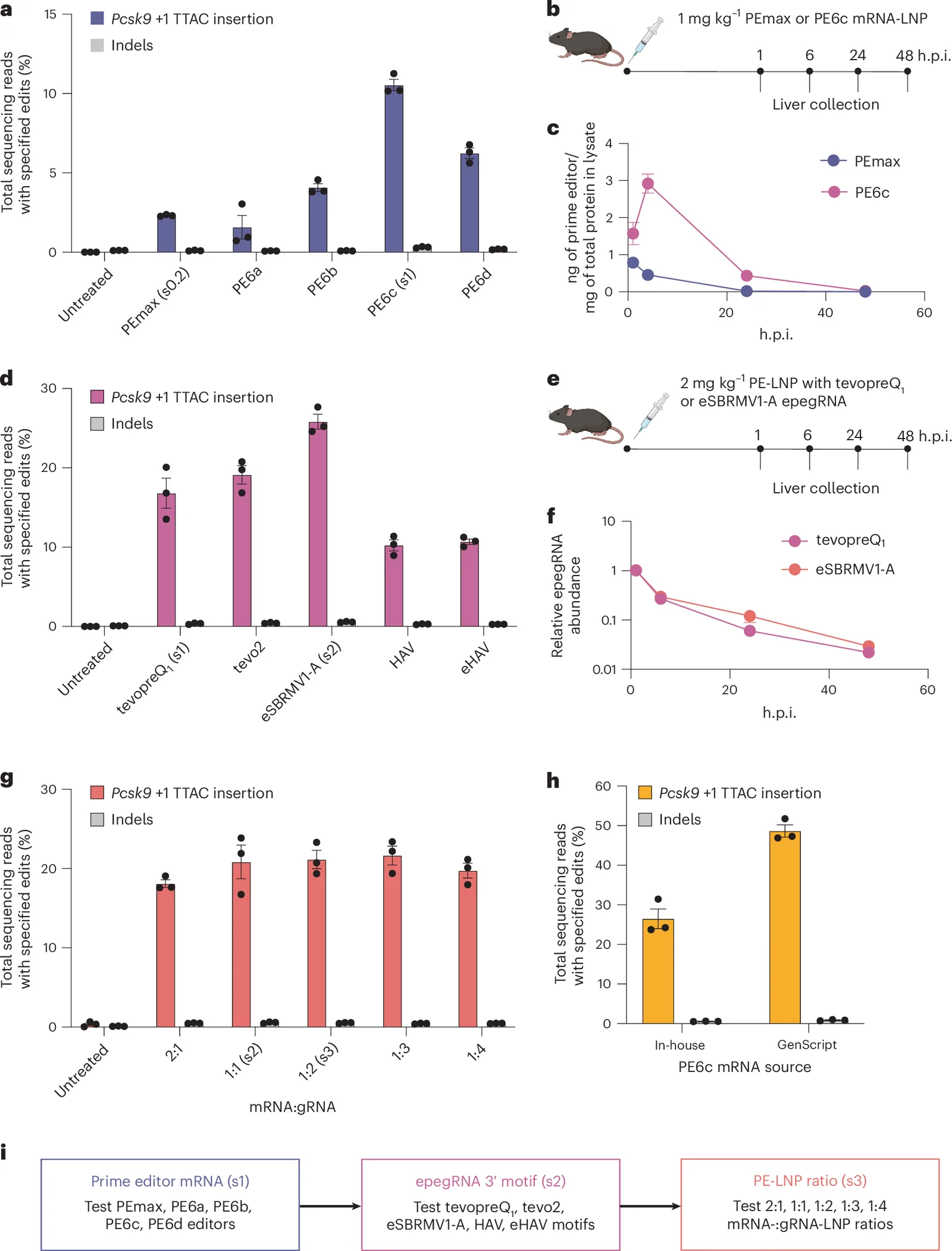
Identification of factors limiting efficient LNP-mediated prime editing in vivo. **a**, Prime editing efficiencies in the bulk liver of 6-week-old female C57BL/6 mice dosed via RO injection with s0.2 PE-LNPs encapsulating either PEmax or PE6 variant mRNA (PE6a through PE6d). PE-LNPs were delivered at 2 mg kg^−1^ total RNA dose (1:0.9:0.1, mRNA:epegRNA:ngRNA). Tissue from livers was collected 7 days after the injection and analysed by HTS. **b**, Schematic of dosing and liver collection at 1, 6, 24 and 48 h post-injection (h.p.i.) for analysis of PE protein abundance in the liver. **c**, Concentration of PE (PEmax or PE6c) protein as measured by ELISA in bulk liver lysate following injection of 1 mg kg^−1^ PE mRNA-LNP. **d**, Prime editing efficiencies in the bulk liver of 6-week-old female C57BL/6 mice dosed via RO injection with s1 PE-LNPs encapsulating epegRNA with either a 3′ tevopreQ_1_ motif or recently developed 3′ motifs. PE-LNPs were delivered at a 2 mg kg^−1^ total RNA dose (1:0.9:0.1, mRNA:epegRNA:ngRNA). Tissue from livers was collected 7 days after the injection and analysed by HTS. **e**, Schematic of dosing and liver collection at 1, 6, 24 and 48 h.p.i. for analysis of epegRNA abundance and prime editing in the liver. **f**, Relative abundance of epegRNA with either a tevopreQ_1_ or eSBRMV1-A 3′ motif in bulk liver lysate following a 2 mg kg^−1^ PE-LNP injection. epegRNA abundance at different timepoints following injection was measured by RT–qPCR and normalized to respective epegRNA abundance at 1 h. **g**, Prime editing efficiencies in the bulk liver of 6-week-old female C57BL/6 mice dosed via RO injection with s2 PE-LNPs. PE-LNPs were delivered at a 2 mg kg^−1^ total RNA dose with varied ratios of mRNA-LNP:gRNA-LNP (constant ratio of 9:1 for epegRNA-LNP:ngRNA-LNP). Tissue from livers was collected 7 days after the injection and analysed by HTS. **h**, Prime editing efficiencies in the bulk liver of 6-week-old female C57BL/6 mice dosed via RO injection with s3 PE-LNPs encapsulating PE6c mRNA produced either in-house or by GenScript. PE-LNPs were delivered at a 2 mg kg^−1^ total RNA dose (1:1.8:0.2, mRNA:epegRNA:ngRNA). Tissue from livers was collected 7 days after the injection and analysed by HTS. **i**, Workflow for the systematic optimization of PE-LNPs. For **a**, **d**, **g** and **h**, data points represent individual mice and error bars represent mean ± s.e.m. of *n* = 3 mice. For **c** and **f**, data points represent mean of *n* = 3 mice and error bars represent s.e.m. Illustrations in **b** and **e** created in BioRender; Jiang, A. https://biorender.com/ooa37g6 (2026). Source data

Delivery of LNPs encapsulating PE-encoding mRNA results in transient production of PE protein. While transient exposure to gene editing agents is helpful to minimize the risk of off-target editing and genotoxicity^9,30–33^, it also imposes a temporal constraint on the opportunity for on-target editing, which can substantially decrease prime editing efficiency. We hypothesized that the recently developed PE6 PEs^26^, which demonstrated enhanced activity and improved editing efficiency in the mouse brain when delivered by AAV, may also improve LNP-mediated prime editing in vivo. We delivered 2 mg kg^−1^ s0.2 PE-LNPs (1:0.9:0.1 mRNA:epegRNA:ngRNA by total RNA mass) encapsulating either PEmax, PE6a, PE6b, PE6c or PE6d mRNA to adult C57BL/6 mice via RO injection and evaluated prime editing of the *Pcsk9* +1 TTAC insertion. We observed improved average editing efficiencies in the bulk liver with PE-LNPs using PE6b (4.1% prime editing), PE6c (11%) and PE6d (6.2%) compared with PEmax (2.3%); PE6c in particular achieved a 4.6-fold improvement in editing over PEmax (*P* < 0.0001) (Fig. 2a). We used PE6c in subsequent PE-LNP optimization of the *Pcsk9* +1 TTAC insertion. PE-LNPs using a PE6 editor are hereafter referred to as s1 PE-LNPs.

We next sought to evaluate whether the improved editing in mice at *Pcsk9* observed with the use of PE6c compared with PEmax may be due to differences in translation of the respective PE in vivo. To test differences in editor production, we delivered 1 mg kg^−1^ of either PEmax or PE6c mRNA-LNPs to adult C57BL/6 mice via RO injection and collected livers 1, 6, 24 and 48 h after injection to quantify editor concentration in liver lysate (Fig. 2b). At all timepoints evaluated, we observed substantially greater concentrations of PE6c protein compared with PEmax as measured by enzyme-linked immunosorbent assay (ELISA) in liver lysate (Fig. 2c). This result suggests that, in addition to the previously characterized improved activity of PE6c over PEmax^26^, higher levels of prime editing observed at *Pcsk9* may also be partially due to improved production of the PE6c PE compared with PEmax. While we hypothesized that the smaller size of PE6c may be advantageous as smaller mRNAs encapsulate more efficiently^34^, attempts at mRNA size minimization did not yield improvements to prime editing in mice (Supplementary Note 1 and Extended Data Fig. 3). We therefore continued to use mRNA encoding full-length PE.

Next, we assessed whether newly developed 3′ epegRNA motifs beyond tevopreQ_1_ could further enhance in vivo editing efficiencies. These new 3′ motifs were mined from natural pseudoknots with diverse phylogenetic origins and evolved using iterated mutagenesis and screening to enhance activity, resulting in second-generation motifs that on average outperform tevopreQ_1_ across delivery modalities and cell types^27^. We delivered 2 mg kg^−1^ s1 PE-LNPs (1:0.9:0.1 PE6c mRNA:epegRNA:ngRNA by total RNA mass) with either tevopreQ_1_ epegRNA or epegRNA with one of four novel 3′ motifs to adult C57BL/6 mice via RO injection. All epegRNAs were programmed to install the same +1 TTAC insertion at *Pcsk9*. We observed that using epegRNA containing the 3′ motif eSBRMV1-A improved average prime editing in the bulk liver over tevopreQ_1_ from 17% to 26% (Fig. 2d, *P* = 0.0006). We selected the eSBRMV1-A motif for subsequent PE-LNP optimization and evaluation of the *Pcsk9* +1 TTAC insertion. We refer to the optimized editor and 3′ epegRNA motif PE-LNPs as s2 PE-LNPs.

**Extended Data Fig. 1:**
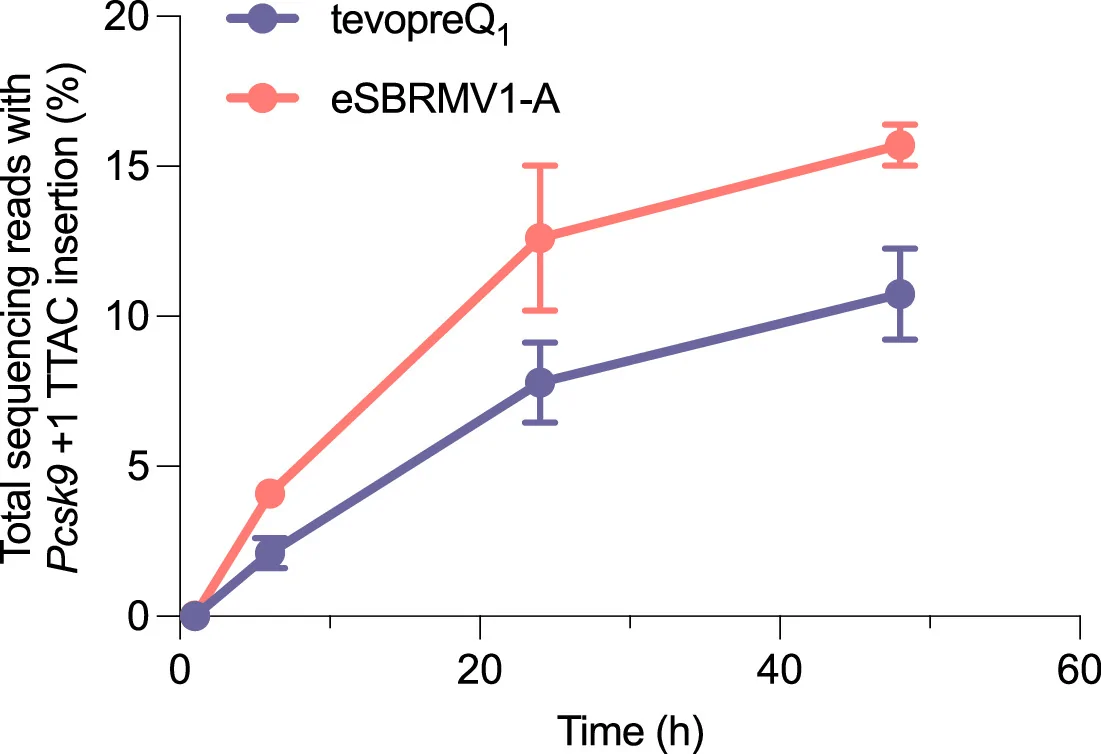
Prime editing at early timepoints in the bulk liver of mice treated with PE-LNPs. Prime editing efficiencies in the bulk liver of 6-week-old female C57BL/6 mice dosed via RO injection with s1 PE-LNPs encapsulating epegRNA with either a 3′ tevopreQ1 motif or eSBRMV1-A motif. PE-LNPs were delivered at 2 mg/kg total RNA dose (1:0.9:0.1, mRNA:epegRNA:ngRNA). Both epegRNAs were designed to install the *Pcsk9* + 1 TTAC insertion. Tissue from livers was harvested at 1, 6, 24, and 48 h post-injection and analyzed by HTS. Each data point represents the mean of *n* = 3 mice and error bars represent s.e.m. Source Data

To assess whether improved editing efficiency at *Pcsk9* using synthetic epegRNA containing the eSBRMV1-A motif was due to enhanced stability of this epegRNA over epegRNAs containing the tevopreQ_1_ motif, we delivered 2 mg kg^−1^ PE-LNPs (1:0.9:0.1 PE6c mRNA:epegRNA:ngRNA by RNA mass) with either tevopreQ_1_ epegRNA or eSBRMV1-A epegRNA encoding the *Pcsk9* +1 TTAC insertion to adult C57BL/6 mice via RO injection. We collected livers at 1, 6, 24 and 48 h post-injection to measure epegRNA levels in liver lysate by reverse-transcription quantitative polymerase chain reaction (RT–qPCR) and prime editing levels by high-throughput sequencing (HTS) (Fig. 2e). Normalized to each epegRNA’s abundance 1 h after dosing, we observed a modestly larger fraction of eSBRMV1-A epegRNA in liver lysate at 24 h post-injection compared with tevopreQ_1_ (Fig. 2f, *P* = 0.0883, two-way analysis of variance (ANOVA) with Bonferroni’s multiple comparisons test), suggesting that the eSBRMV1-A motif enhances stability of the epegRNA following LNP-mediated delivery to the mouse liver. By HTS analysis of genomic DNA, we observed significantly higher levels of prime editing in the bulk liver with eSBRMV1-A epegRNA when compared with tevopreQ_1_ at the 24-h (13% versus 7.8% editing, *P* = 0.0004) and 48-h (16% versus 11% editing, *P* = 0.0003) timepoints (Extended Data Fig. 1).

**Extended Data Fig. 2:**
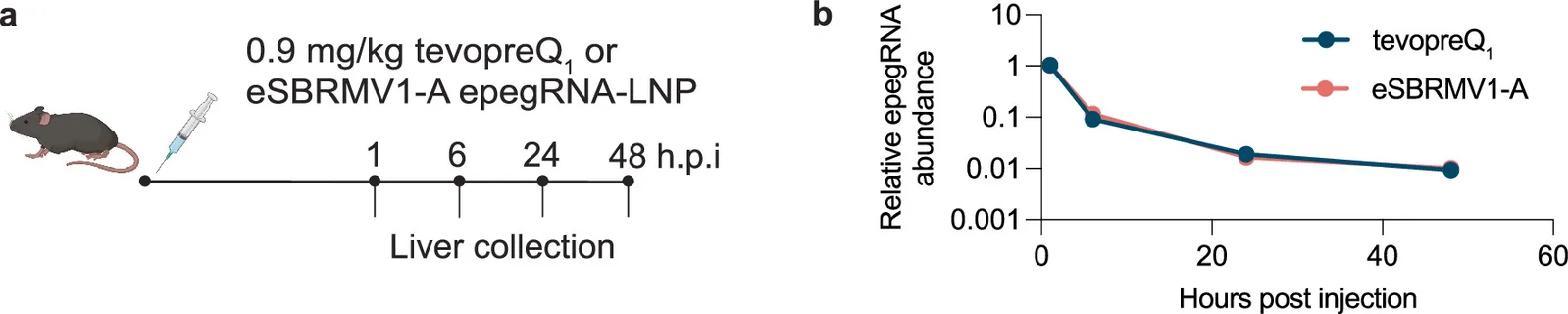
epegRNA abundance in the liver following LNP delivery without PE mRNA. **a**, Schematic of dosing and liver collection at 1, 6, 24, and 48 hours post injection (h.p.i.) for analysis of epegRNA abundance in the liver. **b**, Relative abundance of epegRNA with either tevopreQ_1_ or eSBRMV1-A 3′ motifs in bulk liver lysate following 0.9 mg/kg epegRNA-LNP injection. epegRNA abundance at different time points following injection was measured by RT-qPCR and normalized to respective epegRNA abundance at 1 h. Each data point represents mean of *n* = 3 mice and error bars represent s.e.m. Illustrations in **a** created in BioRender; Jiang, A. https://biorender.com/tha3ui7 (2026). Source Data

**Extended Data Fig. 3:**
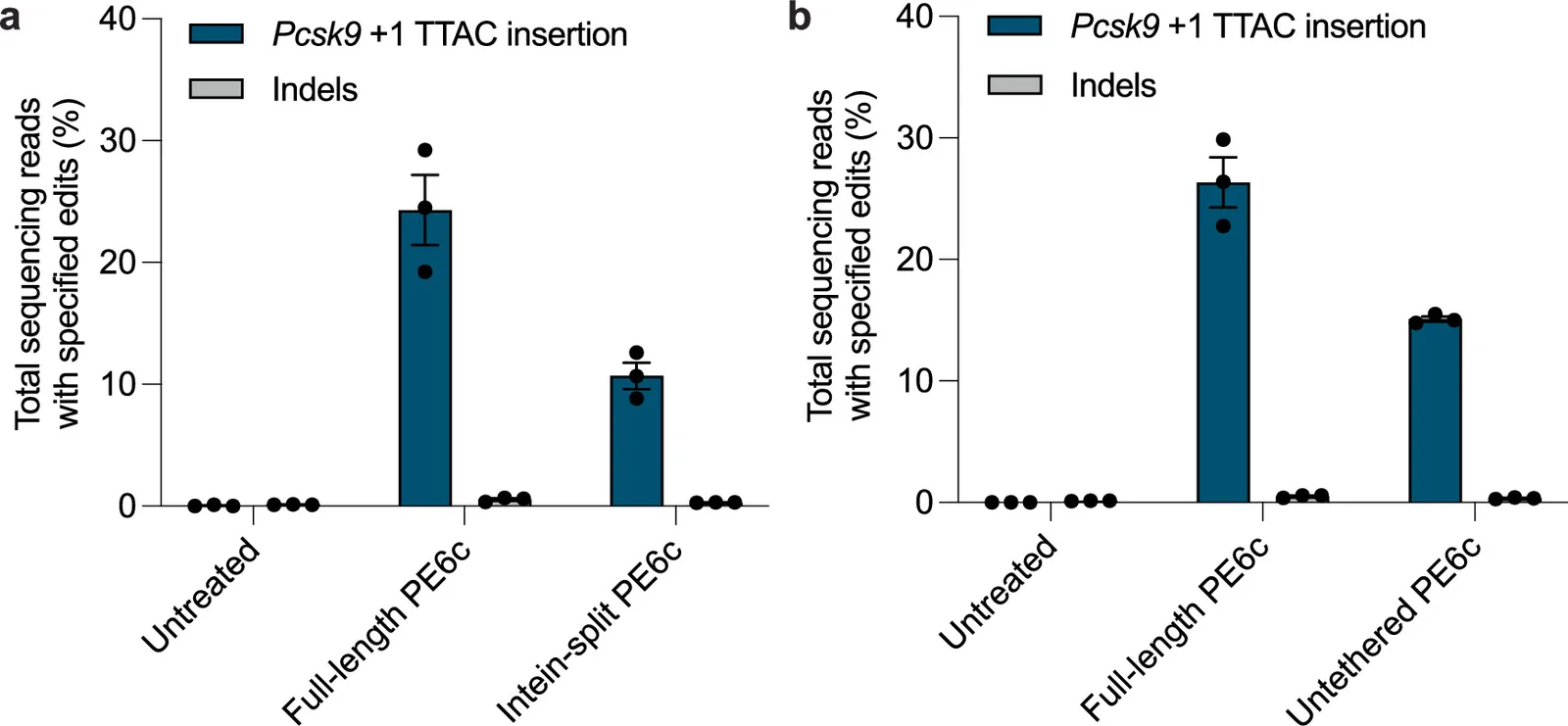
Evaluation of intein-split and untethered PE-LNPs in mice. **a**, Prime editing efficiencies in the bulk liver of 6-week-old female C57BL/6 mice dosed by RO injection at 2 mg/kg PE-LNPs (1:0.9:0.1 mRNA:epegRNA:ngRNA by total RNA mass). PE6c was delivered either as a full-length mRNA (full-length PE6c) or intein-split mRNAs (intein-split PE6c). Full-length PE6c mRNA was formulated in a single LNP whereas the intein-split PE6c mRNAs were formulated separately and admixed at a 1:1 mass ratio for each half. **b**, Prime editing efficiencies in the bulk liver of 6-week-old female C57BL/6 mice dosed by RO injection at 2 mg/kg PE-LNPs (1:0.9:0.1 mRNA:epegRNA:ngRNA by total RNA mass). PE6c was delivered either as full-length mRNA formulated in a single LNP (full-length PE6c) or as the SpCas9 nickase and eeTf1 mRNAs formulated in separate LNPs and admixed at a 1:1 mass ratio for each mRNA (untethered PE6c). For a and b, all PE-LNPs installed the *Pcsk9* + 1 TTAC insertion edit. Liver tissue was harvested one week after injection and analyzed by HTS. Data points represent individual mice, and error bars represent mean ± s.e.m. of *n* = 3 mice. Source Data

To determine whether the observed pegRNA stabilization was an intrinsic property of the eSBRMV1-A motif or required the presence of the PE, we performed a similar experiment but only delivered 0.9 mg kg^−1^ LNP encapsulating either tevopreQ_1_ epegRNA or eSBRMV1-A epegRNA (without PE6c mRNA) to C57BL/6 mice via RO injection and quantified epegRNA abundance in liver lysate by RT–qPCR (Extended Data Fig. 2a). We observed that epegRNA abundance, when normalized to each epegRNA’s abundance at the 1-h timepoint, was similar between both motifs at the 6-, 24- and 48-h timepoints (Extended Data Fig. 2b). These data suggest that the enhanced stabilizing effect of the eSBRMV1-A motif depends on the presence of the PE within the cell. We speculate that PE complexation with epegRNA may impede 5′ exonuclease degradation of the epegRNA, which could otherwise dominate epegRNA degradation kinetics in the absence of PE and thus mask the 3′ end-stabilizing benefit of the eSBRMV1-A motif. In addition to providing greater stability to the epegRNA when bound to PE, the eSBRMV1-A motif may further enhance prime editing compared with tevopreQ_1_ by promoting more efficient ribonucleoprotein assembly or favouring conformational states that facilitate DNA flap binding to the primer binding site or reverse transcription of the epegRNA template^35,36^.

Together, these findings indicate that newly developed 3′ epegRNA motifs such as eSBRMV1-A can enhance LNP-delivered in vivo prime editing efficiencies by impeding pegRNA degradation or enhancing ribonucleoprotein formation while complexed with the PE protein.

We hypothesized that the transience of PE and synthetic gRNAs in vivo may amplify any stoichiometric mismatch among the prime editing components, and that optimizing the stoichiometry of the PE mRNA, epegRNA and ngRNA may therefore improve editing efficiency. We dosed 2 mg kg^−1^ s2 PE-LNPs to install the *Pcsk9* +1 TTAC insertion at varying ratios of PE mRNA-LNP to epegRNA- and ngRNA-LNP via RO injection. We evaluated varying RNA mass ratios of mRNA-LNP to gRNA-LNP (2:1, 1:1, 1:2, 1:3 and 1:4 mRNA-LNP:gRNA-LNP by RNA mass), in which the gRNA fraction represents the combined mass of epegRNA and ngRNA maintained at a constant ratio of 9:1 (RNA wt/wt) epegRNA-LNP to ngRNA-LNP. We observed similar, plateauing levels of prime editing in the bulk liver of mice treated with 1:1 (21% editing), 1:2 (21% editing) and 1:3 (22% editing) ratios of mRNA-LNP to gRNA-LNP (Fig. 2g). Ratios skewed even more towards mRNA or gRNA, corresponding to 2:1 (18% editing) or 1:4 (20% editing) mRNA-LNP to gRNA-LNP, respectively, resulted in slightly lower average prime editing levels in the bulk liver, but these differences were not statistically significant (*P* ≥ 0.337 for all pairwise comparisons of treated groups). These data suggest that the stoichiometry of epegRNA and ngRNA compared with PE mRNA within the range tested (2:1 to 1:4 mRNA:gRNA) was not a strong determinant of in vivo PE efficiency in the liver. We selected a ratio of 1:2 mRNA-LNP to gRNA-LNP for subsequent PE-LNP optimization and evaluation for the *Pcsk9* +1 TTAC insertion and refer to this PE-LNP system that uses optimized PE, 3′ epegRNA motif and RNA ratio as s3 PE-LNP.

Reasoning that optimized mRNA manufacturing would improve translation of PE and therefore increase prime editing efficiency, we next evaluated whether PE mRNA produced by a commercial vendor (GenScript) using optimized untranslated regions (UTRs) and purification methods could improve in vivo prime editing of s3 PE-LNPs over mRNA produced in-house. mRNA purchased from GenScript contains the same coding sequence as our mRNA transcribed in-house but incorporates proprietary 5′ and 3′ UTRs and is purified by high-performance liquid chromatography (HPLC) to remove double-stranded DNA (dsRNA) carryover from in vitro transcription (IVT), a process shown to enhance protein production in vivo^37^. We delivered 2 mg kg^−1^ s3 PE-LNPs with either in-house PE6c mRNA or GenScript PE6c mRNA to adult C57BL/6 mice via RO injection and evaluated prime editing of the *Pcsk9* +1 TTAC insertion after one week. s3 PE-LNPs containing GenScript PE6c mRNA resulted in 49% average prime editing in the bulk liver, a 1.9-fold increase over s3 PE-LNPs containing in-house PE6c mRNA (26% average editing, Fig. 2h, *P* = 0.0016, unpaired *t*-test). Unless otherwise noted, we used GenScript HPLC-purified mRNA for all subsequent in vivo experiments.

Systematic optimization of the mRNA-encoded PE, the 3′ motif of the epegRNA, and the ratio of the three RNA-encapsulating PE-LNP components thus yielded 63-fold and 13-fold overall improvement in in vivo prime editing efficiency in the mouse liver mediated by LNP delivery compared with s0.1 and s0.2 PE-LNPs, respectively (Figs. 1c and 2h). However, the choice of PE, 3′ epegRNA motif, and PE-LNP ratio for a given application may vary. Therefore, we recommend following a systematic workflow to identify the optimal PE (s1 PE-LNP), 3′ epegRNA motif (s2 PE-LNP) and PE-LNP stoichiometry (s3 PE-LNP) for a given application (Fig. 2i). When administering the optimized s3 PE-LNP in vivo, we recommend using mRNA that has been rigorously purified from contaminating dsRNA.

## PE-LNPs exhibit distinct physicochemical properties based on RNA cargo

**Fig. 3:**
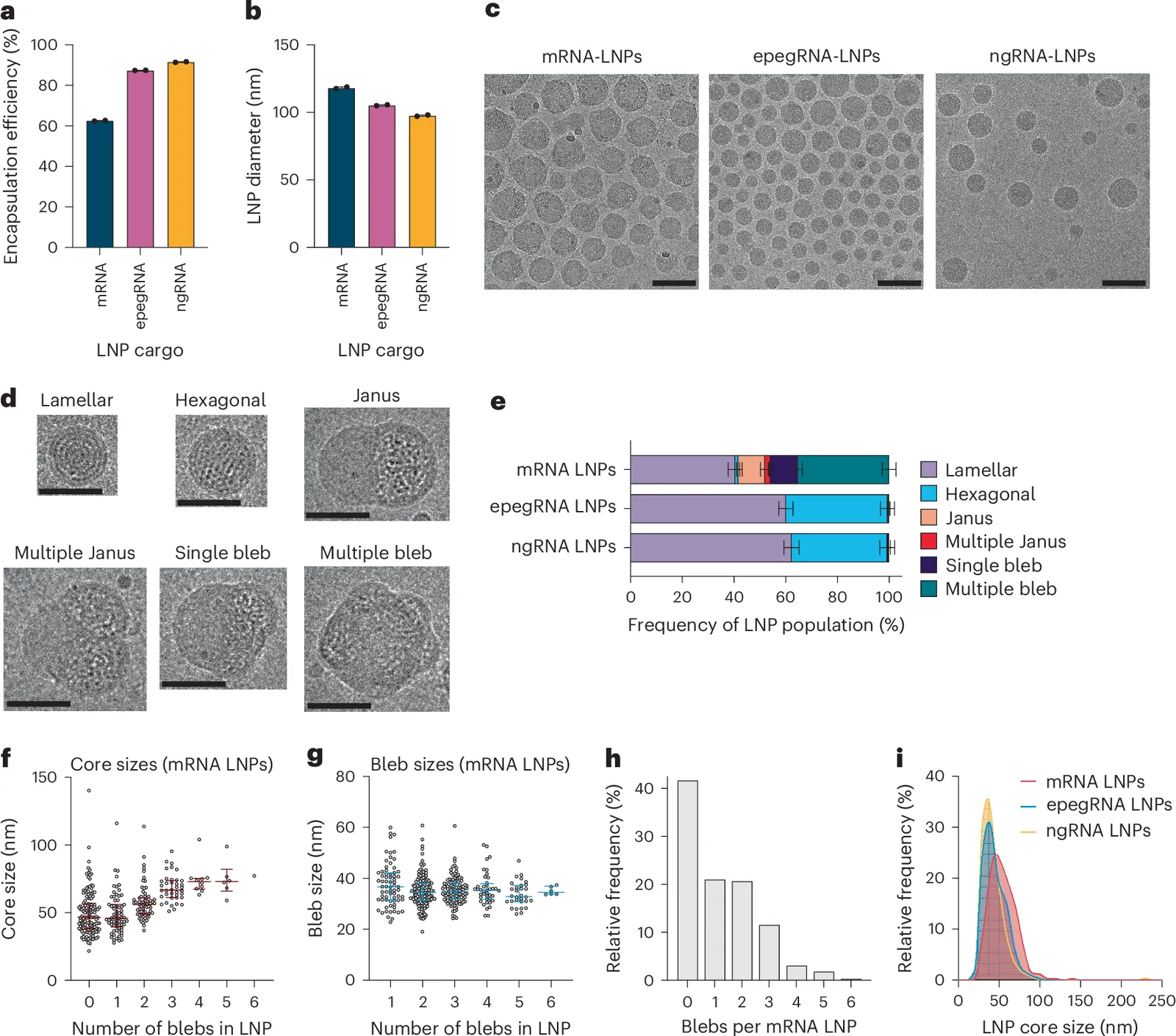
Physicochemical characterization of s3 PE-LNPs. **a**,**b**, RNA EE (**a**) and diameter (**b**) of LNPs packaging PE mRNA, epegRNA or ngRNA. Data points represent technical replicates of *n* = 2 repeated measurements, and bars represent the mean. **c**, Representative cryo-EM images of s3 PE-LNPs. Scale bars, 100 nm. **d**, Representative cryo-EM images of LNP morphologies. Imaging was performed once on a single LNP formulation batch. Scale bars, 50 nm. **e**, Classification and quantification of the types of LNP morphology observed in the cryo-EM images of mRNA, epegRNA and ngRNA LNPs. Error bars represent mean ± s.e.m. **f**,**g**, Core (*n* = 137, 69, 68, 38, 10, 6 and 1 LNPs for 0 to 6 blebs, respectively) (**f**) and bleb (*n* = 69, 136, 114, 40, 30 and 6 LNPs for 1 to 6 blebs, respectively) (**g**) sizes observed in the cryo-EM images of PE mRNA LNPs. Error bars represent mean ± s.e.m. **h**, Quantification of the number of blebs per individual mRNA LNPs observed in the cryo-EM images. *n* = 329 LNPs analysed. **i**, Quantification of core size for mRNA, epegRNA and ngRNA LNPs observed in the cryo-EM images. For **e** and **i**, *n* = 329, 321 and 276 individual LNPs for mRNA, epegRNA and ngRNA, respectively. Source data

**Extended Data Fig. 4:**
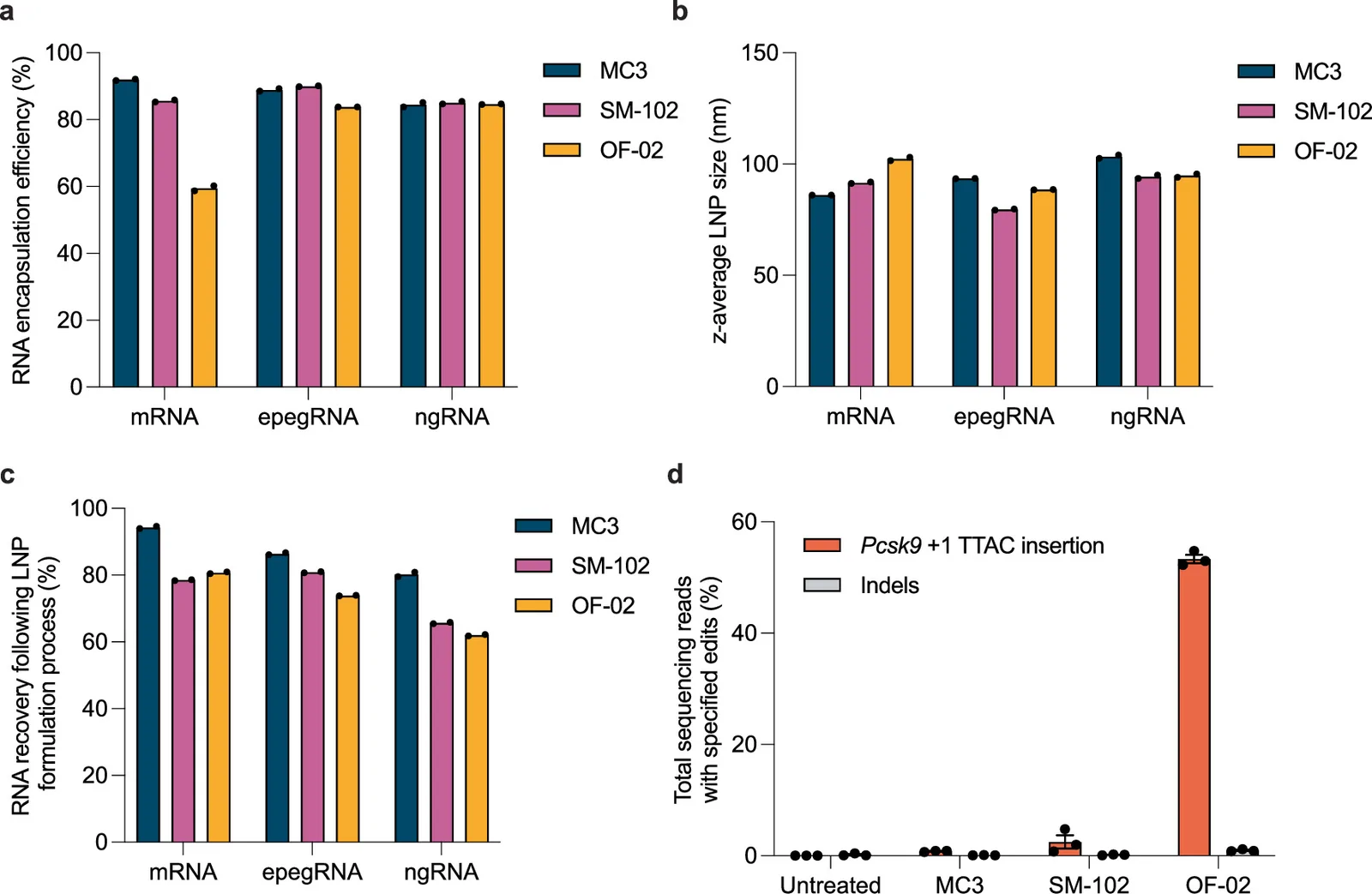
Physicochemical characterization of and in vivo prime editing with clinical LNP formulations. **a**, RNA encapsulation efficiency and **b**, z-average size of MC3, SM-102, and OF-02 LNPs formulated to encapsulate PE6c mRNA, epegRNA, or ngRNA. **c**, RNA mass recovery (%) relative to the initial aqueous RNA input following MC3, SM-102, or OF-02 LNP formulation. For **a**, **b**, and **c**, data points represent technical replicates of *n* = 2 repeated measurements and bars represent the mean. **d**, Prime editing efficiencies in the bulk liver of 6-week-old female C57BL/6 mice dosed via RO injection with 1.5 mg/kg of MC3-, SM-102-, or OF-02 based s3 PE-LNPs. Tissue from livers was harvested 7 days after the injection and analyzed by HTS. Data points represent individual mice, and error bars represent mean ± s.e.m. of n = 3 mice. Source Data

With an optimization workflow in hand, we characterized the physicochemical properties of s3 mRNA-, epegRNA- and ngRNA-LNPs to better understand the nanoscale features and structural complexity of the optimized multicomponent system. Delivery properties of RNA-encapsulating LNPs including biodistribution and transfection efficiency are probably influenced by their size, morphology and encapsulation efficiency (EE)^20,38^. We measured the EE of different RNA cargoes into OF-02 LNPs using an adapted Ribogreen assay and observed that the EE for mRNA-LNPs (63%) was lower than those of the two gRNA-LNP formulations (87% EE for epegRNA-LNPs and 92% EE for ngRNA-LNPs), suggesting that the substantially larger size of the PE mRNA cargo may limit EE (Fig. 3a). Based on these encapsulation efficiencies, the optimal 1:1.8:0.2 (mRNA:epegRNA:ngRNA) s3 PE-LNP ratio of total RNA dosed by mass corresponds to an encapsulated RNA mass ratio of 1:2.5:0.3 (mRNA:epegRNA:ngRNA). We then measured the hydrodynamic size of the three LNPs using dynamic light scattering (DLS) and observed that the mRNA-LNPs were larger in *z*-average size (118 nm) than both gRNA-LNP formulations, which were comparable in size (105 nm for epegRNA-LNPs and 98 nm for ngRNA-LNPs) (Fig. 3b). Based on these physicochemical results, we next investigated whether the performance of our PE-LNPs could be further improved by using clinically validated ionizable lipids such as MC3 or SM-102^39,40^. Despite exhibiting more favourable RNA EE and yields, neither formulation outperformed OF-02 for in vivo liver prime editing, so we continued to use OF-02 for all subsequent experiments (Supplementary Note 2 and Extended Data Fig. 4).

We next performed cryo-electron microscopy (cryo-EM) on OF-02-based s3 mRNA-, epegRNA- and ngRNA-LNPs (Fig. 3c). Cryo-EM enabled us to image the core structures of our LNPs, with the PEG shell structures remaining largely unresolved. Analysis of the cryo-EM images revealed six LNP morphologies: lamellar, hexagonal, Janus, multiple-Janus, single-bleb and multiple-bleb cores (Fig. 3d). Fast Fourier transform patterns of the cryo-EM images validated the distinct lamellar and hexagonal core morphologies (Supplementary Fig. 1). LNPs encapsulating small gRNAs (epegRNA- and ngRNA-LNPs) showed lamellar and hexagonal core morphologies at a similar number distribution (Fig. 3e). By contrast, mRNA-LNPs showed a broader mixture, consisting of ~40% lamellar cores, ~40% cores with multiple blebs, ~10% cores with a single bleb and ~10% Janus cores (Fig. 3e). These observations suggest that bleb formation is a prominent feature of PE mRNA-LNPs.

Prior studies have shown that blebs form when LNPs encapsulate large mRNAs and that a greater number of blebs per LNP is correlated with increased potency, including for in vivo mRNA delivery^41^. We reasoned that the formation of blebs is thermodynamically favoured during LNP self-assembly, potentially due to the increased entropy associated with mRNA flexibility and favourable lipid–lipid interactions upon phase segregation of the mRNAs into blebs. This hypothesis is supported by our observation that LNPs encapsulating small gRNAs showed no blebs (Fig. 3c,e). We further reasoned that each bleb probably contains a single PE mRNA molecule, which is supported by two observations. First, while the overall LNP core size increased with the number of blebs (Fig. 3f), the size of the individual blebs remained consistent regardless of the number of blebs (Fig. 3g). Second, the distribution of the number of blebs per mRNA-LNP was consistent with the experimentally determined distributions of the number of mRNAs encapsulated per LNP of similar formulations^34^ (Fig. 3h), which revealed a majority of empty LNPs (no blebs) and a subset of LNPs containing several mRNA copies (multiple blebs).

Lastly, we quantified the size of the LNPs by cryo-EM (Fig. 3i). Cryo-EM showed that the cores of ngRNA-LNPs were smaller than those of epegRNA-LNPs, which were smaller than those of mRNA-LNPs. This trend is consistent with the DLS characterization data and suggests that the size differences between our PE-LNPs result from differences in core sizes rather than PEG shell thicknesses.

Our results suggest that the optimal formulation for LNPs encapsulating large mRNA and LNPs encapsulating small RNAs may differ. For example, the optimal formulation for mRNA-LNPs should maximize mRNA loading, which is consistent with bleb number, and self-assemble in a different local thermodynamic context than epegRNA- or ngRNA-LNPs. Although we used a single formulation in this study to encapsulate each RNA species individually, followed by admixing the LNPs before injection, the future development of LNP formulations tailored specifically for intracellular delivery of large PE mRNA cargoes may further improve prime editing efficiencies in vitro and in vivo.

## Characterization of PE-LNP pharmacodynamics

**Fig. 4:**
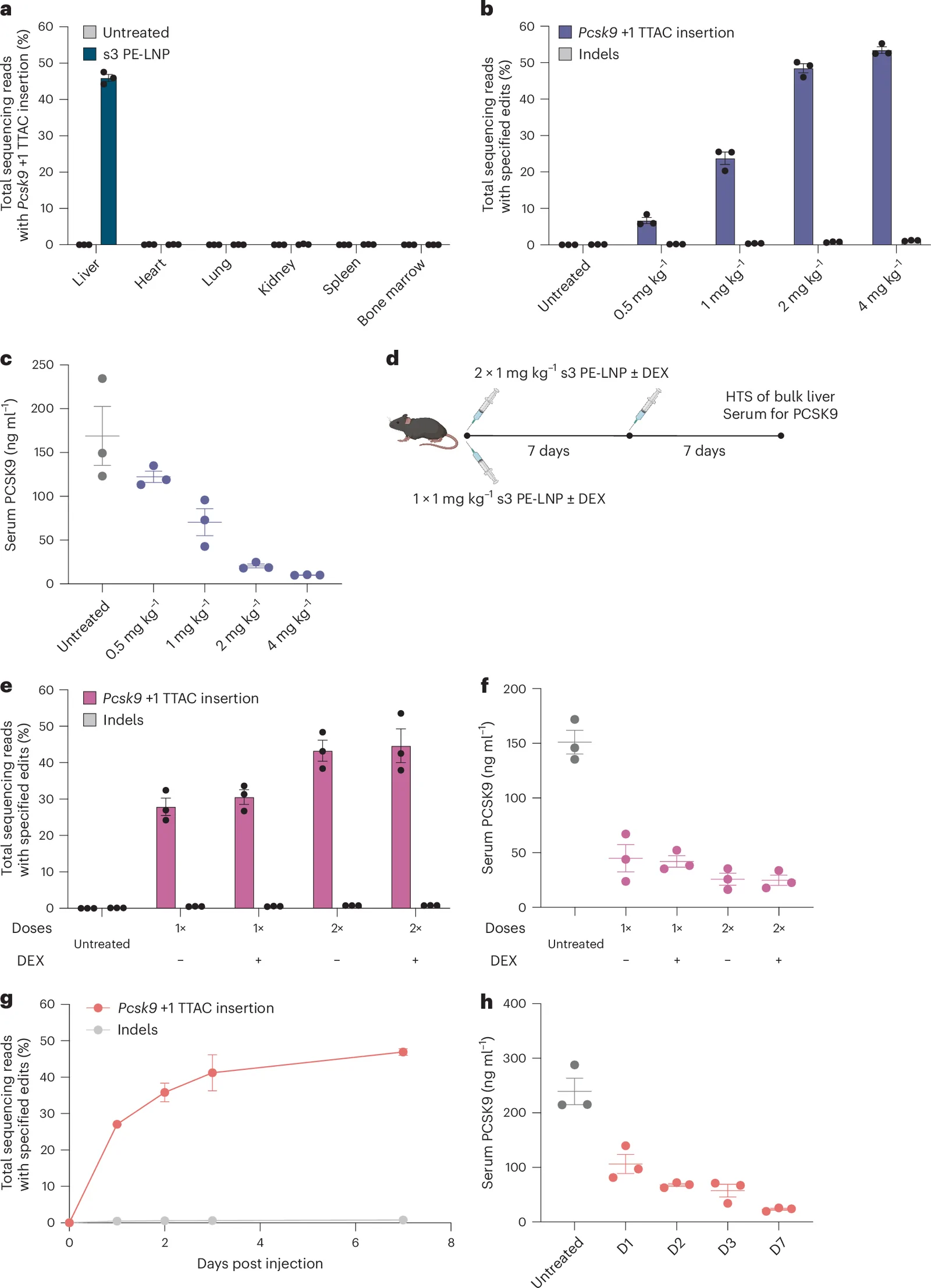
Characterization of PE-LNP pharmacodynamics. **a**, Biodistribution of prime editing across tissues collected from 6-week-old female C57BL/6 mice receiving 2 mg kg^−1^ s3 PE-LNP via RO injection. Tissues were collected 7 days after the injection and analysed by HTS. **b**, Prime editing efficiencies in the bulk liver of 6-week-old female C57BL/6 mice dosed via RO injection with varied doses (0.5 mg kg^−1^ up to 4 mg kg^−1^) of s3 PE-LNPs. Tissue from livers was collected 7 days after the injection and analysed by HTS. **c**, Concentration of PCSK9 protein in serum collected from mice treated in **b**. **d**, Schematic of PE-LNP repeat dosing with and without administration of DEX as well as liver and serum collection. **e**, Prime editing efficiencies in the bulk liver of 6-week-old female C57BL/6 mice treated with either one or two doses (1 mg kg^−1^ per dose, with dosing spaced out by 7 days) of s3 PE-LNPs via RO injection, with or without pretreatment with DEX (5 mg kg^−1^, intraperitoneally). For groups receiving DEX, the steroid was administered 30 min before each PE-LNP injection. Tissue from livers was collected 7 days after the final injection and analysed by HTS. **f**, Concentration of PCSK9 protein in serum collected from mice treated in **e**. **g**, Prime editing efficiencies in the bulk liver of 6-week-old female C57BL/6 mice receiving 2 mg kg^−1^ s3 PE-LNPs via RO injection. Tissue from livers was collected at varying timepoints (1, 2, 3 and 7 days after injection) and analysed by HTS. **h**, Concentration of PCSK9 protein in serum collected from mice treated in **g**. For **a**–**c**, **e**, **f** and **h**, data points represent individual mice and error bars represent mean ± s.e.m. of *n* = 3 mice. For **g**, data points represent the mean of *n* = 3 mice and error bars represent s.e.m. Illustrations in **d** created in BioRender; Jiang, A. https://biorender.com/4p640s3 (2026). Source data

**Extended Data Fig. 5:**
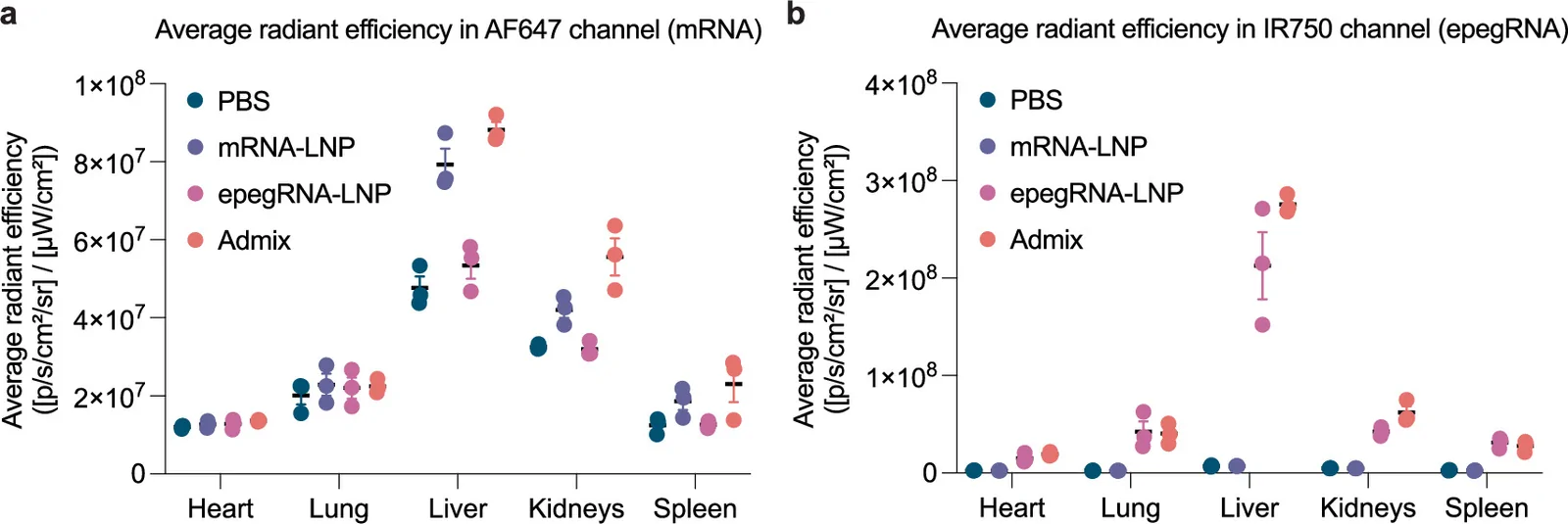
Biodistribution of single and admixed LNP formulations. Average radiant efficiency in **a**, AF647 channel (AF647-conjugated PE6c mRNA) and **b**, IR750 (IR750-conjugated epegRNA) channel for major organs 1 h after RO injection of PBS, 0.66 mg/kg mRNA-LNP, 1.2 mg/kg epegRNA-LNP, or 1.88 mg/kg of admixed LNPs (1:1.8 ratio of mRNA-LNP to epegRNA-LNP by total RNA concentration). Data points represent individual mice, and error bars represent mean ± s.e.m. of *n* = 3 mice. Female 12-week-old C57BL/6 mice were used for all groups. Source Data

**Extended Data Fig. 6:**
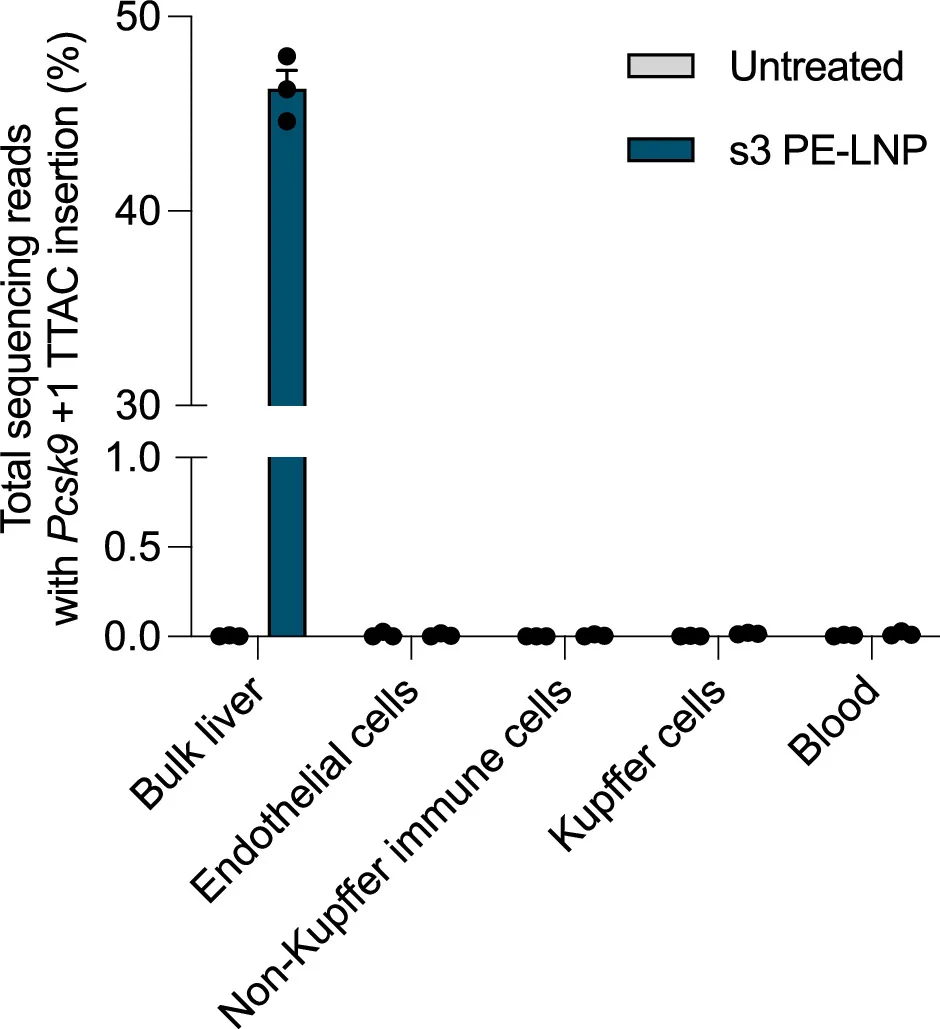
Prime editing in different liver cell populations and whole blood. Prime editing efficiencies in the bulk liver of 6-week-old female C57BL/6 mice dosed via RO injection with 2 mg/kg of s3 PE-LNPs. 1 week after injection, liver tissue and blood was harvested, single-cell suspensions generated from liver tissue were stained and sorted using FACS, and gDNA was harvested from sorted cells and blood for analysis by HTS. Data points represent individual mice, and error bars represent mean ± s.e.m. of *n* = 3 mice. Source Data

Having developed a PE-LNP strategy capable of efficient prime editing in the liver of mice, we next evaluated the pharmacodynamics of the system. To determine the extent of *Pcsk9* prime editing in the liver and other tissues of mice, we dosed 2 mg kg^−1^ s3 PE-LNPs into adult C57BL/6 mice via RO injection and performed targeted amplicon sequencing of genomic DNA extracted from six different organs (liver, heart, lung, kidney, spleen and bone marrow) 1 week after injection. Consistent with the reported biodistribution of functional mRNA delivery following systemic injection of OF-02 LNPs^28^, we observed that prime editing occurred predominantly in the liver, with 49% *Pcsk9* +1 TTAC insertion in the bulk liver and no editing observed in non-hepatic tissues (Fig. 4a). As a physical assessment of LNP distribution, we performed ex vivo imaging of organs to compare the localization of fluorescently labelled PE6c mRNA and epegRNA separately encapsulated within LNPs 1 h after injection (Extended Data Fig. 5). We found that the admixing process did not reduce the tissue associated signal for either RNA species when compared with single RNA-LNP formulations, further supporting the use of the admixed system for systemic delivery. To evaluate editing in the blood and liver non-parenchymal cells, we collected whole blood and bulk livers 1 week after s3 PE-LNP injections, generated single-cell suspensions from liver tissue and stained for endothelial cells (CD31^+^CD45^−^), non-Kupffer immune cells (CD45.2^+^F4/80^−^) and Kupffer cells (CD45.2^+^F4/80^+^) (Supplementary Fig. 2). Cells were sorted by fluorescence-activated cell sorting (FACS) after which genomic DNA was purified and analysed by HTS. We did not observe *Pcsk9* editing in any non-parenchymal cell population or in whole blood, suggesting that OF-02 mediated prime editing is largely restricted to hepatocytes in the liver (Extended Data Fig. 6).

To determine the dose response of PE-LNP, we administered s3 PE-LNPs at doses ranging from 0.5 mg kg^−1^ to 4 mg kg^−1^ to adult C57BL/6 mice via RO injection. We observed dose-dependent levels of *Pcsk9* editing in the bulk liver, reaching 53% at the 4 mg kg^−1^ saturating dose (Fig. 4b). In addition, we analysed serum collected from the same mice to quantify the levels of circulating PCSK9 protein. The +1 TTAC insertion edit at the end of *Pcsk9* exon 1 introduces both a reading frame shift and premature termination codon which should result in a reduction of PCSK9 protein levels in circulation^22^. Serum PCSK9 in PE-LNP-treated mice decreased in a dose-dependent manner compared with age-matched, untreated control mice, with up to 94% reduction in serum PCSK9 concentration observed in the 4 mg kg^−1^ treatment group (Fig. 4c). Reduction in serum PCSK9 was consistent with editing levels in the bulk liver across the range of doses evaluated.

In contrast to viral delivery systems, LNPs can be dosed repeatedly, which has been demonstrated to improve in vivo genome editing compared to a single administration^15,42^. We therefore evaluated whether repeat dosing of PE-LNPs could improve in vivo prime editing. Given the modest editing level observed in the liver following a single 1 mg kg^−1^ injection of s3 PE-LNPs (24% editing; Fig. 4b), we administered either one or two 1 mg kg^−1^ doses of s3 PE-LNPs to C57BL/6 mice via RO injection (Fig. 4d). For mice receiving two doses, injections were spaced 7 days apart. We also tested whether pretreatment with 5 mg kg^−1^ dexamethasone (DEX) injected intraperitoneally 30 min before RO administration of PE-LNPs would improve editing. DEX is a corticosteroid administered in the clinic before injection of LNP therapies and has been demonstrated to improve LNP-mediated Cas9 nuclease editing in mice^43^. One week following the second injection, genomic DNA was collected from bulk livers and analysed by HTS for *Pcsk9* +1 TTAC insertion. For mice receiving a single dose of PE-LNPs, we observed similar levels of editing without (28%) or with DEX (31%) pretreatment (Fig. 4e). The second administration of PE-LNPs resulted in higher levels of total editing (43% without DEX pretreatment and 45% with DEX pretreatment) compared with single administration. Serum PCSK9 levels in PE-LNP-treated mice mirrored the trends observed in *Pcsk9* editing, with a single dose reducing PCSK9 protein levels by 70% (without DEX) and 72% (with DEX), and the second dose further reducing PCSK9 levels by 83% without DEX and 84% with DEX (Fig. 4f). These results demonstrate the ability to readminister PE-LNPs to improve editing efficiency in the liver of mice and indicate that DEX pretreatment is not necessary but may be beneficial for enhanced editing outcomes in mice.

**Extended Data Fig. 7:**
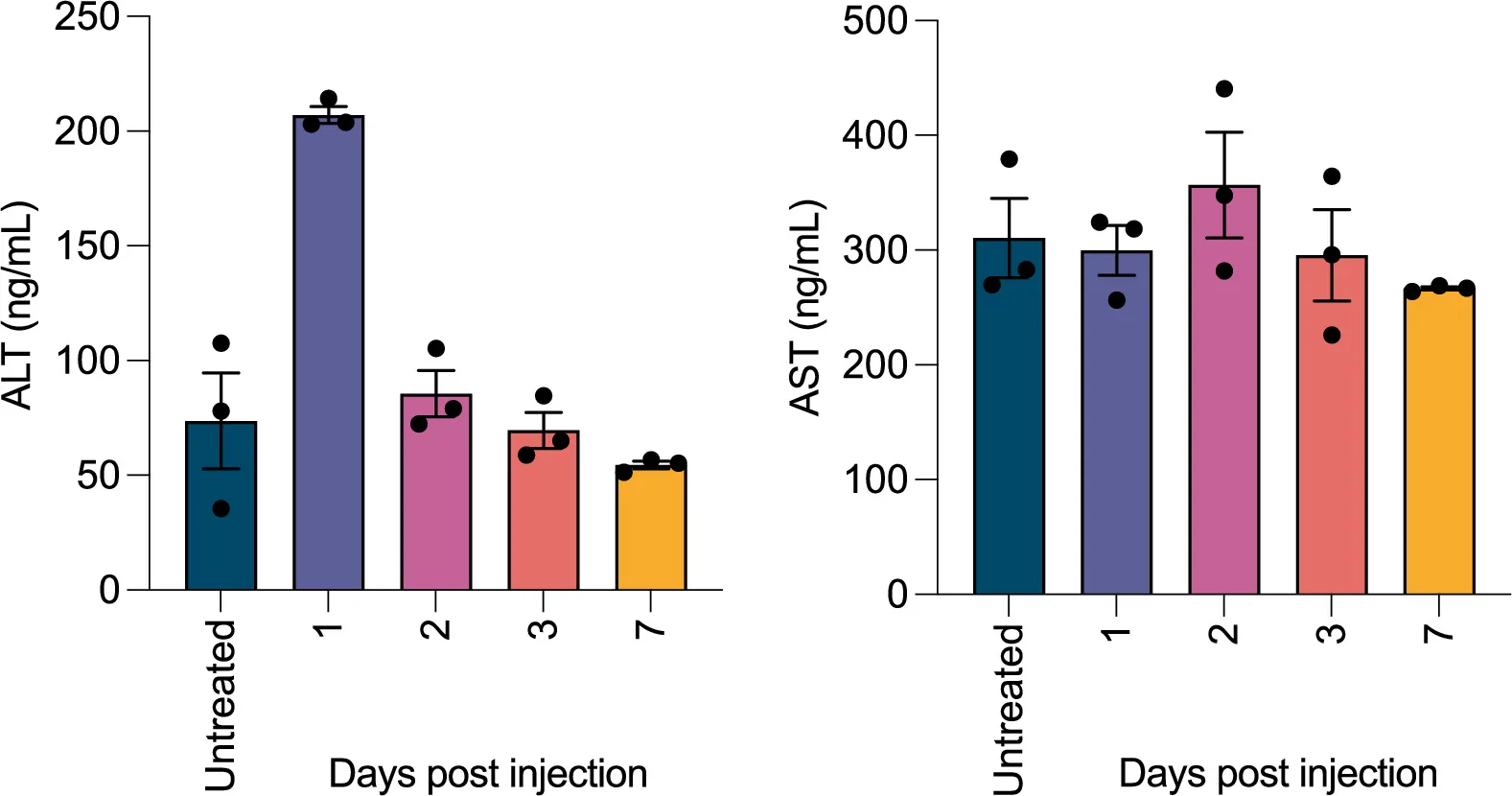
ALT and AST levels in serum following PE-LNP delivery to C57BL/6 mice. Concentrations of alanine aminotransferase (ALT, left) and aspartate transaminase (AST, right) in serum of C57BL/6 mice treated with 2 mg/kg s3 PE-LNPs to install the *Pcsk9* + 1 TTAC insertion via RO injection. Data points represent individual mice, and error bars represent mean ± s.e.m. of *n* = 3 mice. Source Data

An additional, potential advantage of LNP-mediated prime editing is the faster production of the PE protein from nucleoside-modified mRNA^44^, enabling a more rapid onset of therapeutic editing and phenotypic change compared with AAV-mediated delivery. To assess the kinetics of LNP-mediated prime editing, we dosed 2 mg kg^−1^ s3 PE-LNPs via RO injection and performed targeted amplicon sequencing of genomic DNA extracted from the livers of treated mice 1, 2, 3 and 7 days after injection. We observed substantial prime editing in the bulk liver after 1 day (27%), 2 days (36%) and 3 days (41%), with editing after 7 days reaching 47% (Fig. 4g). This result is consistent with rapid editing observed for Cas9 nuclease and base editing when delivered by LNP^42,45^. We analysed serum at the different timepoints and observed similarly rapid reduction in serum PCSK9 levels compared with untreated control mice after 1 day (56%), 2 days (71%) and 3 days (76%), with PCSK9 reduction reaching 90% after 7 days (Fig. 4h). We also analysed serum for alanine aminotransferase (ALT) and aspartate transaminase (AST) levels to assess PE-LNP toxicity. Consistent with previous studies^12,15,17,46^, we observed mild, transient elevations in serum ALT 1 day after 2 mg kg^−1^ LNP injection, which returned to levels similar to those of untreated controls by 3 days post-injection (Extended Data Fig. 7). Taken together, these results demonstrate that LNP-mediated delivery of RNA-based prime editing components enables safe, efficient and rapid editing in the liver of mice.

## Correction of PKU in diseased mice using PE-LNPs

With an effective workflow to optimize prime editing strategies for LNP delivery, we next tested whether PE-LNPs could correct pathogenic mutations in the liver to treat metabolic disorders in mice. In principle, base editing strategies such as the one used to treat K. J. Muldoon^15^ can correct inborn errors of metabolism caused by transition point mutations. Prime editing, however, expands the range of targetable mutations to include virtually any local DNA change, increasing the applicability to patients with inborn errors of metabolism who could benefit from personalized genome editing treatments. In addition, prime editing avoids the possibility of undesired bystander editing or transcriptome editing.

**Fig. 5:**
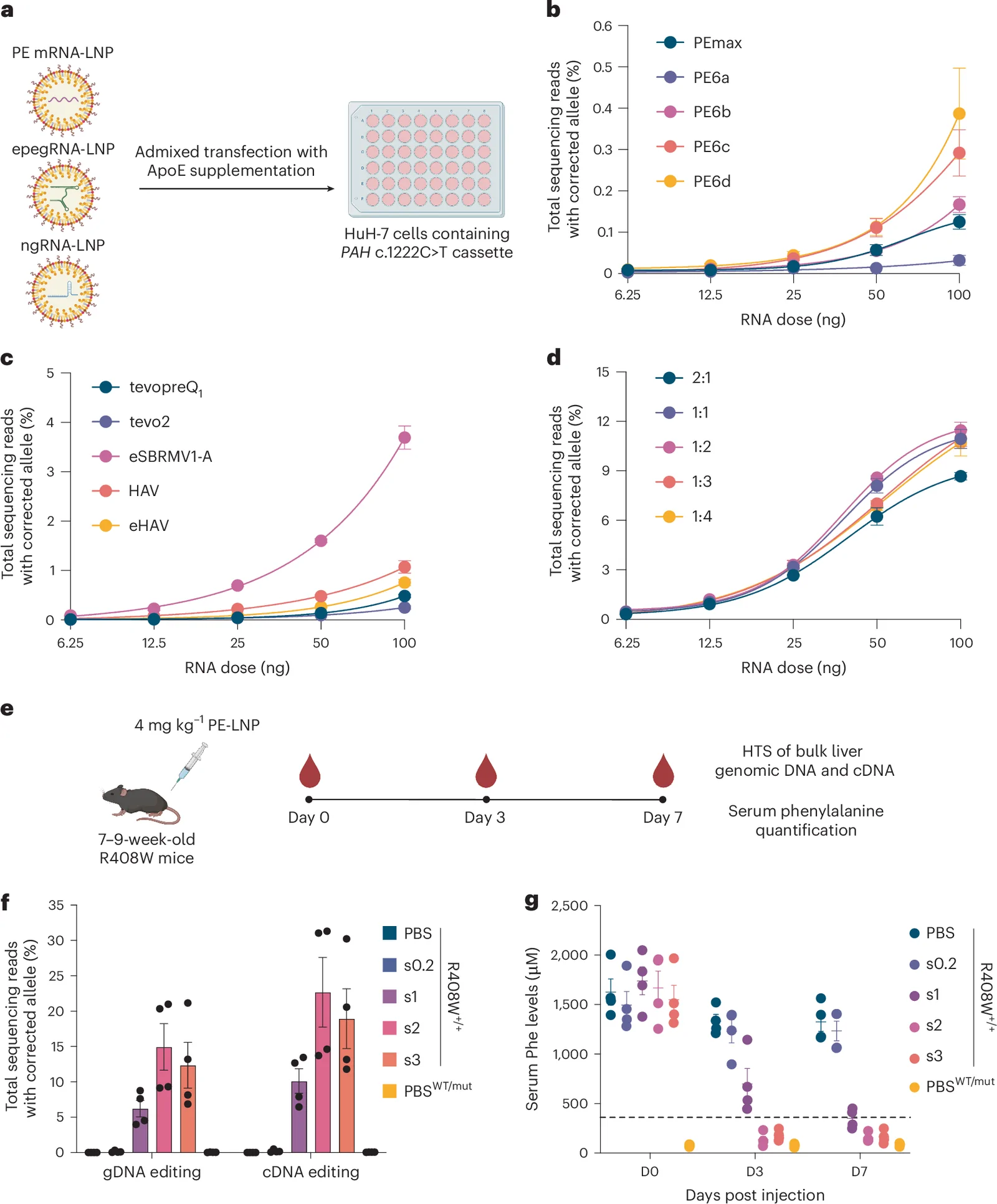
Treatment of PKU in R408W mice using PE-LNPs. **a**, Schematic of the in vitro PE-LNP optimization workflow using HuH-7 cells with a lentivirally integrated *PAH* R408W cassette to develop a lead PE-LNP strategy. **b**, Prime editing efficiencies in lentivirally integrated *PAH* R408W HuH-7 cells treated with PE-LNPs encapsulating either PEmax or a PE6 variant mRNA (PE6a through PE6d), epegRNA or ngRNA. Cells were collected after 72 h and analysed by HTS. **c**, Prime editing efficiencies in lentivirally integrated *PAH* R408W HuH-7 cells treated with PE-LNPs encapsulating epegRNAs with either a 3′ tevopreQ_1_ motif or recently developed 3′ motifs. Cells were collected after 72 h and analysed by HTS. **d**, Prime editing efficiencies in lentivirally integrated *PAH* R408W HuH-7 cells treated with varied ratios of mRNA-:gRNA-LNPs (constant ratio of 9:1 for epegRNA-:ngRNA-LNP). Cells were collected after 72 h and analysed by HTS. **e**, Schematic of the in vivo evaluation of PE-LNPs for the rescue of PKU in R408W mice along with tissue and serum collections. **f**, Prime editing efficiencies in the bulk liver of 7–9-week-old R408W mice dosed via RO injection with either 4 mg kg^−1^ s0.2–s3 PE-LNPs or PBS. PBS-injected *Pah* R408W^WT/+^ mice (PBS^WT/mut^) were included as a reference for serum phenylalanine levels. Tissue from livers was collected 7 days after the injection, and genomic DNA and cDNA from bulk liver were analysed by HTS. **g**, Phenylalanine levels (µM) in serum collected before and 3 and 7 days post-injection from mice treated in **f**. For **b**–**d**, data represent the mean prime editing efficiencies ± s.e.m. of *n* = 3 wells and were fitted to four-parameter logistic curves using nonlinear regression. For **f** and **g**, data are shown as mean ± s.e.m. of *n* = 4 mice per condition (1 male and 3 female). Illustrations created in BioRender: **a**, Jiang, A. https://biorender.com/wmfsknf (2026); **e**, Jiang, A. https://biorender.com/v3xzh3i (2026). Source data

Accordingly, we sought to use PE-LNPs to treat PKU, an inborn error of metabolism caused by mutations in *PAH* that lead to accumulation of phenylalanine (Phe) to neurotoxic levels. Targeting the most frequently occurring pathogenic variant in *PAH* (c. 1222C>T, p.R408W)^47^, we began with a PE3b strategy previously shown to achieve therapeutic levels of editing in mice via AAV delivery^10^ and optimized it for LNP delivery following the workflow developed in this study (Fig. 2i). We systematically evaluated PE variants, 3′ epegRNA motifs and PE-LNP stoichiometry and identified optimal s1, s2 and s3 PE-LNPs through in vitro transfection of HuH-7 cells containing a lentivirally integrated cassette of the diseased allele (Fig. 5a). This approach using a lentivirally integrated target site in cultured cells was also used in the development of k-abe, the BE-LNP used to treat K. J. Muldoon’s *CPS1* deficiency^15^.

Beginning with the baseline PE-LNP stage (s0.2), we formulated OF-02 LNPs separately encapsulating PE variants, tevopreQ_1_ epegRNA and ngRNA and tested them in vitro by admixed transfection (1:0.9:0.1 mRNA:epegRNA:ngRNA by total RNA mass) supplemented with 1 µg ml^−1^ ApoE for enhanced LNP uptake. PE6d was identified as the best PE variant for installation of this edit (Fig. 5b), achieving 0.4% editing in HuH-7 cells at the highest tested dose. Subsequent 3′ epegRNA motif optimization found the eSBRMV1-A motif as the most effective, resulting in 3.7% editing (7.5-fold over tevopreQ_1_; Fig. 5c). Given the modest editing levels observed for this site and the substantial benefit from the use of novel 3′ epegRNA motifs, we reasoned that synthetic epegRNA purity may contribute to PE-LNP editing efficiency for less favourable prime edits and switched to using HPLC-purified epegRNAs from GenScript following in vitro validation of their enhanced performance (Supplementary Fig. 3). We hypothesize that standard chemical synthesis of (e)pegRNAs generates truncated, nonfunctional products that, without stringent purification, carry over into LNP formulations and thereby reduce the fraction of functional epegRNA-LNPs delivered. Finally, we developed s3 PE-LNPs by evaluating a range of mRNA-to-gRNA ratios and identified the optimal 1:2 mRNA:gRNA ratio to yield 11% at the highest tested dose in cultured cells (Fig. 5d), consistent with the importance of epegRNA abundance for robust transient prime editing.

Having developed s1, s2 and s3 PE-LNPs for correction of *PAH* c.1222C>T, we next evaluated their cumulative improvements in vivo by treating homozygous, humanized *Pah* exon 12 transgenic mice harbouring the c.1222C>T variant (referred to as R408W mice). We elected to use this mouse model to demonstrate disease rescue with PE-LNPs because the mouse model accurately recapitulates key characteristics of PKU, such as elevated serum levels of Phe^10^. We administered a single admixed 4 mg kg^−1^ PE-LNP dose via RO injection to 7–9-week-old R408W mice and collected bulk livers after 1 week (Fig. 5e). We used a 4 mg kg^−1^ dose to maximize editing given that this dose was tolerated in mice (Fig. 4b) and that correcting this R408W mutation in vitro resulted in modest levels of prime editing (Fig. 5b–d). HTS revealed 15% and 12% editing of genomic DNA in the bulk liver for s2 and s3 PE-LNPs, respectively (Fig. 5f). We also harvested RNA from bulk livers and generated complementary DNA (cDNA) to assess correction of Pah transcripts, observing an average of 23% cDNA editing for s2 PE-LNPs and 19% for s3 PE-LNPs (Fig. 5f). These findings are consistent with preferential transfection of hepatocytes, the main source of Pah transcripts in the liver^48^.

**Extended Data Fig. 8:**
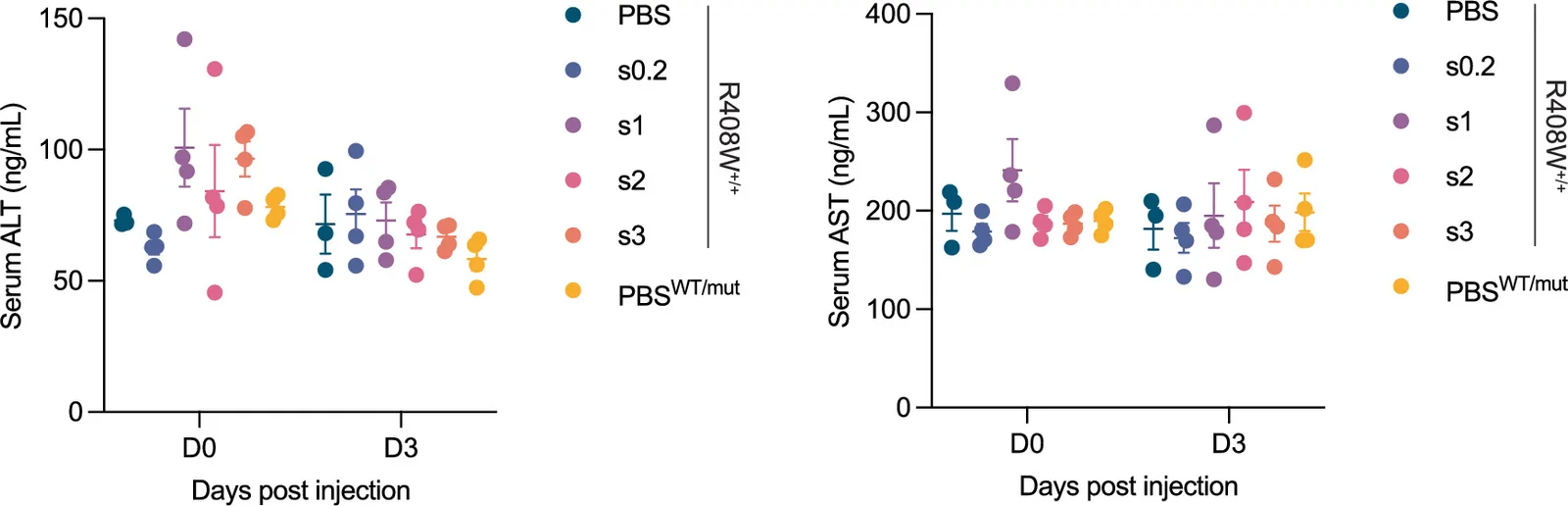
ALT and AST levels in serum following PE-LNP delivery to PKU mice. Concentrations of ALT (left) and AST (right) in the serum of PKU *PAH* R408W^+/+^ mice following treatment with either 4 mg/kg PE-LNPs to install the *PAH* + 3-6 TTGG-to-ACGT edit or PBS via RO injection. PBS-injected *PAH* R408W^WT/+^ mice (PBS WT/mut) were included as a phenotypically normal control group. Data points represent individual mice, and error bars represent mean ± s.e.m. of *n* = 3 mice for PBS-treated group, *n* = 4 mice for all other conditions. Source Data

To assess disease rescue and LNP toxicity, we measured serum Phe, ALT and AST before treatment and on day 3; Phe was also measured on day 7 post-injection during necropsy. PE-LNPs led to an editing-dependent reduction in Phe levels, with s2 PE-LNPs lowering Phe by 90% within 3 days of administration (Fig. 5g). By day 7, Phe levels fell below the threshold of recommended therapeutic intervention (360 µM) in all PE-LNP treated groups except the s0.2 PE-LNPs^49^. We did not observe increased ALT and AST levels at day 3 compared with levels at day 0 (Extended Data Fig. 8). Overall, these results demonstrate that our iterative workflow for PE-LNP optimization streamlines achieving therapeutically relevant levels of prime editing in preclinical models. In addition, the rapid reduction in Phe levels highlights the potential of PE-LNPs for fast and effective rescue of genetic liver diseases.

## La-fused PEs for LNP mediated prime editing in vivo

**Extended Data Fig. 9:**
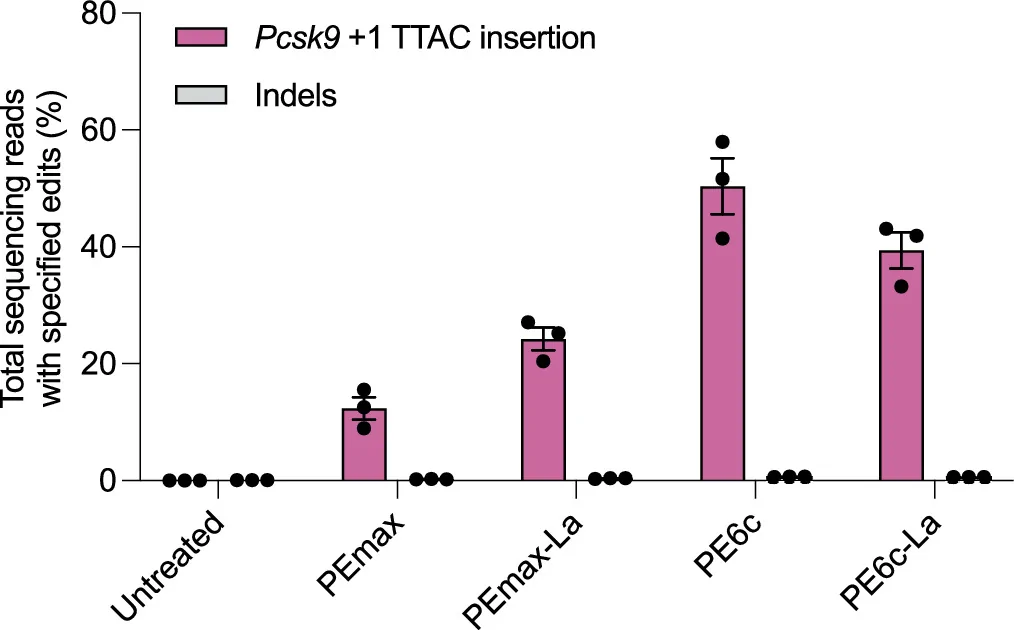
Evaluation of PE7-LNPs in mice. Prime editing efficiencies in the bulk liver of 6-week-old female C57BL/6 mice dosed via RO injection with either 2 mg/kg PE7-LNPs (1 mg/kg of either PEmax-La or PE6c-La mRNA, 0.9 mg/kg La-accessible pegRNA, and 0.1 mg/kg ngRNA) or 2 mg/kg PE-LNPs (1 mg/kg of either PEmax or PE6c mRNA, 0.9 mg/kg eSBRMV1-A motif epegRNA, and 0.1 mg/kg ngRNA), all installing the *Pcsk9* + 1 TTAC insertion edit. Liver tissue was harvested one week after injection and analyzed by HTS. Data points represent individual mice, and error bars represent mean ± s.e.m. of *n* = 3 mice. Source Data

The use of La-fused PEs (PE7 variants) has recently been shown to improve in vivo prime editing via LNPs^12^. We therefore compared in vivo prime editing of PE-LNPs optimized by our workflow to LNPs incorporating La fusions (PE7-LNPs) as an alternative to the use of 3′ epegRNA motifs. We administered 2 mg kg^−1^ PE-LNPs (PEmax or PE6c mRNA, eSBRMV1-A epegRNA and ngRNA) or PE7-LNPs (PEmax-La or PE6c-La mRNA, La-accessible pegRNA and ngRNA) (1:0.9:0.1 mRNA:(e)pegRNA:ngRNA RNA mass ratio for all conditions) with in-house produced mRNAs and GenScript HPLC-purified (e)pegRNAs to C57BL/6 mice via RO injection and collected bulk livers 1 week after injection for HTS analysis of the *Pcsk9* locus. We observed higher average editing efficiencies in mice treated with PE6c mRNA and eSBRMV1-A epegRNA (s2 PE-LNPs, 50% average editing) compared with those treated with PE6c-La mRNA and La-accessible pegRNA (39% average editing) (Extended Data Fig. 9). These data suggest that, although PE7 can increase editing over variants without La in some contexts (such as when using the older PEmax PE), the use of 3′ epegRNA motifs with PE6 variants can yield similar or greater improvements.

We speculate that La fusion may confer benefits that overlap with some benefits of 3′ epegRNA motifs, such as exonuclease protection and enhanced PE complex formation, as indicated by PE7 pegRNA editing efficiencies approaching those achieved with epegRNAs^25^. We did not further investigate the use of PE7 in combination with a La-accessible epegRNA as some of the benefits provided by 3′ epegRNA motifs may be mutually exclusive with those provided by La fusion to the PE due to competing effects (for example, steric interference); indeed, epegRNAs did not outperform pegRNAs when using La-fused PEs at all but one site in a previous report^25^.

## Characterization of PE-LNP off-target editing in vitro and in vivo

**Fig. 6:**
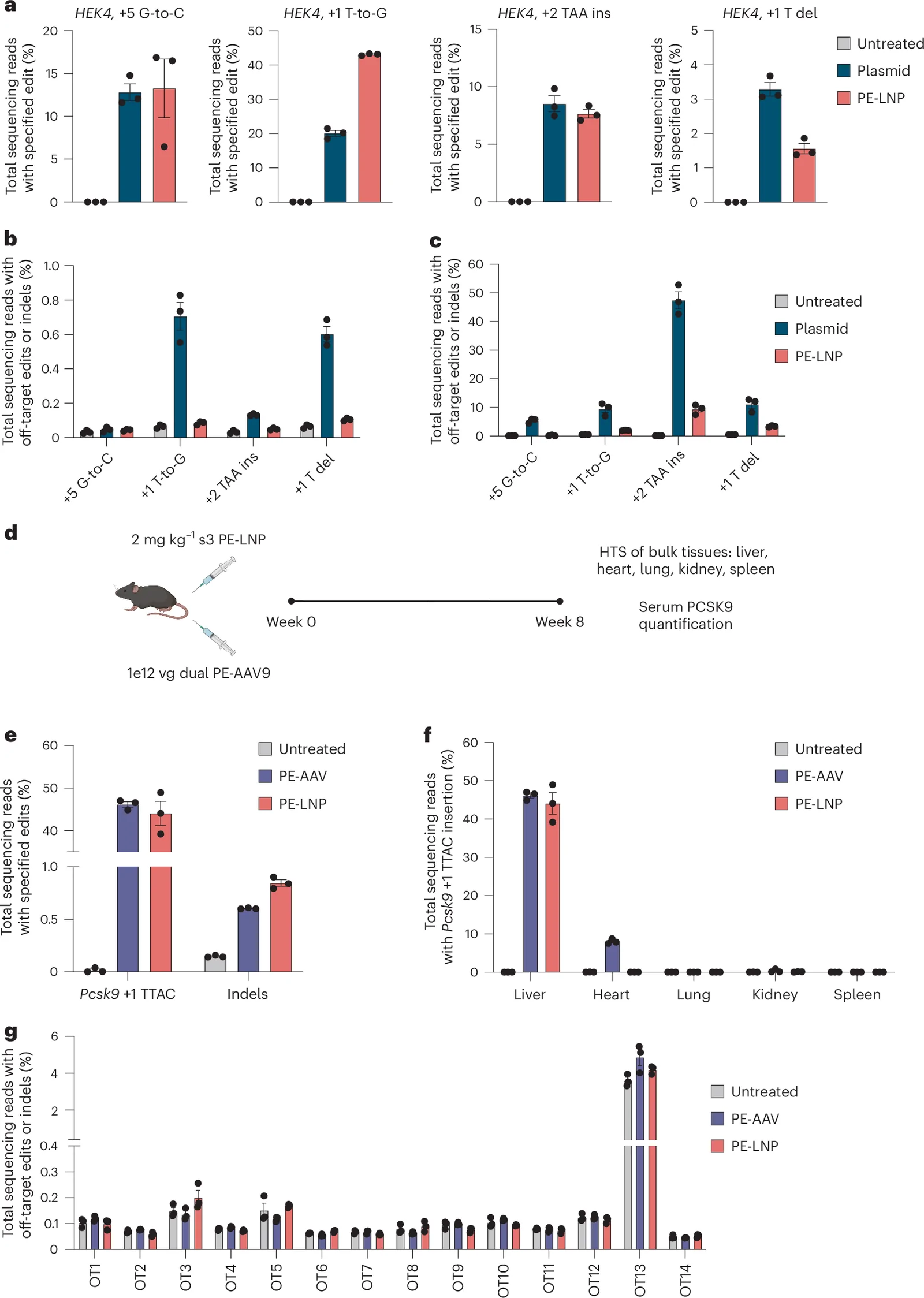
Characterization of PE-LNP off-target editing in vitro and in vivo. **a**, Prime editing efficiencies for four different *HEK4* prime edits in plasmid- and PE-LNP-transfected HuH-7 cells. Cells were collected after 7 days for analysis by HTS. **b**, Percentage of sequencing reads with off-target editing or indels at a known off-target locus (OT1) for the *HEK4* spacer. **c**, Percentage of sequencing reads with off-target editing or indels at a known off-target locus (OT3) for the *HEK4* spacer. **d**, In vivo experimental schematic comparing s3 PE-LNPs and dual PE-AAV9 to evaluate on-target, off-target and off-tissue editing for both delivery modalities. Tissue and serum were collected 8 weeks after injection. **e**, Prime editing efficiencies in the bulk liver of 6-week-old female C57BL/6 mice RO injected with either 2 mg kg^−1^ s3 PE-LNP or 1e12 vg dual PE-AAV9 installing the *Pcsk9* +1 TTAC insertion. **f**, Prime editing efficiencies in five different bulk tissues of mice receiving either 2 mg kg^−1^ s3 PE-LNP or 1e12 vg dual PE-AAV9. **g**, Percentage of sequencing reads with off-target editing or indels at 14 previously reported CIRCLE-seq-nominated candidate off-target sites for both treatment groups and untreated controls. Off-target edits and indels were assessed by targeted amplicon HTS 8 weeks after treatment. For **a**–**c**, data are shown as the mean ± s.e.m. of *n* = 3 wells. For **e**–**g**, data are shown as the mean ± s.e.m. of *n* = 3 mice. Illustrations in **d** created in BioRender; Jiang, A. https://biorender.com/q36qw21 (2026). Source data

LNPs offer several key advantages over viral vectors for gene editing agent delivery, including reduced immunogenicity and transient editor production, which minimizes the opportunity for off-target editing^9,30,31,50–53^. To further assess off-target editing with PE-LNP delivery, we compared plasmid transfection with PE-LNPs in HuH-7 cells across a panel of four prime edits at HEK293T site 4^54^ (hereafter referred to as *HEK4*) with known off-target editing^1^. All PE components were formulated into separate LNPs and used in-house PE6c mRNA and the eSBRMV1-A 3′ epegRNA motif as identified from *Pcsk9* optimization. Wild-type HuH-7 cells were transfected with either plasmid-encoded PE (750 ng PE6c and 250 ng epegRNA) via lipofection or PE-LNPs (200 ng total RNA, 1:2 mRNA:epegRNA). After 1 week, genomic DNA was collected for targeted amplicon sequencing of the on-target site (*HEK4*), as well as its two known off-target sites^1,9^: OT1, within an intron of a lncRNA, and OT3, within an intron of *CTBP2*. PE-LNPs achieved comparable or greater levels of precise on-target editing for three of the four *HEK4*-targeting epegRNAs compared with plasmid transfection (Fig. 6a). Whereas plasmid transfection resulted in significant levels of off-target editing for three of the four epegRNAs at OT1 (Fig. 6b, *P* ≤ 0.0385, two-way ANOVA with post-hoc Tukey) and all four epegRNAs at OT3 (Fig. 6c, *P* ≤ 0.0046) compared with untreated controls, significant off-target editing with PE-LNPs was observed only for the +2 TAA insertion at OT3 (*P* < 0.0001). Furthermore, for this off-target site and epegRNA, the frequency of off-target editing was significantly higher in plasmid-transfected cells than that of those treated with PE-LNP (*P* < 0.0001). Together, these data suggest that transient prime editing delivery indeed lowers the frequency of off-target editing and highlight the advantage of PE-LNPs in enabling transient prime editing with high on-target efficiency and reduced off-target events.

**Extended Data Fig. 10:**
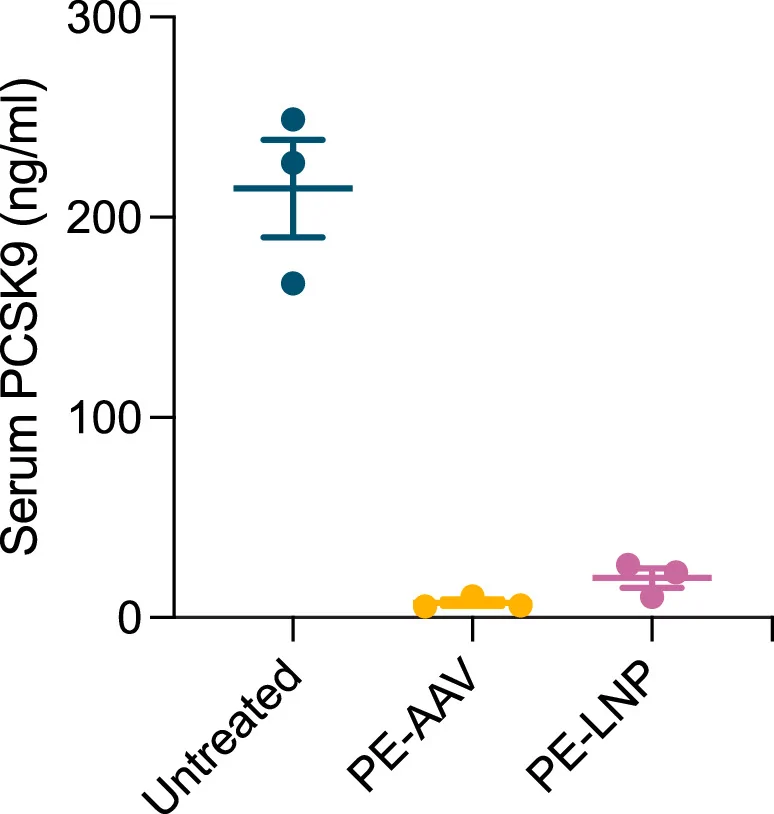
PCSK9 levels in serum of mice treated with s3 PE-LNP or PE-AAV. Concentration of PCSK9 protein in serum collected from mice 8 weeks after RO injection of either 2 mg/kg PE-LNPs or 1e12 vg dual PE-AAV to install the *Pcsk9* + 1 TTAC insertion. Data points represent individual mice, and error bars represent mean ± s.e.m. of *n* = 3 mice. Source Data

To benchmark s3 PE-LNPs against AAVs, a commonly used vehicle to deliver gene therapies and gene editors^5,10,55^, we administered 2 mg kg^−1^ PE-LNPs or 1e12 vg dual PE-AAV9 via RO injection in adult female C57BL/6 mice to mediate the *Pcsk9* +1 TTAC insertion (Fig. 6d). We collected bulk liver, heart, lung, kidneys, spleen and serum 8 weeks post-injection to ensure maximal editing was achieved by both delivery systems and assessed editing by HTS. On-target editing in the bulk liver was comparably high for PE-LNPs and dual PE-AAV9 (44% and 46%, respectively, *P* = 0.1419, two-way ANOVA with post-hoc Tukey’s test) (Fig. 6e), with PE-LNPs and dual PE-AAV9 reducing serum PCSK9 levels on average by 91% and 96%, respectively (Extended Data Fig. 10). In contrast to the highly liver-specific prime editing with s3 PE-LNPs, dual PE-AAV9 also produced 7.9% *Pcsk9* editing in the heart, consistent with the expected tropism of this serotype^56^ (Fig. 6f).

Finally, we sought to identify if either s3 PE-LNPs or dual PE-AAV9 led to above-background off-target editing in the bulk liver. We assayed 14 previously reported CIRCLE-seq-nominated candidate off-target loci for the *Pcsk9* target^33^ by targeted amplicon sequencing 8 weeks after PE treatment and observed off-target editing for PE-LNPs and dual PE-AAV9 at only one site, OT13 (Fig. 6g). However, dual PE-AAV9 off-target editing at OT13 was significantly greater than that of PE-LNPs (*P* < 0.0001), once again demonstrating the benefit of transient editor delivery. Although a greater frequency of off-target events were previously observed for this spacer using AAV-delivered base editing^33^, PEs have been reported to generate fewer off-target edits than other CRISPR editing methods owing to the larger number of prerequisites for productive editing, including pegRNA spacer•target DNA hybridization, pegRNA primer binding site•nicked target strand hybridization, reverse transcription of the RNA template and 3′ DNA flap•unedited DNA strand hybridization^1,57–61^. Collectively, these results highlight the ability of optimized PE-LNPs to achieve efficient on-target prime editing while minimizing the frequency of off-target editing.

## Conclusions

By systematically identifying and addressing bottlenecks to prime editing in vivo mediated by LNPs, we developed PE-LNPs capable of potent and specific prime editing in the mouse liver. We find that the choice of PE and 3′ epegRNA motif can yield substantial improvements to in vivo prime editing when using LNPs, while the ratio of the three RNA-LNP components influences the efficiency of in vivo editing to a more modest extent. In addition, the use of dsRNA-free mRNAs and HPLC-purified epegRNAs further enhanced prime editing in vivo. These findings informed the design of a systematic workflow for PE-LNP optimization, which we hope can aid in the development of PE-LNPs for therapeutic applications. We used this workflow to generate a PE-LNP system that yielded 49% average prime editing at *Pcsk9* in the bulk mouse liver, a 63-fold improvement in editing compared with our initial approach and comparable to 46% achieved with dual-AAV PE delivery.

We further applied the PE-LNP development workflow to the correction of inborn errors of metabolism, in which optimized PE-LNPs demonstrated curative levels of editing in a mouse model of PKU *PAH* c.1222C>T. Notably, this outcome was achieved with a single administration of PE-LNPs, rather than the multidose regimens previously reported for in vivo prime editing with LNPs^12^, and represents an important advance towards the streamlined clinical application of PE-LNPs to treat genetic diseases of the liver.

While these PE-LNPs have demonstrated therapeutically relevant levels of editing in vivo, additional developments in RNA cargoes and LNPs could further improve PE-LNP performance. Advances in mRNA transcript design and synthesis, including sequence optimization^62^ and chemical cap and tail modifications^63,64^, could increase intracellular PE abundance and enhance PE-LNP efficacy. Improvements to epegRNA synthesis, including chemical synthesis methods to improve epegRNA purity^65^ and incorporate chemical modifications to enhance epegRNA stability following intracellular delivery^66,67^, may also benefit PE-LNPs. In addition to these cargo optimizations, our nanoscale characterization results suggests that tailoring LNP formulations to the distinct physicochemical properties of large mRNA versus small gRNA cargoes could provide another opportunity for future improvement. Lastly, ongoing efforts to achieve efficient LNP delivery to tissues beyond the liver will broaden the use of PE-LNPs to study and treat genetic diseases.

## Methods

### General molecular biology

For all general-purpose cloning experiments, primers were ordered from Integrated DNA Technologies (IDT) and editor, IVT plasmids and epegRNA plasmids were generated by Gibson assembly using NEBuilder HiFi DNA Assembly Master Mix (New England Biolabs, E2621). rAAV vector plasmids were cloned by restriction digestion followed by Gibson assembly with eBlocks (IDT). All plasmids used for mammalian tissue culture were purified from Mach1 or NEB Stable *Escherichia coli* using Plasmid Plus Maxiprep or Midiprep kits (Qiagen, 12963 or 12943). PCR was conducted using Phusion U Green Hot Start II DNA polymerase, and the resulting products were purified using the QIAquick PCR Purification Kit or from 1% agarose gel using the QIAquick Gel Extraction Kit (Qiagen).

### Cell culture

Hepa1-6 cells (American Type Culture Collection, CRL-1830) and HuH-7 cells (Cytion, 300156) were grown in Dulbecco’s modified Eagle medium (DMEM) plus GlutaMAX (Thermo Fisher Scientific, 10566016) supplemented with 10% (v/v) fetal bovine serum (FBS) at 37 °C with 5% CO_2_. Cell lines were authenticated by their suppliers and were verified to be mycoplasma negative during the study.

### Generation of in vitro-transcribed mRNA

Plasmid templates for IVT of PE mRNA carry an inactivated T7 promoter, 5′ UTR, Kozak sequence, coding sequence and 3′ UTR (Addgene #178113). IVT templates were generated from template plasmids using NEBNext High-Fidelity 2× PCR Master Mix (New England Biolabs, M0541) with PCR primers that repair an inactive T7 promoter and install a 119 nt poly(A) tail. The PCR template product was purified using QIAquick PCR purification kit (Qiagen, 28104) and then used as an IVT template with the HiScribe T7 High Yield RNA synthesis kit (New England Biolabs, E2040) following the manufacturer’s optional protocol, with full replacement of UTP with N1-methylpseudouridine-5′-triphosphate (TriLink, N-1081) and cotranscriptional capping by CleanCap Reagent AG 3′ OMe (TriLink, N-7413). Reactions were incubated at 37 °C for 2 h, DNase I treated (New England Biolabs, M0303) and purified using the Monarch Spin RNA Cleanup kit (500 µg; New England Biolabs, T2050). mRNAs were dissolved in nuclease-free water and stored at −80 °C. mRNAs sourced from GenScript contain proprietary 5′ and 3′ UTRs and were depleted of dsRNA contaminants by HPLC purification.

### Chemically synthesized gRNA generation

Chemically synthesized pegRNAs and epegRNAs were ordered from IDT (desalted) and GenScript (HPLC-purified) and contained 2′-OMe modifications and 3′-phosphorothioate linkages at the first and last three nucleotides unless stated otherwise. pegRNAs were purified with standard desalting while epegRNAs were either desalted or HPLC-purified. Chemically synthesized ngRNAs were ordered from Synthego and contained 2′-OMe modifications at the first three and last three nucleotides and 3′-phosphorothioate linkages between the first three and last two nucleotides.

### LNP formulation

LNPs were formulated by mixing an aqueous phase containing the mRNA with an ethanol phase containing the lipids in a microfluidic chip device. The ethanol phase was prepared by solubilizing a mixture of OF-02 ionizable lipid (WuXi AppTec), 1,2-dioleoyl-*sn*-glycero-3-phosphoethanolamine (DOPE, Avanti Research, 850725), Chol (Sigma-Aldrich, C8667) and 1,2-dimyristoyl-sn-glycero-3-phosphoethanolamine-*N*-[methoxy(PEG)-2000] (ammonium salt) (C14-PEG2000, Avanti Research, 880150) at a ratio of OF-02/DOPE/Chol/C14-PEG2000 35/16/46.5/2.5 and an ionizable lipid to RNA weight ratio of 10:1 (corresponding to an N/P ratio of 10.1). For SM-102 formulations, the ethanol phase was prepared by solubilizing a mixture of SM-102 (Cayman Chemical), 1,2-distearoyl-*sn*-glycero-3-phosphocholine (DSPC, Avanti Research, 850365), Chol, and 1,2-dimyristoyl-rac-glycero-3-methoxypolyethylene glycol-2000 (DMG-PEG2000, Avanti Research, 880151) at a ratio of SM-102/DSPC/Chol/DMG-PEG2000 50/10/38.5/1.5 and an N/P ratio of 6:1. For MC-3 formulations, the ethanol phase was prepared by solubilizing a mixture of MC-3 (Cayman Chemical), DSPC, Chol and DMG-PEG2000 at the same lipid and N/P ratios as the SM-102 formulation. The aqueous phase was prepared in a 10 mM citrate buffer with corresponding mRNA, pegRNA or ngRNA. The aqueous and ethanol phases were drawn into glass syringes (Hamilton 1000 series) and mixed in a polydimethylsiloxane, herringbone microfluidic device^68,69^ at a 3:1 ratio by syringe pumps (Chemyx Fusion 4000x) to a final RNA concentration of 0.15 mg ml^−1^. The pumps were operated at 900 µl min^−1^ and 300 µl min^−1^ for the aqueous and ethanol phases, respectively. The resultant formulation was dialysed against 1× phosphate-buffered saline (PBS) overnight in a 20 K molecular weight cut-off (MWCO) dialysis cassette (ThermoFisher, 66003) at 4 °C. Following dialysis, LNPs were concentrated at 4 °C with a 100 K MWCO Amicon Ultra Centrifugal Filter (Millipore, UFC210024). LNPs were admixed at specified RNA mass ratios by pipette mixing immediately before injection or transfection.

### LNP characterization

Total RNA concentrations of LNP solutions and LNP sizes were measured by ultraviolet–visible spectrophotometry and DLS, respectively, on the Stunner (Unchained Labs) using the ‘RNA-LNP Screen’ application with default settings in the Stunner Client software (version 9.1.0.143). Samples were measured in duplicate with water blanking and PBS buffer. Data analysis was performed using the Stunner Analysis software (version 9.1.0.157). RNA encapsulation efficiencies were measured using a modified Quanti-iT Ribogreen RNA assay (Invitrogen, R11491)^69^.

### Plasmid transfection of HuH-7 cells

HuH-7 cells were seeded on 48-well poly-D-lysine coated plates at a density of 35,000 cells per well in 300 µl of medium (DMEM with GlutaMAX + 10% FBS). After 24 h, cells were transfected with 1 µl of Lipofectamine 2000 (Thermo Fisher Scientific, 11668027) according to the manufacturer’s protocols, along with 750 ng of PE plasmid and 250 ng of epegRNA plasmid. Seven days after transfection, genomic DNA was collected from cells. Medium was removed from each well, and cells were washed with PBS. Then, 100 µl of lysis buffer (10 mM Tris–HCl pH 8.0, 0.05% sodium dodecyl sulfate and 25 μg ml^−1^ proteinase K) was then added to cells and incubated for 1 h at 37 °C. Following incubation, the lysate was incubated at 80 °C for 30 min and used as input for HTS preparation.

### LNP transfection of HuH-7 and Hepa1-6 cells

Twenty-four hours before transfection, Hepa1-6 and HuH-7 cells were seeded on 48-well poly-D-lysine-coated plates at a density of 30,000 cells per well in 300 µl of medium (DMEM with GlutaMAX + 10% FBS). PE-LNPs at specified total RNA doses were incubated for 10 min at 37 °C with 7 µg ml^−1^ of recombinant apolipoprotein E3 (R&D Systems, 4144-AE). Then, 50 µl of PE-LNP was added to the media in each well. Unless otherwise noted, genomic DNA was collected from cells 72 h after transfection, and the lysate was used as input for HTS preparation.

### HTS and data analysis

Genomic loci of interest were amplified from isolated genomic DNA or cDNA via two rounds of PCR with PhusionU polymerase (Thermo Fisher Scientific, F564). The initial PCR step (PCR1) was done using primers with Illumina adapter overhangs (Supplementary Table 3) with the following conditions: 98 °C for 3 min; 30 cycles of 98 °C for 15 s, 61 °C for 20 s and 72 °C for 30 s, followed by 72 °C for 2 min. Unique Illumina sequencing barcodes were added in the subsequent PCR2 step, using 1 μl of PCR1 as a template with the following conditions: 98 °C for 3 min; 10 cycles of 98 °C for 15 s, 61 °C for 20 s and 72 °C for 30 s, followed by 72 °C for 2 min. After PCR2, samples were pooled according to amplicon size and gel purified in a 1% agarose gel using a QIAquick Gel Extraction Kit (Qiagen, 28704). Pooled library concentration was quantified with the Qubit dsDNA HS assay kit (Thermo Fisher Scientific, Q32851) and run on an Illumina MiSeq 300 V2 Kit with 220–300 cycles.

HTS reads were demultiplexed using MiSeq Reporter (Illumina). Data analysis was conducted using CRISPResso2 in homology-directed repair (HDR) mode. CRISPResso2 analysis was performed with ‘-q30’, ‘discard_indel_reads TRUE’, and the quantification window ‘qwc’ was set to encompass at least ten nucleotides upstream and downstream of the pegRNA and/or ngRNA nick site. Prime editing efficiency was calculated as the percentage of HDR-aligned reads without indels divided by the total number of reference-aligned reads. Indel frequency was calculated as the number of discarded reads divided by the total number of reference-aligned reads.

Off-target analysis for the *HEK4* and *Pcsk9* targets was performed. In brief, reads were aligned to reference off-target amplicons using CRISPResso2 with parameters ‘-q 30’, ‘w 25’ and ‘discard_indel_reads TRUE’. Off-target reads were called as leniently as possible to capture all potential reverse-transcription products. To quantify potential pegRNA-templated off-target editing, the nucleotide sequence 3′ of the PE nick site was compared with the 3′ DNA flap sequence encoded by the pegRNA reverse transcription template. The minimum sequence of the 3′ DNA flap that deviates from the target sequence was designated as an off-target marker sequence. All reference-aligned reads that contain this off-target marker sequence 3′ of the PE nick site were classified as off-target edits, and pegRNA-templated off-target editing was calculated as the percentage of (reads containing the off-target marker sequence)/(reference-aligned reads). The frequency of indels at off-target sites was quantified as the percentage of (discarded reads)/(reference-aligned reads). Total off-target editing was calculated as the sum of pegRNA-templated off-target editing and indel frequency.

### Animal care

All experiments involving live animals were approved by either the Broad Institute Institutional Animal Care and Use Committee (0048-04-15-3; 0255-08-19) or the Institutional Animal Care and Use Committee at the University of Pennsylvania (protocol #805887). Six- to 12-week-old, female C57BL/6J mice were purchased from The Jackson Laboratory (000664). Homozygous humanized PKU mice, as well as heterozygous humanized non-PKU mice, were generated as littermates/colonymates via timed breeding, in some cases using wild-type C57BL/6J mice. Mouse housing facilities were maintained at 20–22 °C with 30–50% humidity, on a 12-h light–dark cycle with ad libitum access to standard rodent diet and water. Animals were randomly assigned to experimental groups.

### RO and intraperitoneal injections

For RO injections, anaesthesia was first induced with 4% isoflurane. Following induction, the right eye was protruded and the needle of the loaded insulin syringe, bevel facing away from the eye, was inserted into the retrobulbar sinus and the LNP or AAV was slowly injected. Following injection, a drop of proparacaine hydrochloride ophthalmic solution (Patterson Veterinary, 07-892-9554) was applied to the eye as an analgesic. For mice receiving DEX, 100 µl of 1 mg ml^−1^ DEX sodium phosphate (Sigma-Aldrich, PHR1768) in 1× PBS was injected intraperitoneally with an insulin syringe 30 min before LNP administration.

### Mice tissue and blood collection

Mice were euthanized by CO_2_ asphyxiation and perfused with PBS via the left ventricle. For genomic DNA extraction and downstream HTS sample preparation of C57BL/6 mice, bulk tissues were collected, transferred to 2-ml tubes containing metal beads (Revvity, 19-670) and mechanically lysed using a TissueLyser II (Qiagen). Genomic DNA was extracted from ground tissues using the DNAdvance kit (Beckman Coulter, A48705) according to the manufacturer’s protocol. The extracted genomic DNA was used as input for downstream HTS sample preparation. For epegRNA and PE protein quantification, as well as genomic DNA and RNA extraction from PKU mice, bulk livers were transferred to bead tubes, snap-frozen in liquid nitrogen and cryogenically homogenized using a Geno Grinder 2010. Homogenized livers were stored at −80 °C until further analysis. Genomic DNA extractions from these samples were performed as described above using the DNAdvance kit. To collect serum, blood was drawn from mice at specified timepoints by either submandibular bleeding or cardiac puncture and transferred into BD microtainer serum separation tubes (Becton Dickinson, 365967) at specified timepoints. Serum was isolated by centrifugation (10 min, 1,000*g*, 4 °C) and stored at −20 °C. Serum PCSK9 (BioLegend, 443207), ALT (Abcam, ab282882) and AST (Abcam, ab263882) were measured by ELISA following manufacturer protocols. Serum Phe was measured by the Penn Metabolomics Core. Whole blood for genomic DNA extraction was collected via cardiac puncture and transferred into BD microtainer EDTA tubes (Becton Dickinson, 365974). Then, 200 µl of blood was incubated in 3 ml of ACK lysis buffer (ThermoFisher, A1049201) for 3 min at room temperature, followed by quenching with 10 ml of PBS. The samples were centrifuged at 500*g* for 9 min, and the supernatant was discarded. This lysis and centrifugation process was repeated once more on the resulting pellet. Genomic DNA was extracted from the final pellet using the Beckman DNAdvance kit.

### Complementary DNA synthesis

To generate cDNA from RNA in the bulk liver of PKU mice for HTS analysis, RNA was first extracted from cryogenically homogenized livers using the AllPrep DNA/RNA/Protein Mini kit (Qiagen, 80004) following the manufacturer’s protocol. cDNA was synthesized from the extracted RNA using ProtoScript II with a random primer mix (New England Biolabs, E6560) according to the manufacturer’s protocol and was used as input for HTS sample preparation.

### RT–qPCR of epegRNA

For RT–qPCR analysis of synthetic epegRNA in the bulk liver of C57BL/6 mice, RNA was first extracted from homogenized livers using a miRNeasy kit (Qiagen, 217084) following the manufacturer’s protocol. cDNA was generated using ProtoScript II from the extracted RNA with a random primer mix (New England Biolabs, E6560) following the manufacturer’s protocol. RT–qPCR of cDNA was then performed^66^. In brief, the PCR solution was prepared as follows: 1 µl of cDNA, 5 µl of TaqPath ProAmp Master Mix (ThermoFisher, A30865), two TaqMan MGB probes (0.1 µl of each, ThermoFisher): a 6-carboxyfluorescein (FAM)-labelled probe for epegRNA and a VIC-labelled probe for U6 snRNA, 0.05 µl of each 100 µM primer to amplify the target epegRNA and endogenous control U6 cDNA, and 3.6 µl of nuclease-free water. RT–qPCR was performed on a CFX Opus 96 Real-Time PCR System (Bio-Rad) with the following amplification protocol: 95 °C for 10 min, followed by 40 cycles of 95 °C for 15 s and 60 °C for 1 min with fluorescent reading performed after the annealing/extension step. PCR ΔCt values were calculated relative to U6 and normalized to the respective motif’s mean ΔCt at the 1-h timepoint to obtain ΔΔCt values. Relative epegRNA levels were determined using the 2^−ΔΔCt^ method. Primer and probe sequences are provided in Supplementary Table 4.

### ELISA of PE protein

Homogenized livers were lysed using high-salt RIPA buffer with phenylmethanesulfonyl fluoride solution (Sigma-Aldrich, 93482) and Halt protease and phosphatase inhibitor (Thermo Fisher Scientific, 78440). Protein concentration in lysate was measured by Pierce BCA (Thermo Fisher Scientific, A55865). ELISA was performed using a FastScan Cas9 ELISA Kit (Cell Signaling Technology, 29666) according to the manufacturer’s protocol with 23 µg of protein as input. Absorbance was measured on a Tecan Spark plate reader. A standard curve of Cas9 protein concentration was included and prepared by serial dilution of Cas9 nuclease protein (New England Biolabs, M0386).

### Cryo-EM sample preparation and imaging

LNPs separately encapsulating mRNA, epegRNA and ngRNA were freshly prepared and concentrated via Amicon filtration as described above to 550 ng µl^−1^ of total RNA in solution. Grid preparation for 300-mesh R2/1 Quantifoil Holey Carbon Cu grids was conducted by glow discharge for 60 s at a set current of 25 mA using a K100X Glow Discharger (EM Sciences). Plunge-freezing was performed using a Vitrobot Mark IV (Thermo Fisher Scientific) set at force 4, 4 °C and 95% humidity; within this instrument, 3 μl of LNP sample was drop-cast on a glow-discharged grid and then immediately blotted for 6 or 9 s before plunge-freezing in liquid ethane cooled by liquid nitrogen. Sample grids were then placed in liquid nitrogen for storage.

Cryo-EM was performed using a Talos Arctica G2 (Thermo Fisher Scientific) operating at 200 kV and with a Falcon 3 direct electron detector. Focusing was carried out on carbon film adjacent to a hole, and five images at 92,000× magnification were taken across the area of each hole. Calibrated pixel sizes were 1.548 Å. All datasets were acquired automatically using EPU software (version 2.12.1.2782REL) (Thermo Fisher Scientific).

### Cryo-EM image analysis

Upon visual inspection of cryo-EM images, LNP cores were classified into one of the following types: lamellar, hexagonal, Janus, multiple-Janus, single-bleb and multiple-bleb cores. Classification and sizing were performed manually in ImageJ Fiji (version 2.16.0, National Institutes of Health). To minimize the potential for selection bias, every discernible LNP and LNP bleb in the set of cryo-EM images was classified and sized.

### Fluorescent labelling of RNAs

For the synthetic epegRNA, a short unmodified polyA stretch was appended to the original epegRNA. The polyA-epegRNA was modified using T4 RNA ligase in the presence of 0.1 mM IR750 labelled cytidine-5′-phosphate-3′-(6-aminohexyl)phosphate (Jena BioScience, NU-1706-IR750) following the manufacturer’s instructions. The crude mixture was then desalted, concentrated and subjected to reversed-phase HPLC purification (acetonitrile as the organic phase, 1 M hexylammonium acetate as the aqueous phase) on an Agilent 1260 Infinity II HPLC (Agilent Openlab CDS)^63^. For the PE6c mRNA labelling, the in vitro-transcribed mRNA was modified by poly(U) Polymerase (NEB, M0337S) with modified nucleotide 5-(3-aminoallyl)-UTP followed by conjugation with Alexa Fluor 647 NHS Ester (Succinimidyl Ester) (Lumiprobe, 26820) following the manufacturer’s protocol. The modified PE6c mRNA was then purified with an oligo-dT column, ethanol precipitated and resuspended in water.

### Ex vivo imaging of organs by IVIS

One hour after RO injection with OF-02 LNPs containing fluorescently labelled RNAs at specified doses, mice were anaesthetized with 5% isoflurane and euthanized via transcardial perfusion with 1× PBS through the left ventricle. The liver, lungs, heart, spleen and kidneys were then excised and imaged using an IVIS spectrum system and analysed with Living Image software. Fluorescence was sequentially captured using specific filter sets: the AF647-mRNA signal was acquired using the Cy5 filter set (640 nm excitation/ 680 nm emission) and the IR750-epegRNA signal was acquired using the 2-DG 750 filter set (745 nm excitation/ 800 nm emission).

### FACS of mouse liver cells

Mouse livers were collected and immediately placed in ice-cold DMEM. The livers were digested using the Liver Dissociation Kit (Miltenyi Biotec, 130-105-807) according to the manufacturer’s instructions. Following digestion, cells were passed through a MACS Smart Strainer (100 μm), and the strainer was washed with 5 ml of DMEM. Cells were centrifuged at 400*g* for 5 min to obtain a cell pellet. The cell pellet was resuspended in 3 ml of ACK lysis buffer at room temperature for 3 min and quenched with 20 ml of 1× PBS. Cells were centrifuged at 400*g* for 5 min to obtain a cell pellet. Cells were live-dead stained with Zombie NIR (1:1000 dilution) in 1× PBS for 30 min at 4 °C and washed once with FACS buffer (1× PBS with 2 mM EDTA and 1% bovine serum albumin). Cells were incubated with TruStain FcX (anti-mouse CD16/32) antibody in FACS buffer for 10 min at room temperature before proceeding directly to antibody staining. Cells were then stained with the following antibody fluorophore conjugates for 30 min at room temperature: FITC-CD45.2 (BioLegend 109805, 1:50 dilution), APC-CD31 (BioLegend, 102410, 1:50) and PE-F4/80 (BioLegend, 123110, 1:50). Cells were sorted into FACS buffer on the BD FACSDiscover S8 with CellView instrument. Sorted cells were centrifuged at 500*g* for 5 min, and genomic DNA was extracted from the pellet using the Beckman DNAdvance kit.

### Generation of HuH-7 cell line harbouring PAH R408W variant using lentivirus

To generate a lentiviral construct with the *PAH* R408W variant for integration into HuH-7 cells, a DNA cassette was designed in silico containing an array of mutated loci associated with genetic metabolic diseases along with 50 bp of flanking endogenous sequence on each side of the variants. The complete list of variants and the full lentiviral cassette sequence can be found in Supplementary Sequence 6. The gene fragment was ordered from a commercial vendor, TOPO cloned (Thermo Fisher Scientific, 450245), digested with BsaI-HF (New England Biolabs, R3733S) and ligated into BsmBI-digested LentiGuide-Puro backbone (Addgene, 53963) using T4 DNA ligase (New England Biolabs, M0202S).

To produce lentivirus, HEK293T cells were cultured in high-glucose DMEM growth medium (Corning, 10-013-CV), 10% FBS (Thermo Fisher Scientific, 16000044) and 1% penicillin–streptomycin (Thermo Fisher Scientific, 15140122) at 37 °C with 5% CO_2_. Transfection was performed with TransIT-LT1 Transfection Reagent (Mirus Bio, MIR2300), psPAX2 (Addgene #12260), pMD2.G (Addgene #12259) and the lentiviral cassette plasmid described above. Viral medium was collected at 18, 48 and 72 h after transfection. Viral medium was centrifuged to remove packaging cells, passed through a 0.45-μm polyvinylidene fluoride filter, and precipitated for 72 h at 4 °C with PEG8000 solution. Viral particles were pelleted and resuspended in 100 μl PBS per 10-cm dish.

Before lentivirus transduction, HuH-7 cells were cultured in low-glucose DMEM growth medium (Gibco, 11885-084), 10% FBS (Thermo Fisher Scientific, 16000044) and 1% penicillin–streptomycin (Thermo Fisher Scientific, 15140122) at 37 °C with 5% CO_2_. The transgene was transduced into HuH-7 cells with 8 µg ml^−1^ polybrene (Santa Cruz, sc-134220) and incubated for 24 h before selection with 1.5 µg ml^−1^ puromycin (Gibco, 1159). Insertion of the exogenous DNA cassette was then verified by sequencing.

### Statistical analysis

Data are presented as mean and standard error of the mean (s.e.m.). Sample sizes are described in the figure legends. No statistical methods were used to predetermine sample size. Unless otherwise noted, *P* values reported are derived from one-way ANOVA with post-hoc Tukey’s comparison for multiple testing. Statistical analysis was performed using GraphPad Prism software.

### Reporting summary

Further information on research design is available in the Nature Portfolio Reporting Summary linked to this article.

## Online content

Any methods, additional references, Nature Portfolio reporting summaries, source data, extended data, supplementary information, acknowledgements, peer review information; details of author contributions and competing interests; and statements of data and code availability are available at https://doi.org/10.1038/s41565-026-02200-6.

## Supplementary information

Supplementary Information Supplementary Figs. 1–3, Notes 1 and 2, Sequences 1–6, Tables 1–5 and References.

Reporting Summary

Supplementary Tables 1–5 Oligonucleotide and off-target HTS sequences.

Supplementary Data Source data for Supplementary Fig. 3.

## Source data

Source Data Figs. 1–6 and Extended Data Figs. 1–10 Raw data.

## Data Availability

All data supporting the results of this study are available within the Article and its Supplementary Information. HTS data are available from the NCBI Sequence Read Archive database (PRJNA1339036). Sequences of PE variants for IVT are listed in the Supplementary Information. Source data are provided with this paper.

## Code availability

The code used for analysis of HTS data is available via GitHub at https://github.com/pinellolab/CRISPResso2.

## References

[1] Anzalone, A. V. et al. Search-and-replace genome editing without double-strand breaks or donor DNA. *Nature* **576**, 149–157 (2019). 31634902 10.1038/s41586-019-1711-4 PMC6907074

[2] Heath, J. M. et al. Prime editing efficiently and precisely corrects causative mutation in chronic granulomatous disease, restoring myeloid function: toward development of a prime edited autologous hematopoietic stem cell therapy. *Blood* **142**, 7129–7129 (2023).

[3] Everette, K. A. et al. Ex vivo prime editing of patient haematopoietic stem cells rescues sickle-cell disease phenotypes after engraftment in mice. *Nat. Biomed. Eng.* **7**, 616–628 (2023). 37069266 10.1038/s41551-023-01026-0 PMC10195679

[4] Li, C. et al. In vivo HSC prime editing rescues sickle cell disease in a mouse model. *Blood* **141**, 2085–2099 (2023). 36800642 10.1182/blood.2022018252 PMC10163316

[5] Sousa, A. A. et al. In vivo prime editing rescues alternating hemiplegia of childhood in mice. *Cell* **188**, 4275–4294 (2025). 40695277 10.1016/j.cell.2025.06.038 PMC12702498

[6] Qin, H. et al. Vision rescue via unconstrained in vivo prime editing in degenerating neural retinas. *J. Exp. Med.* **220**, e20220776 (2023). 36930174 10.1084/jem.20220776 PMC10037108

[7] Fu, Y. et al. In vivo prime editing rescues photoreceptor degeneration in nonsense mutant retinitis pigmentosa. *Nat. Commun.* **16**, 2394 (2025). 40064881 10.1038/s41467-025-57628-6 PMC11893901

[8] Jang, H. et al. Application of prime editing to the correction of mutations and phenotypes in adult mice with liver and eye diseases. *Nat. Biomed. Eng.* **6**, 181–194 (2021). 34446856 10.1038/s41551-021-00788-9

[9] An, M. et al. Engineered virus-like particles for transient delivery of prime editor ribonucleoprotein complexes in vivo. *Nat. Biotechnol.* **42**, 1526–1537 (2024). 38191664 10.1038/s41587-023-02078-y PMC11228131

[10] Brooks, D. L. et al. Efficient in vivo prime editing corrects the most frequent phenylketonuria variant, associated with high unmet medical need. *Am. J. Hum. Genet.* **110**, 2003–2014 (2023). 37924808 10.1016/j.ajhg.2023.10.005 PMC10716342

[11] Böck, D. et al. In vivo prime editing of a metabolic liver disease in mice. *Sci. Transl. Med.* **14**, eabl9238 (2022). 35294257 10.1126/scitranslmed.abl9238 PMC7614134

[12] Rothgangl, T. et al. Treatment of a metabolic liver disease in mice with a transient prime editing approach. *Nat. Biomed. Eng.* **9**, 1705–1718 (2025). 40394220 10.1038/s41551-025-01399-4 PMC12532708

[13] Liu, P. et al. Improved prime editors enable pathogenic allele correction and cancer modelling in adult mice. *Nat. Commun.* **12**, 2121 (2021). 33837189 10.1038/s41467-021-22295-w PMC8035190

[14] Gillmore, J. D. et al. CRISPR–Cas9 in vivo gene editing for transthyretin amyloidosis. *N. Engl. J. Med.* **385**, 493–502 (2021). 34215024 10.1056/NEJMoa2107454

[15] Musunuru, K. et al. Patient-specific in vivo gene editing to treat a rare genetic disease. *N. Engl. J. Med.* **392**, 2235–2243 (2025). 40373211 10.1056/NEJMoa2504747 PMC12713542

[16] Beam Therapeutics. Beam Therapeutics presents additional data for BEAM-302 in alpha-1 antitrypsin deficiency (AATD). In *2025 Alpha-1 Foundation 7th Global Research Conference and 10th Patient Congress* https://investors.beamtx.com/news-releases/news-release-details/beam-therapeutics-presents-additional-data-beam-302-alpha-1 (2025).

[17] Musunuru, K. et al. In vivo CRISPR base editing of PCSK9 durably lowers cholesterol in primates. *Nature* **593**, 429–434 (2021). 34012082 10.1038/s41586-021-03534-y

[18] Hou, X., Zaks, T., Langer, R. & Dong, Y. Lipid nanoparticles for mRNA delivery. *Nat. Rev. Mater.* **6**, 1078–1094 (2021). 34394960 10.1038/s41578-021-00358-0 PMC8353930

[19] Paunovska, K., Loughrey, D. & Dahlman, J. E. Drug delivery systems for RNA therapeutics. *Nat. Rev. Genet.* **23**, 265–280 (2022). 34983972 10.1038/s41576-021-00439-4 PMC8724758

[20] Hosseini-Kharat, M., Bremmell, K. E. & Prestidge, C. A. Why do lipid nanoparticles target the liver? Understanding of biodistribution and liver-specific tropism. *Mol. Ther. Methods Clin. Dev.* **33**, 101436 (2025). 40104152 10.1016/j.omtm.2025.101436 PMC11919328

[21] Akinc, A. et al. Targeted delivery of RNAi therapeutics with endogenous and exogenous ligand-based mechanisms. *Mol. Ther.* **18**, 1357–1364 (2010). 20461061 10.1038/mt.2010.85 PMC2911264

[22] Chen, Z. et al. In vivo prime editing by lipid nanoparticle co-delivery of chemically modified pegRNA and prime editor mRNA. *GEN Biotechnol.* **2**, 490–502 (2023). 39850578 10.1089/genbio.2023.0045 PMC11756591

[23] Liu, P. et al. Increasing intracellular dNTP levels improves prime editing efficiency. *Nat. Biotechnol.* **43**, 539–544 (2024). 39322763 10.1038/s41587-024-02405-x PMC12092096

[24] Nelson, J. W. et al. Engineered pegRNAs improve prime editing efficiency. *Nat. Biotechnol.* **40**, 402–410 (2021). 34608327 10.1038/s41587-021-01039-7 PMC8930418

[25] Yan, J. et al. Improving prime editing with an endogenous small RNA-binding protein. *Nature* **628**, 639–647 (2024). 38570691 10.1038/s41586-024-07259-6 PMC11023932

[26] Doman, J. L. et al. Phage-assisted evolution and protein engineering yield compact, efficient prime editors. *Cell* **186**, 3983–4002 (2023). 37657419 10.1016/j.cell.2023.07.039 PMC10482982

[27] Sakai, H. A. et al. Directed evolution of small RNA-stabilizing motifs that improve prime editing. *Nat. Biotechnol.* 10.1038/s41587-026-03123-2 (2026). 42162366

[28] Fenton, O. S. et al. Bioinspired alkenyl amino alcohol ionizable lipid materials for highly potent in vivo mRNA delivery. *Adv. Mater.* **28**, 2939–2943 (2016). 26889757 10.1002/adma.201505822 PMC5245883

[29] Herrera-Barrera, M. et al. Lipid nanoparticle-enabled intracellular delivery of prime editors. *AAPS J.* **25**, 65 (2023). 37380935 10.1208/s12248-023-00833-2 PMC13198972

[30] Davis, K. M., Pattanayak, V., Thompson, D. B., Zuris, J. A. & Liu, D. R. Small molecule–triggered Cas9 protein with improved genome-editing specificity. *Nat. Chem. Biol.* **11**, 316–318 (2015). 25848930 10.1038/nchembio.1793 PMC4402137

[31] Zuris, J. A. et al. Cationic lipid-mediated delivery of proteins enables efficient protein-based genome editing in vitro and in vivo. *Nat. Biotechnol.* **33**, 73–80 (2014). 25357182 10.1038/nbt.3081 PMC4289409

[32] Rees, H. A. et al. Improving the DNA specificity and applicability of base editing through protein engineering and protein delivery. *Nat. Commun.* **8**, 15790 (2017). 28585549 10.1038/ncomms15790 PMC5467206

[33] Banskota, S. et al. Engineered virus-like particles for efficient in vivo delivery of therapeutic proteins. *Cell* **185**, 250–265 (2022). 35021064 10.1016/j.cell.2021.12.021 PMC8809250

[34] Li, S. et al. Payload distribution and capacity of mRNA lipid nanoparticles. *Nat. Commun.* **13**, 5561 (2022). 36151112 10.1038/s41467-022-33157-4 PMC9508184

[35] Shuto, Y. et al. Structural basis for pegRNA-guided reverse transcription by a prime editor. *Nature* **631**, 224–231 (2024). 38811740 10.1038/s41586-024-07497-8 PMC11222144

[36] Zhang, W. et al. Enhancing CRISPR prime editing by reducing misfolded pegRNA interactions. *Elife* **12**, RP90948 (2024). 38847802 10.7554/eLife.90948 PMC11161173

[37] Baiersdörfer, M. et al. A facile method for the removal of dsRNA contaminant from in vitro-transcribed mRNA. *Mol. Ther. Nucleic Acids* **15**, 26–35 (2019). 30933724 10.1016/j.omtn.2019.02.018 PMC6444222

[38] Chatterjee, S., Kon, E., Sharma, P. & Peer, D. Endosomal escape: a bottleneck for LNP-mediated therapeutics. *Proc. Natl Acad. Sci. USA* **121**, e2307800120 (2024). 38437552 10.1073/pnas.2307800120 PMC10945858

[39] Hassett, K. J. et al. Optimization of lipid nanoparticles for intramuscular administration of mRNA vaccines. *Mol. Ther. Nucleic Acids* **15**, 1–11 (2019). 30785039 10.1016/j.omtn.2019.01.013 PMC6383180

[40] Jayaraman, M. et al. Maximizing the potency of siRNA lipid nanoparticles for hepatic gene silencing in Vivo. *Angew. Chem. Int. Ed.* **51**, 8529–8533 (2012). 10.1002/anie.201203263 PMC3470698 22782619

[41] Cheng, M. H. Y. et al. Induction of bleb structures in lipid nanoparticle formulations of mRNA leads to improved transfection potency. *Adv. Mater.* **35**, 2303370 (2023). 10.1002/adma.202303370 37172950

[42] Finn, J. D. et al. A single administration of CRISPR/Cas9 lipid nanoparticles achieves robust and persistent in vivo genome editing. *Cell Rep.* **22**, 2227–2235 (2018). 29490262 10.1016/j.celrep.2018.02.014

[43] Li, L. et al. Glucocorticoid pre-administration improves LNP-mRNA mediated protein replacement and genome editing therapies. *Int. J. Pharm.* **672**, 125282 (2025). 39880143 10.1016/j.ijpharm.2025.125282

[44] Andries, O. et al. N1-methylpseudouridine-incorporated mRNA outperforms pseudouridine-incorporated mRNA by providing enhanced protein expression and reduced immunogenicity in mammalian cell lines and mice. *J. Control. Release* **217**, 337–344 (2015). 26342664 10.1016/j.jconrel.2015.08.051

[45] Brooks, D. L. et al. A base editing strategy using mRNA-LNPs for in vivo correction of the most frequent phenylketonuria variant. *Hum. Genet. Genom. Adv.* **5**, 100253 (2024). 10.1016/j.xhgg.2023.100253 PMC10800763 37922902

[46] Lee, R. G. et al. Efficacy and safety of an investigational single-course CRISPR base-editing therapy targeting PCSK9 in nonhuman primate and mouse models. *Circulation* **147**, 242–253 (2023). 36314243 10.1161/CIRCULATIONAHA.122.062132

[47] Hillert, A. et al. The genetic landscape and epidemiology of phenylketonuria. *Am. J. Hum. Genet.* **107**, 234–250 (2020). 32668217 10.1016/j.ajhg.2020.06.006 PMC7413859

[48] van Spronsen, F. J. et al. Phenylketonuria. *Nat. Rev. Dis. Primers* **7**, 36 (2021). 34017006 10.1038/s41572-021-00267-0 PMC8591558

[49] Vockley, J. et al. Phenylalanine hydroxylase deficiency: diagnosis and management guideline. *Genet. Med.* **16**, 188–200 (2014). 24385074 10.1038/gim.2013.157

[50] Kim, S., Kim, D., Cho, S. W., Kim, J. & Kim, J.-S. Highly efficient RNA-guided genome editing in human cells via delivery of purified Cas9 ribonucleoproteins. *Genome Res.* **24**, 1012–1019 (2014). 24696461 10.1101/gr.171322.113 PMC4032847

[51] Ramakrishna, S. et al. Gene disruption by cell-penetrating peptide-mediated delivery of Cas9 protein and guide RNA. *Genome Res.* **24**, 1020–1027 (2014). 24696462 10.1101/gr.171264.113 PMC4032848

[52] Cameron, P. et al. Mapping the genomic landscape of CRISPR–Cas9 cleavage. *Nat. Methods* **14**, 600–606 (2017). 28459459 10.1038/nmeth.4284

[53] Lattanzi, A. et al. Optimization of CRISPR/Cas9 delivery to human hematopoietic stem and progenitor cells for therapeutic genomic rearrangements. *Mol. Ther.* **27**, 137–150 (2019). 30424953 10.1016/j.ymthe.2018.10.008 PMC6318785

[54] Tsai, S. Q. et al. GUIDE-seq enables genome-wide profiling of off-target cleavage by CRISPR–Cas nucleases. *Nat. Biotechnol.* **33**, 187–197 (2014). 25513782 10.1038/nbt.3117 PMC4320685

[55] Davis, J. R. et al. Efficient prime editing in mouse brain, liver and heart with dual AAVs. *Nat. Biotechnol.* **42**, 253–264 (2023). 37142705 10.1038/s41587-023-01758-z PMC10869272

[56] Bish, L. T. et al. Adeno-associated virus (AAV) serotype 9 provides global cardiac gene transfer superior to AAV1, AAV6, AAV7, and AAV8 in the mouse and rat. *Hum. Gene Ther.* **19**, 1359–1368 (2008). 18795839 10.1089/hum.2008.123 PMC2940566

[57] Habib, O., Habib, G., Hwang, G.-H. & Bae, S. Comprehensive analysis of prime editing outcomes in human embryonic stem cells. *Nucleic Acids Res.* **50**, 1187–1197 (2022). 35018468 10.1093/nar/gkab1295 PMC8789035

[58] Gao, R. et al. Genomic and Transcriptomic analyses of prime editing guide RNA–independent off-target effects by prime editors. *CRISPR J.* **5**, 276–293 (2022). 35294852 10.1089/crispr.2021.0080

[59] Liang, S.-Q. et al. Genome-wide profiling of prime editor off-target sites in vitro and in vivo using PE-tag. *Nat. Methods* **20**, 898–907 (2023). 37156841 10.1038/s41592-023-01859-2 PMC11708963

[60] Fiumara, M. et al. Author Correction: Genotoxic effects of base and prime editing in human hematopoietic stem cells. *Nat. Biotechnol.* **42**, 986–986 (2024). 38273067 10.1038/s41587-024-02142-1 PMC11180604

[61] Kim, D. Y., Moon, S. B., Ko, J.-H., Kim, Y.-S. & Kim, D. Unbiased investigation of specificities of prime editing systems in human cells. *Nucleic Acids Res.* **48**, 10576–10589 (2020). 32941652 10.1093/nar/gkaa764 PMC7544197

[62] Zhang, H. et al. Algorithm for optimized mRNA design improves stability and immunogenicity. *Nature* **621**, 396–403 (2023). 37130545 10.1038/s41586-023-06127-z PMC10499610

[63] Chen, H. et al. Chemical and topological design of multicapped mRNA and capped circular RNA to augment translation. *Nat. Biotechnol.* **43**, 1128–1143 (2024). 39313647 10.1038/s41587-024-02393-y PMC11929619

[64] Chen, H. et al. Branched chemically modified poly(A) tails enhance the translation capacity of mRNA. *Nat. Biotechnol.* **43**, 194–203 (2024). 38519719 10.1038/s41587-024-02174-7 PMC11416571

[65] Lei, X. et al. Rapid generation of long, chemically modified pegRNAs for prime editing. *Nat. Biotechnol.* **43**, 1156–1167 (2024). 39349835 10.1038/s41587-024-02394-x

[66] Ryan, D. E. et al. Phosphonoacetate modifications enhance the stability and editing yields of guide RNAs for Cas9 editors. *Biochemistry* **62**, 3512–3520 (2022). 35436085 10.1021/acs.biochem.1c00768 PMC10734248

[67] Yin, H. et al. Structure-guided chemical modification of guide RNA enables potent non-viral in vivo genome editing. *Nat. Biotechnol.* **35**, 1179–1187 (2017). 29131148 10.1038/nbt.4005 PMC5901668

[68] Chen, D. et al. Rapid discovery of potent siRNA-containing lipid nanoparticles enabled by controlled microfluidic formulation. *J. Am. Chem. Soc.* **134**, 6948–6951 (2012). 22475086 10.1021/ja301621z

[69] Ma, Y., VanKeulen-Miller, R. & Fenton, O. S. mRNA lipid nanoparticle formulation, characterization and evaluation. *Nat. Protoc.* **20**, 2618–2651 (2025). 40069324 10.1038/s41596-024-01134-4 PMC12312782

