# Supplementary material for: Efficient prime editing in vivo and in vitro using lipid nanoparticles
Source: Nat Nanotechnol. 2026 Jun 15;21(7):1022–35. doi: 10.1038/s41565-026-02200-6 (PMC13379318; doi:10.1038/s41565-026-02200-6)
Supplement: Supplementary file 1 — Supplementary Figs. 1–3, Notes 1 and 2, Sequences 1–6, Tables 1–5 and References. [file 41565_2026_2200_MOESM1_ESM.pdf]

nature nanotechnology

Supplementary information https://doi.org/10.1038/s41565-026-02200-6

Efficient prime editing in vivo and in vitro using lipid nanoparticles

In the format provided by the authors and unedited

Supplementary Information

Supplementary Figures.

Supplementary Figure 1. Fast Fourier Transform (FFT) of LNP images obtained from Cryo-EM images.

Supplementary Figure 2. Representative FACS gating strategy for mouse liver cell sorting.

Supplementary Figure 3. Synthetic epegRNA purification and vendor comparison for the PKU *PAH* +3-6 TTGG-to-ACGT edit.

Supplementary Notes.

Supplementary Note 1. mRNA size minimization via intein-split and untethered prime editors.

Supplementary Note 2. Comparison of OF-02, MC3, and SM-102 formulations.

Supplementary Sequences. Sequences of key PE constructs used for IVT and the lentiviral cassette used in this study.

Supplementary Sequence 1. PE6c with La (SSB N-term) fusion.

Supplementary Sequence 2. N-term PE6c for intein-split PE.

Supplementary Sequence 3. C-term PE6c for intein-split PE.

Supplementary Sequence 4. PE6c without RT for untethered PE.

Supplementary Sequence 5. eeTf1 RT from PE6c with N-terminal MCP fusion for untethered PE.

Supplementary Sequence 6. Lentiviral cassette harboring the *PAH* R408W variant.

Supplementary Tables (provided as a separate file):

Supplementary Table 1. Sequences of pegRNAs used in this study.

Supplementary Table 2. Sequences of ngRNAs used in this study.

Supplementary Table 3. Primers used for genomic DNA amplification and their corresponding amplicons.

Supplementary Table 4. Probes and primers used to quantify pegRNA abundance in bulk liver tissues.

Supplementary Table 5. Sequences of *HEK4* and *Pcsk9* pegRNA off-target sites, primers used for amplicon sequencing, and their corresponding amplicons

Supplementary References.

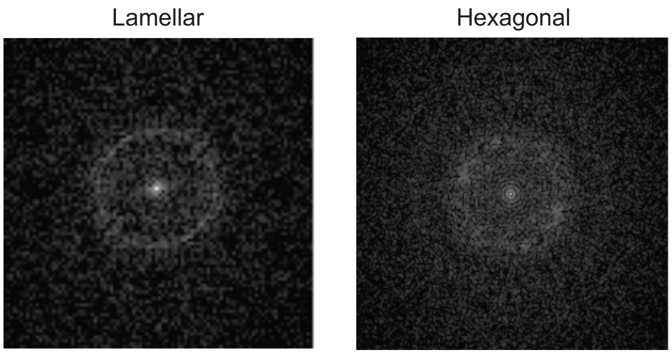

Supplementary Figure 1. Fast Fourier Transform (FFT) of LNP images obtained from cryo-EM images. FFT of LNP images from cryo-EM with lamellar (left) and hexagonal (right) cores. Lamellar cores show a bright ring while hexagonal cores show a hexagonal arrangement of six bright spots.

a

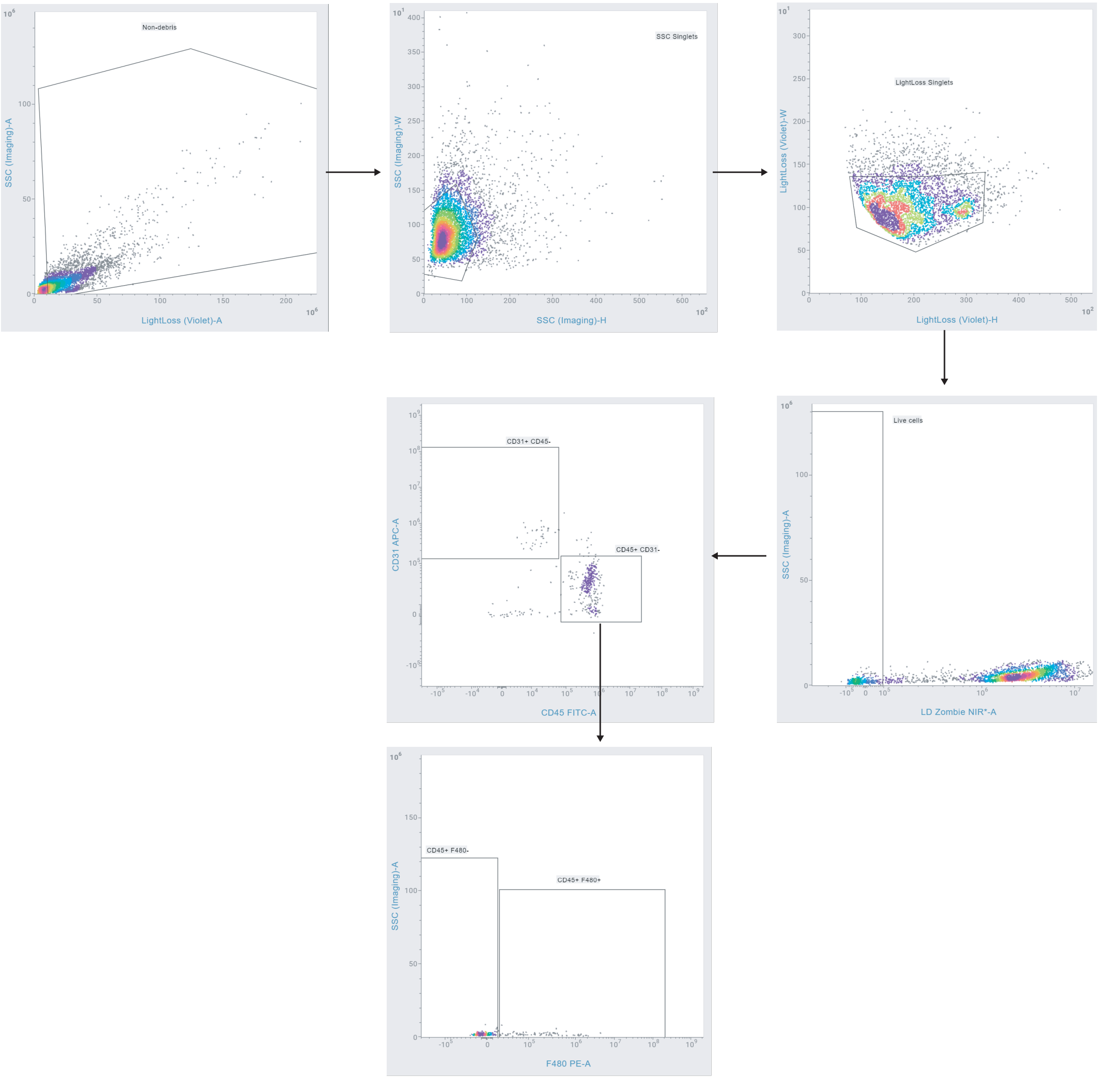

b

Population	Events	% Parent	% Total
All Events	10,000	N/A	100.00 %
Saturated	785	7.85 %	7.85 %
Unsaturated	9,215	92.15 %	92.15 %
Non-debris	3,412	34.12 %	34.12 %
SSC Singlets	2,538	74.38 %	25.38 %
LightLoss Sin...	2,132	84.00 %	21.32 %
Live cells	558	26.17 %	5.58 %
CD31+ CD45-	81	14.52 %	0.81 %
CD45+ CD31-	400	71.68 %	4.00 %
CD45+ F480+	63	15.75 %	0.63 %
CD45+ F480-	336	84.00 %	3.36 %

Supplementary Figure 2. Representative FACS gating strategy for mouse liver cell sorting. a) Within the live cell population, endothelial cells were defined as $CD31^{+}CD45^{-}$. Within the $CD45^{+}CD31^{-}$ population, Kupffer cells were defined as $F4/80^{+}$ and non-Kupffer immune cells were defined as $F4/80^{-}$. b) Population statistics for the representative gating strategy in (a).

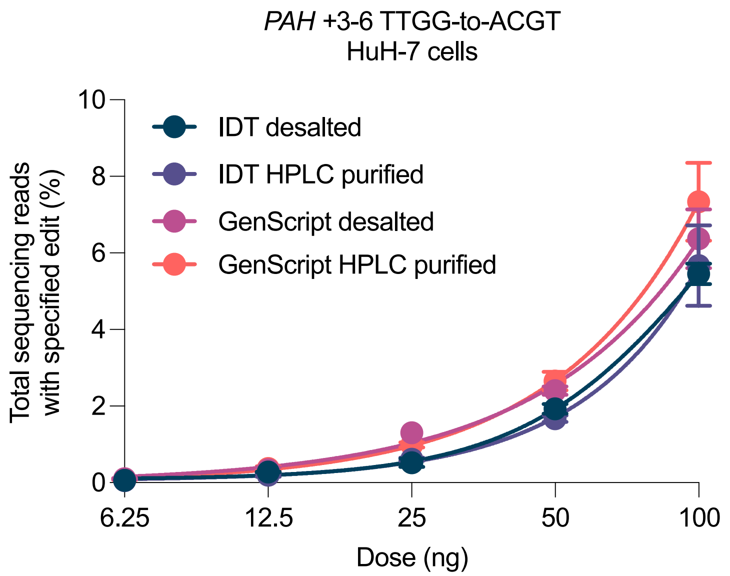

Supplementary Figure 3. Synthetic epegRNA purification and vendor comparison for the PKU *PAH* +3-6 TTGG-to-ACGT edit. Prime editing efficiencies in HuH-7 cells containing the PKU *PAH* R408W cassette after treatment with s2 PE-LNPs. Data points represent the mean prime editing efficiencies ± s.e.m. of n = 3 wells. Cells were harvested after 72 hours for HTS, and data were fitted to four-parameter logistic curves using nonlinear regression.

Supplementary Note 1. mRNA size minimization via intein-split and untethered prime editors.

Larger mRNAs result in fewer mRNA molecules encapsulated per LNP[1], thereby requiring a greater amount of LNP to deliver an equivalent molar amount of cargo. Most formulation studies evaluate functional cargo delivery with mRNAs smaller than 2 kb, such as those encoding luciferase or Cre recombinase, potentially biasing the development of LNP formulations towards those optimized for delivery of cargoes smaller than full-length PE. We therefore tested strategies to minimize the length of PE mRNA as a potential way to further increase PE-LNP editing efficiencies.

We designed size-minimized PE mRNA using two approaches. In the first approach, inspired by dual PE-AAV[2], we split the PE6c coding sequence into two halves, each fused to either the N-terminal or C-terminal intein from *N. punctiforme* and encoded on separate mRNA molecules (referred to as intein-split PE6c). We administered 2 mg/kg PE-LNPs (1:0.9:0.1 mRNA:epegRNA:ngRNA by total RNA mass) installing the *Pcsk9* +1 TTAC insertion to adult C57BL/6 mice via RO injection; PE6c was delivered either as a full-length mRNA encapsulated in a single LNP or as an intein-split mRNA with each half encapsulated in a separate LNP and admixed at a 1:1 total mRNA ratio. The intein-split PE6c system (11% editing) did not improve prime editing in the bulk liver of mice when compared to full-length PE6c (25% editing, P = 0.004), presumably due to incomplete protein trans-splicing for intein-split PE (Extended Data Fig. 3a).

In the second approach, we removed the linker between the SpCas9 nickase and the engineered and evolved Tf1 reverse transcriptase of PE6c (eeTf1 RT) and delivered them as two separate mRNA molecules, one encoding the nickase and the other encoding the RT[3] (referred to as untethered PE6c). To promote colocalization of the RT with the epegRNA, the MS2 coat protein (MCP) was fused to the N-terminus of the eeTf1 RT, and an MS2 RNA stem-loop was inserted in the epegRNA scaffold's ST2 loop as previously described[4]. We administered 2 mg/kg PE-LNPs (1:0.9:0.1 mRNA:epegRNA:ngRNA by total RNA mass) installing the *Pcsk9* +1 TTAC insertion to adult C57BL/6 mice via RO injection; PE6c was delivered either as full-length mRNA in a single LNP or as SpCas9 nickase and eeTf1 RT mRNAs in separate LNPs admixed at

a 1:1 total mRNA ratio. We analyzed prime editing of the bulk liver 7 days after LNP injection and observed that delivery of full-length PE6c mRNA (26% editing) again resulted in greater levels of prime editing when compared to delivery of the size-minimized, untethered PE6c (15% editing, $P = 0.0013$) (Extended Data Fig. 3b). We speculate that the lower efficiency of untethered PE6c may arise from inefficient co-localization of the nickase and RT, insufficient recruitment of the epegRNA to the RT, or a hook effect. All of these challenges could be further exacerbated by the temporal constraints of transient mRNA and epegRNA delivery. Given that neither size minimization approach increased prime editing efficiency in mice, we continued to use mRNA encoding full-length PE.

Supplementary Note 2. Comparison of OF-02, MC3, and SM-102 formulations.

MC3- and SM-102-based LNPs exhibited higher RNA encapsulation efficiencies (Extended Data Fig. 4a), smaller mRNA-LNP sizes (Extended Data Fig. 4b), and generally higher RNA yields following LNP formulation (Extended Data Fig. 4c) when compared to OF-02-based LNPs. However, following treatment of mice with 1.5 mg/kg s3 PE-LNPs using either MC3, SM-102, or OF-02 LNP formulations to install the *Pcsk9* +1 TTAC insertion, we observed higher levels of editing in the bulk liver after one week with OF-02 PE-LNPs (53% average editing) when compared to MC3 (0.8% average editing) or SM-102 (2.5 % average editing) (Extended Data Fig. 4d). This observation is consistent with prior design-of-experiments (DOE) studies demonstrating that higher RNA encapsulation efficiency does not necessarily correlate with improved mRNA delivery *in vivo*[5]. The *in vivo* results are further supported by previous observations that OF-02 demonstrates superior liver mRNA delivery in mice when compared to the benchmark lipid cKK-E12[6], while cKK-E12 has shown greater delivery efficiency than MC3 or Lipid 5, a close structural analog of SM-102[7-9]. Based on these *in vivo* prime editing results, we continued to use OF-02 in the remainder of the study. Further engineering of lipid structures may enable the combination of improved physicochemical properties with the potent RNA delivery efficiency required for *in vivo* prime editing.

Supplementary Sequences. Sequences of key PE constructs used for IVT and the lentiviral cassette in this study.

Supplementary Sequence 1. PE6c with La (SSB N-term) fusion, 6723 bp

Inactive T7 promoter-5’ UTR-SV40NLS-PE6c-XTEN-SSB(N-term)-SV40NLS-cMycNLS-3’ UTR

TAATACGACACACTATAAGGAAATAAGAGAGAAAAGAAGAGTAAGAAGAAATATAA
GAGCCACCATGAAACGGACAGCCGACGGAAGCGAGTTCGAGTCACCAAAGAAGA
AGCGGAAAGTCGACAAGAAGTACAGCATCGGCCTGGACATCGGCACCAACTCTGT
GGGCTGGGCCGTGATCACCGACGAGTACAAGGTGCCCAGCAAGAAATTCAAGGT
GCTGGGCAACACCGACCGGCACAGCATCAAGAAGAACCTGATCGGAGCCCTGCT
GTTCGACAGCGGCGAAACAGCCGAGGCCACCCGGCTGAAGAGAACCGCCAGAAG
AAGATACACCAGACGGAAGAACCGGATCTGCTATCTGCAAGAGATCTTCAGCAAC
GAGATGGCCAAGGTGGACGACAGCTTCTTCCACAGACTGGAAGAGTCCTTCCTGG
TGGAAGAGGATAAGAAGCACGAGCGGCACCCCATCTTCGGCAACATCGTGGACGA
GGTGGCCTACCACGAGAAGTACCCCACCATCTACCACCTGAGAAAGAAACTGGTG
GACAGCACCGACAAGGCCGACCTGCGGCTGATCTATCTGGCCCTGGCCCACATG
ATCAAGTTCCGGGGCCACTTCCTGATCGAGGGCGACCTGAACCCCGACAACAGCG
ACGTGGACAAGCTGTTCATCCAGCTGGTGCAGACCTACAACCAGCTGTTCGAGGA
AAACCCCATCAACGCCAGCGGCGTGGACGCCAAGGCCATCCTGTCTGCCAGACT
GAGCAAGAGCAGAAAGCTGGAAAATCTGATCGCCCAGCTGCCCGGCGAGAAGAA
GAATGGCCTGTTCGGAAACCTGATTGCCCTGAGCCTGGGCCTGACCCCCAACTTC
AAGAGCAACTTCGACCTGGCCGAGGATGCCAAACTGCAGCTGAGCAAGGACACCT
ACGACGACGACCTGGACAACCTGCTGGCCCAGATCGGCGACCAGTACGCCGACC
TGTTTCTGGCCGCCAAGAACCTGTCCGACGCCATCCTGCTGAGCGACATCCTGAG
AGTGAACACCGAGATCACCAAGGCCCCCCTGAGCGCCTCTATGATCAAGAGATAC
GACGAGCACCACCAGGACCTGACCCTGCTGAAAGCTCTCGTGCGGCAGCAGCTG
CCTGAGAAGTACAAAGAGATTTTCTTCGACCAGAGCAAGAACGGCTACGCCGGCT
ACATTGACGGCGGAGCCAGCCAGGAAGAGTTCTACAAGTTCATCAAGCCCATCCT
GGAAAAGATGGACGGCACCGAGGAACTGCTCGTGAAGCTGAAGAGAGAGGACCT
GCTGCGGAAGCAGCGGACCTTCGACAACGGCAGCATCCCCCACCAGATCCACCT
GGGAGAGCTGCACGCCATTCTGCGGCGGCAGGAAGATTTTTACCCATTCCTGAAG
GACAACCGGGAAAAGATCGAGAAGATCCTGACCTTCCGCATCCCCTACTACGTGG
GCCCTCTGGCCAGGGGAAACAGCAGATTCGCCTGGATGACCAGAAAGAGCGAGG
AAACCATCACCCCCTGGAACTTCGAGGAAGTGGTGGACAAGGGCGCTTCCGCCCA
GAGCTTCATCGAGCGGATGACCAACTTCGATAAGAACCTGCCCAACGAGAAGGTG
CTGCCCAAGCACAGCCTGCTGTACGAGTACTTCACCGTGTATAACGAGCTGACCA
AAGTGAAATACGTGACCGAGGGAATGAGAAAGCCCGCCTTCCTGAGCGGCGAGC

AGAAAAAGGCCATCGTGGACCTGCTGTTCAAGACCAACCGGAAAGTGACCGTGAA
GCAGCTGAAAGAGGACTACTTCAAGAAAATCGAGTGCTTCGACTCCGTGGAAATCT
CCGGCGTGGAAGATCGGTTCAACGCCTCCCTGGGCACATACCACGATCTGCTGAA
AATTATCAAGGACAAGGACTTCCTGGACAATGAGGAAAACGAGGACATTCTGGAAG
ATATCGTGCTGACCCTGACACTGTTTGAGGACAGAGAGATGATCGAGGAACGGCT
GAAAACCTATGCCCACCTGTTCGACGACAAAGTGATGAAGCAGCTGAAGCGGCGG
AGATACACCGGCTGGGGCAGGCTGAGCCGGAAGCTGATCAACGGCATCCGGGAC
AAGCAGTCCGGCAAGACAATCCTGGATTTCCTGAAGTCCGACGGCTTCGCCAACA
GAAACTTCATGCAGCTGATCCACGACGACAGCCTGACCTTTAAAGAGGACATCCA
GAAAGCCCAGGTGTCCGGCCAGGGCGATAGCCTGCACGAGCACATTGCCAATCT
GGCCGGCAGCCCCGCCATTAAGAAGGGCATCCTGCAGACAGTGAAGGTGGTGGA
CGAGCTCGTGAAAGTGATGGGCCGGCACAAGCCCGAGAACATCGTGATCGAAATG
GCCAGAGAGAACCAGACCACCCAGAAGGGACAGAAGAACAGCCGCGAGAGAATG
AAGCGGATCGAAGAGGGCATCAAAGAGCTGGGCAGCCAGATCCTGAAAGAACAC
CCCGTGGAAAACACCCAGCTGCAGAACGAGAAGCTGTACCTGTACTACCTGCAGA
ATGGGCGGGATATGTACGTGGACCAGGAACTGGACATCAACCGGCTGTCCGACTA
CGATGTGGACGCTATCGTGCCTCAGAGCTTTCTGAAGGACGACTCCATCGACAAC
AAGGTGCTGACCAGAAGCGACAAGAACCGGGGCAAGAGCGACAACGTGCCCTCC
GAAGAGGTCGTGAAGAAGATGAAGAACTACTGGCGGCAGCTGCTGAACGCCAAG
CTGATTACCCAGAGAAAGTTCGACAATCTGACCAAGGCCGAGAGAGGCGGCCTGA
GCGAACTGGATAAGGCCGGCTTCATCAAGAGACAGCTGGTGGAAACCCGGCAGAT
CACAAAGCACGTGGCACAGATCCTGGACTCCCGGATGAACACTAAGTACGACGAG
AATGACAAGCTGATCCGGGAAGTGAAAGTGATCACCCTGAAGTCCAAGCTGGTGT
CCGATTTCCGGAAGGATTTCCAGTTTTACAAAGTGCGCGAGATCAACAACTACCAC
CACGCCCACGACGCCTACCTGAACGCCGTCGTGGGAACCGCCCTGATCAAAAAGT
ACCCTAAGCTGGAAAGCGAGTTCGTGTACGGCGACTACAAGGTGTACGACGTGCG
GAAGATGATCGCCAAGAGCGAGCAGGAAATCGGCAAGGCTACCGCCAAGTACTTC
TTCTACAGCAACATCATGAACTTTTTCAAGACCGAGATTACCCTGGCCAACGGCGA
GATCCGGAAGCGGCCTCTGATCGAGACAAACGGCGAAACCGGGGAGATCGTGTG
GGATAAGGGCCGGGATTTTGCCACCGTGCGGAAAGTGCTGAGCATGCCCCAAGT
GAATATCGTGAAAAAGACCGAGGTGCAGACAGGCGGCTTCAGCAAAGAGTCTATC
CTGCCCAAGAGGAACAGCGATAAGCTGATCGCCAGAAAGAAGGACTGGGACCCTA
AGAAGTACGGCGGCTTCGACAGCCCCACCGTGGCCTATTCTGTGCTGGTGGTGGC
CAAAGTGGAAAAGGGCAAGTCCAAGAAACTGAAGAGTGTGAAAGAGCTGCTGGGG
ATCACCATCATGGAAAGAAGCAGCTTCGAGAAGAATCCCATCGACTTTCTGGAAGC
CAAGGGCTACAAAGAAGTGAAAAAGGACCTGATCATCAAGCTGCCTAAGTACTCCC
TGTTCGAGCTGGAAAACGGCCGGAAGAGAATGCTGGCCTCTGCCGGCGAACTGC
AGAAGGGAAACGAACTGGCCCTGCCCTCCAAATATGTGAACTTCCTGTACCTGGC
CAGCCACTATGAGAAGCTGAAGGGCTCCCCCGAGGATAATGAGCAGAAACAGCTG
TTTGTGGAACAGCACAAGCACTACCTGGACGAGATCATCGAGCAGATCAGCGAGT

TCTCCAAGAGAGTGATCCTGGCCGACGCTAATCTGGACAAAGTGCTGTCCGCCTA
CAACAAGCACCGGGATAAGCCCATCAGAGAGCAGGCCGAGAATATCATCCACCTG
TTTACCCTGACCAATCTGGGAGCCCCTGCCGCCTTCAAGTACTTTGACACCACCAT
CGACCGGAAGAGGTACACCAGCACCAAAGAGGTGCTGGACGCCACCCTGATCCA
CCAGAGCATCACCGGCCTGTACGAGACACGGATCGACCTGTCTCAGCTGGGAGG
TGACTCCGGCGGAAGCTCTGGTGGCAGCAAGCGGACCGCCGACGGCTCTGAATT
CGAGAGCCCTAAGAAGAAAAGAAAGGTGAGCGGAGGCTCTAGCGGCGGAAGCAT
CAGCAGCTCTAAGCACACCCTGAGCCAGATGAACAAGGTGAGCAACATCGTGAAG
GAACCCGAGCTGCCCGACATCTACAAGGAATTTAAGGACATCACCGCCGACACCA
ATACCGAGAAGCTGCCTAAGCCTATTAAGGGCCTGGAATTTGAAGTGGAACTGACA
CAGGAGAACTACAGACTGCCTATCCGGAACTATCCTCTGACTCCAGTCAAGATGCA
GGCCATGAACGACGAGATCAATCAAGGCCTGAAAGGCGGCATCATCAGAGAGAGC
AAGGCCATCAACGCCTGCCCTGTTATATTCGTGCCCAGAAAGGAAGGCACACTGC
GCATGGTGGTCGACTACAGGCCCCTGAACAAGTACGTGAAGCCCAACGTCTACCC
CCTGCCACTGATTGAACAACTGCTGGCCAAGATCCAGGGCAGCACCATTTTCACC
AAGCTGGACCTGAAAAGCGCCTACCACCAGATCAGAGTGCGAAAGGGAGATGAGC
ACAAGCTGGCCTTCCGGTGCCCCAGAGGAGTCTTCGAGTACCTGGTGATGCCTTA
CGGCATCAAGACAGCCCCTGCCCACTTTCAGTACTTCATCAACACAATCCTGGGC
GAGGCCAAGGAAAGCCACGTGGTGTGCTACATGGACGACATCCTGATCCATTCCA
AGTCCGAGTCCGAACACGTGAAACATGTGAAGGACGTGCTGCAAAAGCTGAAGAA
CGCTAATCTGATCATCAACCAGGCCAAATGCGAGTTTCACCAGAGCCAAGTGAAGT
TCCTGGGCTACCACATCAGCGAGAAGGGCTTAACCCCATGTCAGGAGAACATCGA
CAAGGTGCTGCAGTGGAAACAGCCTAAAAACCAGAAGGAACTGAGACAGTTCCTG
GGCCAGGTGAACTACCTGAGAAAATTCATCCCCAAAACCAGCCAGTTGACCCACC
CTCTGAACAAACTCCTGAAAAAGGATGTCAGATGGAAATGGACCCCTACCCAGACC
CAGGCTATCGAGAATATCAAGCAATGTCTGGTGTCTCCTCCTGTGCTGAGGCACTT
CGACTTCAGCAAGAAGATCCTGCTTGAGACAGACGTTTCTGATGTGGCCGTGGGA
GCCGTGCTGAGCCAGAAGCATGATGATGATAAGTACTACCCTGTGGGCTATTACA
GCGCTAAAATGAGCAAAGCCCAGCTGAATTATTCTGTGTCCGACAAGGAGATGCT
GGCTATCATCAAAAGCCTGGAGCACTGGCGGCACTACCTGGAATCTACAATCGAG
CCCTTCAAGATCCTGACCGACCACAGAAACCTGATTGGAAGAATCACAAACGAGA
GCGAACCAGAGAACAAGCGGCTGGCCAGATGGCAGCTGTTCCTGCAGGACTTCAA
CTTCGAGATCAACTACAGACCTGGCTCTGCAAATCACATCGCCGATGCCCTGTCTA
GAATCGTGGACGAGACTGAGCCTATCCCTAAGGACAACGAAGATAACAGCATCAA
CTTCGTGAACCAGATCAGCATCTCCGGAGGATCTAGCGGAGGCTCCTCTGGCTCT
GAGACACCTGGCACAAGCGAGAGCGCAACACCTGAAAGCAGCGGGGGCAGCAGC
GGGGGGTCAATGGCTGAAAATGGTGATAATGAAAAGATGGCTGCCCTGGAGGCCA
AAATCTGTCATCAAATTGAGTATTATTTTGGCGACTTCAATTTGCCACGGACAAGT
TTCTAAAGGAACAGATAAAACTGGATGAAGGCTGGGTACCTTTGGAGATAATGATA
AAATTCAACAGGTTGAACCGTCTAACAACAGACTTTAATGTAATTGTGGAAGCATTG

AGCAAATCCAAGGCAGAACTCATGGAAATCAGTGAAGATAAAACTAAAATCAGAAG
GTCTCCAAGCAAACCCCTACCTGAAGTGACTGATGAGTATAAAAATGATGTAAAAA
ACAGATCTGTTTATATTAAAGGCTTCCCAACTGATGCAACTCTTGATGACATAAAAG
AATGGTTAGAAGATAAAGGTCAAGTACTAAATATTCAGATGAGAAGAACATTGCATA
AAGCATTTAAGGGATCAATTTTTGTTGTGTTTGATAGCATTGAATCTGCTAAGAAATT
TGTAGAGACCCCTGGCCAGAAGTACAAAGAAACAGACCTGCTAATACTTTTCAAGG
ACGATTACTTTGCCAAAAAAAATGAATCTGGCGGCTCAAAAAGAACCGCCGACGGC
AGCGAATTCGAGTCTCCCAAGAAGAAGAGGAAAGTCGGCTCTGGCCCTGCCGCTA
AGAGAGTGAAGCTGGACTAATTAATTAAGCTGCCTTCTGCGGGGCTTGCCTTCTGG
CCATGCCCTTCTTCTCTCCCTTGCACCTGTACCTCTTGGTCTTTGAATAAAGCCTGA
GTAGGAAG

Supplementary Sequence 2. N-term PE6c for intein-split PE, 3702 bp

Inactive T7 promoter-5' UTR-SV40NLS-N-term PE6c-NpuN-SV40NLS-cMycNLS-3' UTR

TAATACGACACACTATAAGGAAATAAGAGAGAAAAGAAGAGTAAGAAGAAATATAA
GAGCCACCATGAAACGGACAGCCGACGGAAGCGAGTTCGAGTCACCAAAGAAGA
AGCGGAAAGTCGACAAGAAGTACAGCATCGGCCTGGACATCGGCACCAACTCTGT
GGGCTGGGCCGTGATCACCGACGAGTACAAGGTGCCCAGCAAGAAATTCAAGGT
GCTGGGCAACACCGACCGGCACAGCATCAAGAAGAACCTGATCGGAGCCCTGCT
GTTCGACAGCGGCGAAACAGCCGAGGCCACCCGGCTGAAGAGAACCGCCAGAAG
AAGATACACCAGACGGAAGAACCGGATCTGCTATCTGCAAGAGATCTTCAGCAAC
GAGATGGCCAAGGTGGACGACAGCTTCTTCCACAGACTGGAAGAGTCCTTCCTGG
TGGAAGAGGATAAGAAGCACGAGCGGCACCCCATCTTCGGCAACATCGTGGACGA
GGTGGCCTACCACGAGAAGTACCCCACCATCTACCACCTGAGAAAGAAACTGGTG
GACAGCACCGACAAGGCCGACCTGCGGCTGATCTATCTGGCCCTGGCCCACATG
ATCAAGTTCCGGGGCCACTTCCTGATCGAGGGCGACCTGAACCCCGACAACAGCG
ACGTGGACAAGCTGTTCATCCAGCTGGTGCAGACCTACAACCAGCTGTTCGAGGA
AAACCCCATCAACGCCAGCGGCGTGGACGCCAAGGCCATCCTGTCTGCCAGACT
GAGCAAGAGCAGAAAGCTGGAAAATCTGATCGCCCAGCTGCCCGGCGAGAAGAA
GAATGGCCTGTTCGGAAACCTGATTGCCCTGAGCCTGGGCCTGACCCCCAACTTC
AAGAGCAACTTCGACCTGGCCGAGGATGCCAAACTGCAGCTGAGCAAGGACACCT
ACGACGACGACCTGGACAACCTGCTGGCCCAGATCGGCGACCAGTACGCCGACC
TGTTTCTGGCCGCCAAGAACCTGTCCGACGCCATCCTGCTGAGCGACATCCTGAG
AGTGAACACCGAGATCACCAAGGCCCCCCTGAGCGCCTCTATGATCAAGAGATAC
GACGAGCACCACCAGGACCTGACCCTGCTGAAAGCTCTCGTGCGGCAGCAGCTG
CCTGAGAAGTACAAAGAGATTTTCTTCGACCAGAGCAAGAACGGCTACGCCGGCT
ACATTGACGGCGGAGCCAGCCAGGAAGAGTTCTACAAGTTCATCAAGCCCATCCT
GGAAAAGATGGACGGCACCGAGGAACTGCTCGTGAAGCTGAAGAGAGAGGACCT
GCTGCGGAAGCAGCGGACCTTCGACAACGGCAGCATCCCCCACCAGATCCACCT
GGGAGAGCTGCACGCCATTCTGCGGCGGCAGGAAGATTTTTACCCATTCCTGAAG
GACAACCGGGAAAAGATCGAGAAGATCCTGACCTTCCGCATCCCCTACTACGTGG
GCCCTCTGGCCAGGGGAAACAGCAGATTCGCCTGGATGACCAGAAAGAGCGAGG
AAACCATCACCCCCTGGAACTTCGAGGAAGTGGTGGACAAGGGCGCTTCCGCCCA
GAGCTTCATCGAGCGGATGACCAACTTCGATAAGAACCTGCCCAACGAGAAGGTG
CTGCCCAAGCACAGCCTGCTGTACGAGTACTTCACCGTGTATAACGAGCTGACCA
AAGTGAAATACGTGACCGAGGGAATGAGAAAGCCCGCCTTCCTGAGCGGCGAGC
AGAAAAAGGCCATCGTGGACCTGCTGTTCAAGACCAACCGGAAAGTGACCGTGAA
GCAGCTGAAAGAGGACTACTTCAAGAAAATCGAGTGCTTCGACTCCGTGGAAATCT
CCGGCGTGGAAGATCGGTTCAACGCCTCCCTGGGCACATACCACGATCTGCTGAA

AATTATCAAGGACAAGGACTTCCTGGACAATGAGGAAAACGAGGACATTCTGGAAG
ATATCGTGCTGACCCTGACACTGTTTGAGGACAGAGAGATGATCGAGGAACGGCT
GAAAACCTATGCCCACCTGTTCGACGACAAAGTGATGAAGCAGCTGAAGCGGCGG
AGATACACCGGCTGGGGCAGGCTGAGCCGGAAGCTGATCAACGGCATCCGGGAC
AAGCAGTCCGGCAAGACAATCCTGGATTTCCTGAAGTCCGACGGCTTCGCCAACA
GAAACTTCATGCAGCTGATCCACGACGACAGCCTGACCTTTAAAGAGGACATCCA
GAAAGCCCAGGTGTCCGGCCAGGGCGATAGCCTGCACGAGCACATTGCCAATCT
GGCCGGCAGCCCCGCCATTAAGAAGGGCATCCTGCAGACAGTGAAGGTGGTGGA
CGAGCTCGTGAAAGTGATGGGCCGGCACAAGCCCGAGAACATCGTGATCGAAATG
GCCAGAGAGAACCAGACCACCCAGAAGGGACAGAAGAACAGCCGCGAGAGAATG
AAGCGGATCGAAGAGGGCATCAAAGAGCTGGGCAGCCAGATCCTGAAAGAACAC
CCCGTGGAAAACACCCAGCTGCAGAACGAGAAGCTGTACCTGTACTACCTGCAGA
ATGGGCGGGATATGTACGTGGACCAGGAACTGGACATCAACCGGCTGTCCGACTA
CGATGTGGACGCTATCGTGCCTCAGAGCTTTCTGAAGGACGACTCCATCGACAAC
AAGGTGCTGACCAGAAGCGACAAGAACCGGGGCAAGAGCGACAACGTGCCCTCC
GAAGAGGTCGTGAAGAAGATGAAGAACTACTGGCGGCAGCTGCTGAACGCCAAG
CTGATTACCCAGAGAAAGTTCGACAATCTGACCAAGGCCGAGAGAGGCGGCCTGA
GCGAACTGGATAAGGCCGGCTTCATCAAGAGACAGCTGGTGGAAACCCGGCAGAT
CACAAAGCACGTGGCACAGATCCTGGACTCCCGGATGAACACTAAGTACGACGAG
AATGACAAGCTGATCCGGGAAGTGAAAGTGATCACCCTGAAGTCCAAGCTGGTGT
CCGATTTCCGGAAGGATTTCCAGTTTTACAAAGTGCGCGAGATCAACAACTACCAC
CACGCCCACGACGCCTACCTGAACGCCGTCGTGGGAACCGCCCTGATCAAAAAGT
ACCCTAAGCTGGAAAGCGAGTTCGTGTACGGCGACTACAAGGTGTACGACGTGCG
GAAGATGATCGCCAAGTGCCTGTCCTACGAGACAGAGATCCTGACAGTGGAGTAT
GGCCTGCTGCCAATCGGCAAGATCGTGGAGAAGAGGATCGAGTGTACCGTGTACT
CTGTGGATAACAATGGCAACATCTATACACAGCCCGTGGCACAGTGGCACGATAG
GGGAGAGCAGGAGGTGTTCGAGTATTGCCTGGAGGACGGCAGCCTGATCAGGGC
AACCAAGGACCACAAGTTCATGACAGTGGATGGCCAGATGCTGCCCATCGACGAG
ATTTTCGAGCGGGAGCTGGACCTGATGAGAGTGGATAACCTGCCTAATTCTGGCG
GCTCAAAAAGAACCGCCGACGGCAGCGAATTCGAGTCTCCCAAGAAGAAGAGGAA
AGTCGGCTCTGGCCCTGCCGCTAAGAGAGTGAAGCTGGACTAATTAATTAAGCTG
CCTTCTGCGGGGCTTGCCTTCTGGCCATGCCCTTCTTCTCTCCCTTGCACCTGTAC
CTCTTGGTCTTTGAATAAAGCCTGAGTAGGAAG

Supplementary Sequence 3. C-term PE6c for intein-split PE, 3081 bp

Inactive T7 promoter-5’ UTR-SV40NLS-NpuC-C-term PE6c-SV40NLS-cMycNLS-3’ UTR

TAATACGACACACTATAAGGAAATAAGAGAGAAAAGAAGAGTAAGAAGAAATATAA
GAGCCACCATGAAACGGACAGCCGACGGAAGCGAGTTCGAGTCACCAAAGAAGA
AGCGGAAAGTCATCAAGATTGCTACACGGAAATACCTGGGAAAGCAGAACGTGTA
CGACATCGGCGTGGAGCGGGATCACAACTTCGCCCTGAAGAATGGCTTTATCGCC
AGCAATTGTTTCAACGAAATCGGCAAGGCTACCGCCAAGTACTTCTTCTACAGCAA
CATCATGAACTTTTTCAAGACCGAGATTACCCTGGCCAACGGCGAGATCCGGAAG
CGGCCTCTGATCGAGACAAACGGCGAAACCGGGGAGATCGTGTGGGATAAGGGC
CGGGATTTTGCCACCGTGCGGAAAGTGCTGAGCATGCCCCAAGTGAATATCGTGA
AAAAGACCGAGGTGCAGACAGGCGGCTTCAGCAAAGAGTCTATCCTGCCCAAGAG
GAACAGCGATAAGCTGATCGCCAGAAAGAAGGACTGGGACCCTAAGAAGTACGGC
GGCTTCGACAGCCCCACCGTGGCCTATTCTGTGCTGGTGGTGGCCAAAGTGGAAA
AGGGCAAGTCCAAGAAACTGAAGAGTGTGAAAGAGCTGCTGGGGATCACCATCAT
GGAAAGAAGCAGCTTCGAGAAGAATCCCATCGACTTTCTGGAAGCCAAGGGCTAC
AAAGAAGTGAAAAAGGACCTGATCATCAAGCTGCCTAAGTACTCCCTGTTCGAGCT
GGAAAACGGCCGGAAGAGAATGCTGGCCTCTGCCGGCGAACTGCAGAAGGGAAA
CGAACTGGCCCTGCCCTCCAAATATGTGAACTTCCTGTACCTGGCCAGCCACTATG
AGAAGCTGAAGGGCTCCCCCGAGGATAATGAGCAGAAACAGCTGTTTGTGGAACA
GCACAAGCACTACCTGGACGAGATCATCGAGCAGATCAGCGAGTTCTCCAAGAGA
GTGATCCTGGCCGACGCTAATCTGGACAAAGTGCTGTCCGCCTACAACAAGCACC
GGGATAAGCCCATCAGAGAGCAGGCCGAGAATATCATCCACCTGTTTACCCTGAC
CAATCTGGGAGCCCCTGCCGCCTTCAAGTACTTTGACACCACCATCGACCGGAAG
AGGTACACCAGCACCAAAGAGGTGCTGGACGCCACCCTGATCCACCAGAGCATCA
CCGGCCTGTACGAGACACGGATCGACCTGTCTCAGCTGGGAGGTGACTCCGGCG
GAAGCTCTGGTGGCAGCAAGCGGACCGCCGACGGCTCTGAATTCGAGAGCCCTA
AGAAGAAAAGAAAGGTGAGCGGAGGCTCTAGCGGCGGAAGCATCAGCAGCTCTA
AGCACACCCTGAGCCAGATGAACAAGGTGAGCAACATCGTGAAGGAACCCGAGCT
GCCCGACATCTACAAGGAATTTAAGGACATCACCGCCGACACCAATACCGAGAAG
CTGCCTAAGCCTATTAAGGGCCTGGAATTTGAAGTGGAACTGACACAGGAGAACTA
CAGACTGCCTATCCGGAACTATCCTCTGACTCCAGTCAAGATGCAGGCCATGAAC
GACGAGATCAATCAAGGCCTGAAAGGCGGCATCATCAGAGAGAGCAAGGCCATCA
ACGCCTGCCCTGTTATATTCGTGCCCAGAAAGGAAGGCACACTGCGCATGGTGGT
CGACTACAGGCCCCTGAACAAGTACGTGAAGCCCAACGTCTACCCCCTGCCACTG
ATTGAACAACTGCTGGCCAAGATCCAGGGCAGCACCATTTTCACCAAGCTGGACC
TGAAAAGCGCCTACCACCAGATCAGAGTGCGAAAGGGAGATGAGCACAAGCTGGC
CTTCCGGTGCCCCAGAGGAGTCTTCGAGTACCTGGTGATGCCTTACGGCATCAAG

ACAGCCCCTGCCCACTTTCAGTACTTCATCAACACAATCCTGGGCGAGGCCAAGG
AAAGCCACGTGGTGTGCTACATGGACGACATCCTGATCCATTCCAAGTCCGAGTC
CGAACACGTGAAACATGTGAAGGACGTGCTGCAAAAGCTGAAGAACGCTAATCTG
ATCATCAACCAGGCCAAATGCGAGTTTCACCAGAGCCAAGTGAAGTTCCTGGGCT
ACCACATCAGCGAGAAGGGCTTAACCCCATGTCAGGAGAACATCGACAAGGTGCT
GCAGTGGAAACAGCCTAAAAACCAGAAGGAACTGAGACAGTTCCTGGGCCAGGTG
AACTACCTGAGAAAATTCATCCCCAAAACCAGCCAGTTGACCCACCCTCTGAACAA
ACTCCTGAAAAAGGATGTCAGATGGAAATGGACCCCTACCCAGACCCAGGCTATC
GAGAATATCAAGCAATGTCTGGTGTCTCCTCCTGTGCTGAGGCACTTCGACTTCAG
CAAGAAGATCCTGCTTGAGACAGACGTTTCTGATGTGGCCGTGGGAGCCGTGCTG
AGCCAGAAGCATGATGATGATAAGTACTACCCTGTGGGCTATTACAGCGCTAAAAT
GAGCAAAGCCCAGCTGAATTATTCTGTGTCCGACAAGGAGATGCTGGCTATCATCA
AAAGCCTGGAGCACTGGCGGCACTACCTGGAATCTACAATCGAGCCCTTCAAGAT
CCTGACCGACCACAGAAACCTGATTGGAAGAATCACAAACGAGAGCGAACCAGAG
AACAAGCGGCTGGCCAGATGGCAGCTGTTCCTGCAGGACTTCAACTTCGAGATCA
ACTACAGACCTGGCTCTGCAAATCACATCGCCGATGCCCTGTCTAGAATCGTGGA
CGAGACTGAGCCTATCCCTAAGGACAACGAAGATAACAGCATCAACTTCGTGAACC
AGATCAGCATCTCTGGCGGCTCAAAAAGAACCGCCGACGGCAGCGAATTCGAGTC
TCCCAAGAAGAAGAGGAAAGTCGGCTCTGGCCCTGCCGCTAAGAGAGTGAAGCTG
GACTAATTAATTAAGCTGCCTTCTGCGGGGCTTGCCTTCTGGCCATGCCCTTCTTC
TCTCCCTTGCACCTGTACCTCTTGGTCTTTGAATAAAGCCTGAGTAGGAAG

Supplementary Sequence 4. PE6c without RT for untethered PE, 4428 bp

Inactive T7 promoter-5’ UTR-SV40NLS-PE6c no RT-SV40NLS-cMycNLS-3’ UTR

TAATACGACACACTATAAGGAAATAAGAGAGAAAAGAAGAGTAAGAAGAAATATAA
GAGCCACCATGAAACGGACAGCCGACGGAAGCGAGTTCGAGTCACCAAAGAAGA
AGCGGAAAGTCGACAAGAAGTACAGCATCGGCCTGGACATCGGCACCAACTCTGT
GGGCTGGGCCGTGATCACCGACGAGTACAAGGTGCCCAGCAAGAAATTCAAGGT
GCTGGGCAACACCGACCGGCACAGCATCAAGAAGAACCTGATCGGAGCCCTGCT
GTTCGACAGCGGCGAAACAGCCGAGGCCACCCGGCTGAAGAGAACCGCCAGAAG
AAGATACACCAGACGGAAGAACCGGATCTGCTATCTGCAAGAGATCTTCAGCAAC
GAGATGGCCAAGGTGGACGACAGCTTCTTCCACAGACTGGAAGAGTCCTTCCTGG
TGGAAGAGGATAAGAAGCACGAGCGGCACCCCATCTTCGGCAACATCGTGGACGA
GGTGGCCTACCACGAGAAGTACCCCACCATCTACCACCTGAGAAAGAAACTGGTG
GACAGCACCGACAAGGCCGACCTGCGGCTGATCTATCTGGCCCTGGCCCACATG
ATCAAGTTCCGGGGCCACTTCCTGATCGAGGGCGACCTGAACCCCGACAACAGCG
ACGTGGACAAGCTGTTCATCCAGCTGGTGCAGACCTACAACCAGCTGTTCGAGGA
AAACCCCATCAACGCCAGCGGCGTGGACGCCAAGGCCATCCTGTCTGCCAGACT
GAGCAAGAGCAGAAAGCTGGAAAATCTGATCGCCCAGCTGCCCGGCGAGAAGAA
GAATGGCCTGTTCGGAAACCTGATTGCCCTGAGCCTGGGCCTGACCCCCAACTTC
AAGAGCAACTTCGACCTGGCCGAGGATGCCAAACTGCAGCTGAGCAAGGACACCT
ACGACGACGACCTGGACAACCTGCTGGCCCAGATCGGCGACCAGTACGCCGACC
TGTTTCTGGCCGCCAAGAACCTGTCCGACGCCATCCTGCTGAGCGACATCCTGAG
AGTGAACACCGAGATCACCAAGGCCCCCCTGAGCGCCTCTATGATCAAGAGATAC
GACGAGCACCACCAGGACCTGACCCTGCTGAAAGCTCTCGTGCGGCAGCAGCTG
CCTGAGAAGTACAAAGAGATTTTCTTCGACCAGAGCAAGAACGGCTACGCCGGCT
ACATTGACGGCGGAGCCAGCCAGGAAGAGTTCTACAAGTTCATCAAGCCCATCCT
GGAAAAGATGGACGGCACCGAGGAACTGCTCGTGAAGCTGAAGAGAGAGGACCT
GCTGCGGAAGCAGCGGACCTTCGACAACGGCAGCATCCCCCACCAGATCCACCT
GGGAGAGCTGCACGCCATTCTGCGGCGGCAGGAAGATTTTTACCCATTCCTGAAG
GACAACCGGGAAAAGATCGAGAAGATCCTGACCTTCCGCATCCCCTACTACGTGG
GCCCTCTGGCCAGGGGAAACAGCAGATTCGCCTGGATGACCAGAAAGAGCGAGG
AAACCATCACCCCCTGGAACTTCGAGGAAGTGGTGGACAAGGGCGCTTCCGCCCA
GAGCTTCATCGAGCGGATGACCAACTTCGATAAGAACCTGCCCAACGAGAAGGTG
CTGCCCAAGCACAGCCTGCTGTACGAGTACTTCACCGTGTATAACGAGCTGACCA
AAGTGAAATACGTGACCGAGGGAATGAGAAAGCCCGCCTTCCTGAGCGGCGAGC
AGAAAAAGGCCATCGTGGACCTGCTGTTCAAGACCAACCGGAAAGTGACCGTGAA
GCAGCTGAAAGAGGACTACTTCAAGAAAATCGAGTGCTTCGACTCCGTGGAAATCT
CCGGCGTGGAAGATCGGTTCAACGCCTCCCTGGGCACATACCACGATCTGCTGAA
AATTATCAAGGACAAGGACTTCCTGGACAATGAGGAAAACGAGGACATTCTGGAAG

ATATCGTGCTGACCCTGACACTGTTTGAGGACAGAGAGATGATCGAGGAACGGCT
GAAAACCTATGCCCACCTGTTCGACGACAAAGTGATGAAGCAGCTGAAGCGGCGG
AGATACACCGGCTGGGGCAGGCTGAGCCGGAAGCTGATCAACGGCATCCGGGAC
AAGCAGTCCGGCAAGACAATCCTGGATTTCCTGAAGTCCGACGGCTTCGCCAACA
GAAACTTCATGCAGCTGATCCACGACGACAGCCTGACCTTTAAAGAGGACATCCA
GAAAGCCCAGGTGTCCGGCCAGGGCGATAGCCTGCACGAGCACATTGCCAATCT
GGCCGGCAGCCCCGCCATTAAGAAGGGCATCCTGCAGACAGTGAAGGTGGTGGA
CGAGCTCGTGAAAGTGATGGGCCGGCACAAGCCCGAGAACATCGTGATCGAAATG
GCCAGAGAGAACCAGACCACCCAGAAGGGACAGAAGAACAGCCGCGAGAGAATG
AAGCGGATCGAAGAGGGCATCAAAGAGCTGGGCAGCCAGATCCTGAAAGAACAC
CCCGTGGAAAACACCCAGCTGCAGAACGAGAAGCTGTACCTGTACTACCTGCAGA
ATGGGCGGGATATGTACGTGGACCAGGAACTGGACATCAACCGGCTGTCCGACTA
CGATGTGGACGCTATCGTGCCTCAGAGCTTTCTGAAGGACGACTCCATCGACAAC
AAGGTGCTGACCAGAAGCGACAAGAACCGGGGCAAGAGCGACAACGTGCCCTCC
GAAGAGGTCGTGAAGAAGATGAAGAACTACTGGCGGCAGCTGCTGAACGCCAAG
CTGATTACCCAGAGAAAGTTCGACAATCTGACCAAGGCCGAGAGAGGCGGCCTGA
GCGAACTGGATAAGGCCGGCTTCATCAAGAGACAGCTGGTGGAAACCCGGCAGAT
CACAAAGCACGTGGCACAGATCCTGGACTCCCGGATGAACACTAAGTACGACGAG
AATGACAAGCTGATCCGGGAAGTGAAAGTGATCACCCTGAAGTCCAAGCTGGTGT
CCGATTTCCGGAAGGATTTCCAGTTTTACAAAGTGCGCGAGATCAACAACTACCAC
CACGCCCACGACGCCTACCTGAACGCCGTCGTGGGAACCGCCCTGATCAAAAAGT
ACCCTAAGCTGGAAAGCGAGTTCGTGTACGGCGACTACAAGGTGTACGACGTGCG
GAAGATGATCGCCAAGAGCGAGCAGGAAATCGGCAAGGCTACCGCCAAGTACTTC
TTCTACAGCAACATCATGAACTTTTTCAAGACCGAGATTACCCTGGCCAACGGCGA
GATCCGGAAGCGGCCTCTGATCGAGACAAACGGCGAAACCGGGGAGATCGTGTG
GGATAAGGGCCGGGATTTTGCCACCGTGCGGAAAGTGCTGAGCATGCCCCAAGT
GAATATCGTGAAAAAGACCGAGGTGCAGACAGGCGGCTTCAGCAAAGAGTCTATC
CTGCCCAAGAGGAACAGCGATAAGCTGATCGCCAGAAAGAAGGACTGGGACCCTA
AGAAGTACGGCGGCTTCGACAGCCCCACCGTGGCCTATTCTGTGCTGGTGGTGGC
CAAAGTGGAAAAGGGCAAGTCCAAGAAACTGAAGAGTGTGAAAGAGCTGCTGGGG
ATCACCATCATGGAAAGAAGCAGCTTCGAGAAGAATCCCATCGACTTTCTGGAAGC
CAAGGGCTACAAAGAAGTGAAAAAGGACCTGATCATCAAGCTGCCTAAGTACTCCC
TGTTCGAGCTGGAAAACGGCCGGAAGAGAATGCTGGCCTCTGCCGGCGAACTGC
AGAAGGGAAACGAACTGGCCCTGCCCTCCAAATATGTGAACTTCCTGTACCTGGC
CAGCCACTATGAGAAGCTGAAGGGCTCCCCCGAGGATAATGAGCAGAAACAGCTG
TTTGTGGAACAGCACAAGCACTACCTGGACGAGATCATCGAGCAGATCAGCGAGT
TCTCCAAGAGAGTGATCCTGGCCGACGCTAATCTGGACAAAGTGCTGTCCGCCTA
CAACAAGCACCGGGATAAGCCCATCAGAGAGCAGGCCGAGAATATCATCCACCTG
TTTACCCTGACCAATCTGGGAGCCCCTGCCGCCTTCAAGTACTTTGACACCACCAT
CGACCGGAAGAGGTACACCAGCACCAAAGAGGTGCTGGACGCCACCCTGATCCA

CCAGAGCATCACCGGCCTGTACGAGACACGGATCGACCTGTCTCAGCTGGGAGG
TGACTCTGGCGGCTCAAAAAGAACCGCCGACGGCAGCGAATTCGAGTCTCCCAAG
AAGAAGAGGAAAGTCGGCTCTGGCCCTGCCGCTAAGAGAGTGAAGCTGGACTAAT
TAATTAAGCTGCCTTCTGCGGGGCTTGCCTTCTGGCCATGCCCTTCTTCTCTCCCT
TGCACCTGTACCTCTTGGTCTTTGAATAAAGCCTGAGTAGGAAG

Supplementary Sequence 5. eeTf1 RT from PE6c with N-terminal MCP fusion for untethered PE, 2238 bp

Inactive T7 promoter-5' UTR-MCP-XTEN-eeTF1 RT-SV40NLS-3' UTR

TAATACGACACACTATAAGGAAATAAGAGAGAAAAGAAGAGTAAGAAGAAATATAA
GAGCCACCATGGCTTCAAACTTTACTCAGTTCGTGCTCGTGGACAATGGTGGGAC
AGGGGATGTGACAGTGGCTCCTTCTAATTTCGCTAATGGGGTGGCAGAGTGGATC
AGCTCCAACTCACGGAGCCAGGCCTACAAGGTGACATGCAGCGTCAGGCAGTCTA
GTGCCCAGAAGAGAAAGTATACCATCAAGGTGGAGGTCCCCAAAGTGGCTACCCA
GACAGTGGGCGGAGTCGAACTGCCTGTCGCCGCTTGGAGGTCCTACCTGAACAT
GGAGCTCACTATCCCAATTTTCGCTACCAATTCTGACTGTGAACTCATCGTGAAGG
CAATGCAGGGGCTCCTCAAAGACGGTAATCCTATCCCTTCCGCCATCGCCGCTAA
CTCAGGTATCTACTCTGGAGGATCTAGCGGAGGATCCTCTGGCAGCGAGACACCA
GGAACAAGCGAGTCAGCAACACCAGAGAGCAGTGGCGGCAGCAGCGGCGGCAG
CAGCATCAGCAGCTCTAAGCACACCCTGAGCCAGATGAACAAGGTGAGCAACATC
GTGAAGGAACCCGAGCTGCCCGACATCTACAAGGAATTTAAGGACATCACCGCCG
ACACCAATACCGAGAAGCTGCCTAAGCCTATTAAGGGCCTGGAATTTGAAGTGGAA
CTGACACAGGAGAACTACAGACTGCCTATCCGGAACTATCCTCTGACTCCAGTCAA
GATGCAGGCCATGAACGACGAGATCAATCAAGGCCTGAAAGGCGGCATCATCAGA
GAGAGCAAGGCCATCAACGCCTGCCCTGTTATATTCGTGCCCAGAAAGGAAGGCA
CACTGCGCATGGTGGTCGACTACAGGCCCCTGAACAAGTACGTGAAGCCCAACGT
CTACCCCCTGCCACTGATTGAACAACTGCTGGCCAAGATCCAGGGCAGCACCATT
TTCACCAAGCTGGACCTGAAAAGCGCCTACCACCAGATCAGAGTGCGAAAGGGAG
ATGAGCACAAGCTGGCCTTCCGGTGCCCCAGAGGAGTCTTCGAGTACCTGGTGAT
GCCTTACGGCATCAAGACAGCCCCTGCCCACTTTCAGTACTTCATCAACACAATCC
TGGGCGAGGCCAAGGAAAGCCACGTGGTGTGCTACATGGACGACATCCTGATCCA
TTCCAAGTCCGAGTCCGAACACGTGAAACATGTGAAGGACGTGCTGCAAAAGCTG
AAGAACGCTAATCTGATCATCAACCAGGCCAAATGCGAGTTTCACCAGAGCCAAGT
GAAGTTCCTGGGCTACCACATCAGCGAGAAGGGCTTAACCCCATGTCAGGAGAAC
ATCGACAAGGTGCTGCAGTGGAAACAGCCTAAAAACCAGAAGGAACTGAGACAGT
TCCTGGGCCAGGTGAACTACCTGAGAAAATTCATCCCCAAAACCAGCCAGTTGAC
CCACCCTCTGAACAAACTCCTGAAAAAGGATGTCAGATGGAAATGGACCCCTACCC
AGACCCAGGCTATCGAGAATATCAAGCAATGTCTGGTGTCTCCTCCTGTGCTGAG
GCACTTCGACTTCAGCAAGAAGATCCTGCTTGAGACAGACGTTTCTGATGTGGCC
GTGGGAGCCGTGCTGAGCCAGAAGCATGATGATGATAAGTACTACCCTGTGGGCT
ATTACAGCGCTAAAATGAGCAAAGCCCAGCTGAATTATTCTGTGTCCGACAAGGAG
ATGCTGGCTATCATCAAAAGCCTGGAGCACTGGCGGCACTACCTGGAATCTACAAT
CGAGCCCTTCAAGATCCTGACCGACCACAGAAACCTGATTGGAAGAATCACAAAC
GAGAGCGAACCAGAGAACAAGCGGCTGGCCAGATGGCAGCTGTTCCTGCAGGAC

TTCAACTTCGAGATCAACTACAGACCTGGCTCTGCAAATCACATCGCCGATGCCCT
GTCTAGAATCGTGGACGAGACTGAGCCTATCCCTAAGGACAACGAAGATAACAGC
ATCAACTTCGTGAACCAGATCAGCATCTCTGGCGGCTCAAAAAGAACCGCCGACG
GCAGCGAATTCGAGTCTCCCAAGAAGAAGAGGAAAGTCTAATTAATTAAGCTGCCT
TCTGCGGGGCTTGCCTTCTGGCCATGCCCTTCTTCTCTCCCTTGCACCTGTACCTC
TTGGTCTTTGAATAAAGCCTGAGTAGGAAG

Supplementary Sequence 6. Lentiviral cassette harboring the *PAH* R408W variant

Lentiviral cassette containing an array of mutated loci associated with genetic metabolic diseases along with 50 bp of flanking endogenous sequence on each side of the variants.

PAH P281L – *PAH* R408W – *MMUT* R369H – *MMUT* R727X - *MMAB* R186W – *MMUT* R108C – *ASS1* G390R

CGTCTCACACCTTTTTTTAGATGGCGCTCATTGTGCCTGGCAACTGGTAGCTGGAG
GACAGTACTCACAGTTCGGGGGTATACATGGGCTTGGATCCATGTCTGATGTACTG
TGTGCAGCAAGACCTCAATCCTTTGGGTGTATGGGTCGTAGCGAACTGAGAAGGG
CCAAGGTATTGTGGCAGCAAAGTTCCTAAGACCAAAACCACAGGCTTGAGTGAGC
AAAGACTGAGTCCCTCCAAATACTGCTGCCATTGCTTCTATTGCAGTATGGACAAT
ATTATTGTAGGGATCCTAAAATATTTGATAAAAAACAAAAACTCAAACACTTCTCAAT
ATCATCAAGCACCTGAACGGCAGCCTTTGGAATTCAAGTCCCAGGACCAAATACAT
TGGAAACACCAACTTCAAACAGAAATTCATCTTTCTCCTTTCAGTCGGGAGGCAAG
ATCAGCTCGGCGCTGCATTTCTGCTGGGCCGTGTGCCGCCGGGCCGAGAGACGG
TAAGAGGGCTGGAGAGAGGGATCACACGTGGACCATATCCTACCATGTATACCTTT
AGGCCCTGGACCATCTGCCAGTATGCTGGTTTTAGTACTGTGGAAGAAAGCAATAA
GTTCTATAAGCTCCTTGCAGCATGAACGTGCAGGGTGATTATGAGCCAACTGATGC
CACCAGGTTCATCAACATCAATTCCCTCAGGTGAGAAGCTCAGGGCCCTGACGGG
TTTTGAGACG

Supplementary References

1. Li, S. et al. Payload distribution and capacity of mRNA lipid nanoparticles. *Nature Communications* **13** (2022).
2. Davis, J.R. et al. Efficient prime editing in mouse brain, liver and heart with dual AAVs. *Nature Biotechnology* **42**, 253–264 (2023).
3. Liu, B. et al. A split prime editor with untethered reverse transcriptase and circular RNA template. *Nature Biotechnology* **40**, 1388–1393 (2022).
4. An, M. et al. Engineered virus-like particles for transient delivery of prime editor ribonucleoprotein complexes in vivo. *Nature Biotechnology* **42**, 1526–1537 (2024).
5. Kauffman, K.J. et al. Optimization of Lipid Nanoparticle Formulations for mRNA Delivery in Vivo with Fractional Factorial and Definitive Screening Designs. *Nano Letters* **15**, 7300–7306 (2015).
6. Fenton, O.S. et al. Bioinspired Alkenyl Amino Alcohol Ionizable Lipid Materials for Highly Potent In Vivo mRNA Delivery. *Advanced Materials* **28**, 2939–2943 (2016).
7. Miao, L. et al. Synergistic lipid compositions for albumin receptor mediated delivery of mRNA to the liver. *Nature Communications* **11** (2020).
8. Rudra, A. et al. Degradable cyclic amino alcohol ionizable lipids as vectors for potent influenza mRNA vaccines. *Nature Nanotechnology* **20**, 1831–1842 (2025).
9. Sabnis, S. et al. A Novel Amino Lipid Series for mRNA Delivery: Improved Endosomal Escape and Sustained Pharmacology and Safety in Non-human Primates. *Molecular Therapy* **26**, 1509–1519 (2018).
